# The prediction of swarming in honeybee colonies using vibrational spectra

**DOI:** 10.1038/s41598-020-66115-5

**Published:** 2020-06-16

**Authors:** Michael-Thomas Ramsey, Martin Bencsik, Michael Ian Newton, Maritza Reyes, Maryline Pioz, Didier Crauser, Noa Simon Delso, Yves Le Conte

**Affiliations:** 1 Nottingham Trent University, School of Science and Technology, Clifton Lane, Nottingham, NG11 8NS United Kingdom; 2 l’Institut National de Recherche en Agriculture, Alimentation et Environnement (INRAE), UR 406 Abeilles et Environnement, Domaine Saint-Paul, 84914 Avignon, France; 3 Centre Apicole de Recherche et d’Information, CARI, 4, Place Croix du Sud, B-1348 Louvain-La-Neuve, Belgium

**Keywords:** Computational biophysics, Biophysics, Mathematics and computing, Software, Population dynamics

## Abstract

In this work, we disclose a non-invasive method for the monitoring and predicting of the swarming process within honeybee colonies, using vibro-acoustic information. Two machine learning algorithms are presented for the prediction of swarming, based on vibration data recorded using accelerometers placed in the heart of honeybee hives. Both algorithms successfully discriminate between colonies intending and not intending to swarm with a high degree of accuracy, over 90% for each method, with successful swarming prediction up to 30 days prior to the event. We show that instantaneous vibrational spectra predict the swarming within the swarming season only, and that this limitation can be lifted provided that the history of the evolution of the spectra is accounted for. We also disclose queen toots and quacks, showing statistics of the occurrence of queen pipes over the entire swarming season. From this we were able to determine that (1) tooting always precedes quacking, (2) under natural conditions there is a 4 to 7 day period without queen tooting following the exit of the primary swarm, and (3) human intervention, such as queen clipping and the opening of a hive, causes strong interferences with important mechanisms for the prevention of simultaneous rival queen emergence.

## Introduction

Collective animal behaviour is a subdivision of social behaviour focussed on the coordination and emergent properties of large groups of animals achieved through group-wide transfer of information, group decision-making, group locomotion and synchronization of activities. Many forms of collective behaviour can be observed across multiple animal groups: murmurations of European starlings (*Sturnus vulgaris*)^1^, twisting and turning in complete unison; schools of Atlantic chub mackerel (*Scomber colias*), that split and reform to confuse predators^2^; the thousands of wildebeest that migrate across the plains of East Africa in search of better grazing^3^; and, most relevant to this paper, the swarms of honeybees in search of a new home that settle on tree branches and debate on the location of their future nest-site^4^.

In basic terms, swarming in honeybee colonies is a system of reproduction^5^. This process comprises of a complex array of collective behaviours that have evolved to increase the probability of reproductive success: the splitting of one colony into several new viable ones. Particularly in *Apis mellifera* adapted to temperate climates, the swarming season extends across the late spring and early summer months^6^. In this time, a colony’s population will expand until it is too large for its maternal nest, at which point the colony will proceed to divide itself through the swarming process^5^. Seeley *et al*.^5^ states that ‘*Mature colonies have a natural urge to swarm each year unless weakened by disease or mismanagement*‘. In the first swarming instance of the season, known as the primary swarm, the old queen and approximately half the worker population (of all ages) leave the parental colony to establish a new colony, leaving behind a series of virgin daughter queens (gynes) encapsulated in the cells in which they developed, and the remaining workers, to perpetuate the old colony. Additionally, it is common that one or more afterswarms will ensue with a large proportion of bees leaving with virgin queens after they emerge from their cells. The swarming bees will quickly exit the hive *en masse* and retain a cloud formation for around twenty minutes until they amalgamate in a beard-like cluster at a temporary site (usually a nearby tree branch) where they decide on their future nest-site^7^.

Amongst the vast array of signals, cues and behaviours, important vibro-acoustic information is conveyed within the colony throughout some of the swarming season. This is particularly obvious from the piping of virgin queens. Queen rearing is thought to be the stimulus for engorgement prior to swarming^6^. Once the mated queen leaves with the primary swarm, the first virgin queen will be released from her cell between a few hours and up to ten days after, depending on her stage in development when the primary swarm occurred^6^. Following hatching, the newly emerged virgin queen will produce a type of piping known as “tooting” or “toots”, which is thought to announce her arrival to the colony^6^. During each tooting sequence, the queen presses her thorax onto the comb and vibrates her wing muscles in the folded position^8^. Typically, the emerged queen produces several one-second-long pipes that are each immediately followed by several bursts of less than half a second^9^ with a fundamental frequency that increases with the age of queens, ranging from 200 to 550 Hz^10^, and is usually observed around 400 Hz. Mature virgin queens still confined within their queen cells respond to this tooting with their own distinct piping sound, referred to as “quacking” or “quacks”, which probably alerts the emerged queen and the workers of their presence^6^. Quacking is a sequence of numerous short pulses that each typically last less than 0.2 seconds^11^ at a lower fundamental frequency compared to tooting, of around 350 Hz. Both types of queen piping are broadcast within the hive as vibrations through the honeycomb^10^. The function of the tooting was examined by Grooters^12^ who demonstrated that a single replay of tooting inhibited the emergence of mature encapsulated gynes engaged in chewing their way out of their cell, delaying this behaviour for several hours. This then enabled the worker bees to reseal the cells to obstruct their emergence for up to several days^12^. Due to limitations in our ability to observe honeybees under natural conditions, all such experiments to date have been conducted under controlled conditions. Whilst this gives great insight into the proposed functions of the signals, our long-term, non-invasive measurements provide a more ethological method for the *in-situ* monitoring of honeybee hives, providing valuable continuous recordings of queen pipes occurring under natural conditions.

Most of the vibrational and acoustic information emanating from beehives is a by-product of the bees’ activities^13,14^ and not an outcome of direct communication signals, such as the whooping^15^ and the DVAV signal^16^, however, this does not prevent possible high biological significance. Experienced beekeepers sometimes claim the ability to identify certain conditions within a hive, solely by listening to its hum. In fact, the concept that swarming can be predicted by acoustic measurements can be traced back as early as Aristotle, who wrote: *“When the flight of a swarm is imminent, a monotonous and quite peculiar sound made by all the bees is heard for several days*”^17^. As such, acoustic signals have been targeted in some studies for honeybee swarm prediction. In the mid twentieth century, Woods^18^ obtained the sound spectrum of three colonies of healthy bees using a standard wave analyser. He showed modulation of a 250 Hz component that he termed “the warble” in relation to the status of the colony, particularly during the preparation for swarming. By placing microphones on the top of the brood frames within a beehive, Ferarri *et al*.^19^ were also able to show some evidence of frequency shifts during the swarming process. Similarly, using microphones placed close to the brood chamber, Vancata^20^ suggested that the intensity of the spectrum across a frequency range of 200–500 Hz changes within the 21 days leading up to the primary swarm. However, due to the nature of the equipment, microphone technology tends to only provide crude short-term data collection (e.g. Woods^18^). In a recent paper by Zgank^21^, an Internet of Things based bee activity acoustic classification system is presented, with acoustic training data being collected from the Open Source Beehives Project. However, in contrast to this paper, which aims to predict whether a honeybee colony is preparing to swarm or not, their work focused only on identifying the best machine learning algorithm for acoustic classification between the two states, preparing and not preparing. Owing to the use of training data originating from only one hive, this categorisation would not be suitable for application across multiple hives^21^. Outside of the focus of swarming, acoustic measurements have also been explored to make assessments into the general health status of honeybee colonies. Robles-Guerrero *et al*.^22^ compared the sounds emanating from a healthy colony to those of a colony that had been artificially made queenless, and Qandaur *et al*.^23^ compared healthy and Varroa-infested hives. In both cases, preliminary results demonstrate some ability to computationally discriminate between colony conditions based on vibro-acoustic information, but always only for a limited number of colonies.

Previous work^24^ focused on swarming prediction have also employed temperature sensors placed above the brood body within a beehive to predict specific annual phases in honeybee colony development, with suggestions that substantial prolonged temperature decreases could be the result of swarming or queen replacement. Ferrari *et al*.^19^, alongside their acoustic measurements detailed above, also showed a reduction in hive temperature immediately prior to each swarming event measured across nine swarms issuing from three hives. However, this difference in temperature occurred just minutes before the swarming event and would therefore not provide a useful tool for swarming prediction. Buchmann and Thoenes^25^ and Meikle *et al*.^26^ also showed that the weight of the hive measured with electronic scales can reveal weight changes in colonies in relation to swarming. However, the subsequent rapid weight change can only reveal if and when the colony has already swarmed; it gives no indication as to its swarming preparations.

The above methods, as well as our own technique, contribute to “precision apiculture”, which is defined as any honeybee management strategy that involves the monitoring of individual colony parameters (such as: temperature, humidity, sound, vibration and weight) to minimise the beekeeper’s expended resources (such as: time and money) and maximize the hive’s productivity^27^. With respect to the swarming process, if a system were in place that could identify a colony’s intent to swarm, a beekeeper would benefit from being alerted to those hives preparing to swarm and would prioritise the appropriate swarm management procedures to those hives, reducing the need and burden for every colony to be regularly inspected, often unnecessarily. In line with Bencsik *et al*.^13^, this study will, therefore, focus on two strategies that utilise continuous vibrational information taken from colonies preparing and not preparing to swarm in order to predict the swarming process in honeybee colonies. The aim of this work is to explore whether there are features found in the honeycomb vibrational spectra that are specific to colonies’ preparations to produce a primary swarm. These features are then used to raise an alarm, as early as possible prior to the event. This technique encompasses the use of accelerometers placed directly in the centre of honeybee hives and continuously collects the average spectra of vibration at three minutes intervals (see Methods). A reliable signature will be one which will detect a high proportion of swarms. A more reliable one will work for swarm events that have not contributed to the numerical search (training stage) originally required to clarify the signature. The best vibrational signature will be one that will work from season to season, and for different apiaries. Usually the primary swarm is not preceded by any piping, but nevertheless we have also endeavoured, in this study, to carefully examine the pipes logged within our continuous vibrational datasets, as it seems to strongly support the theory that tooting and quacking are produced to inform the worker bees about the need to release or keep captive un-emerged virgin queens.

## Results

### Prediction of swarming

**Figure 1:**
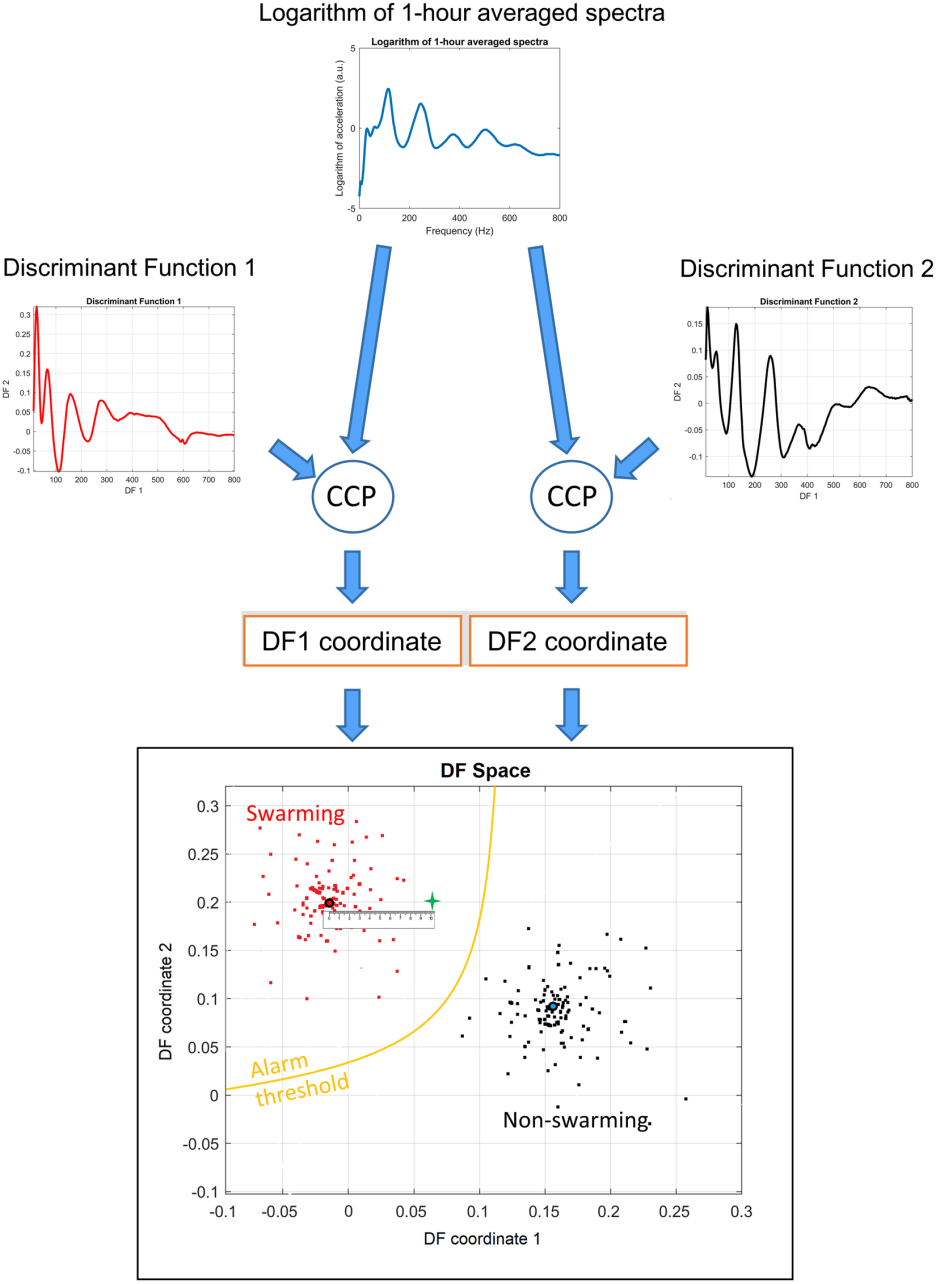
The instantaneous alarm procedure. The images in Fig. 1 are solely for illustrative purposes. This figure depicts the processing for the detection of swarming in the post-training phase. Each hour-long averaged spectrum is compared, using the cross-correlation product (CCP), to a pair of DF curves, as determined by the DFA algorithm (see Fig. 2), providing the co-ordinates for a single point in the DF space (green ‘+’ sign). The Euclidean distance to the swarming and non-swarming centroids (the central point of all swarming and non-swarming spectra, used in the training phase, respectively shown as a red dot with black outline and green dot with blue outline) are calculated. The ratio of these distances provides our “criterion value”, and is compared to an optimised threshold value (see Methods) to determine if it is the spectrum of a colony intending to swarm or not, i.e. if the point in DF space lies within the upper left “swarming area” bordered by the yellow line. In this figure, for ease of representation, we give an example using two discriminant functions, however, in this study, three discriminant functions were used. Plots used in the creation of this figure were generated using the MATLAB (R2018b) software (uk.mathworks.com).

**Figure 2:**
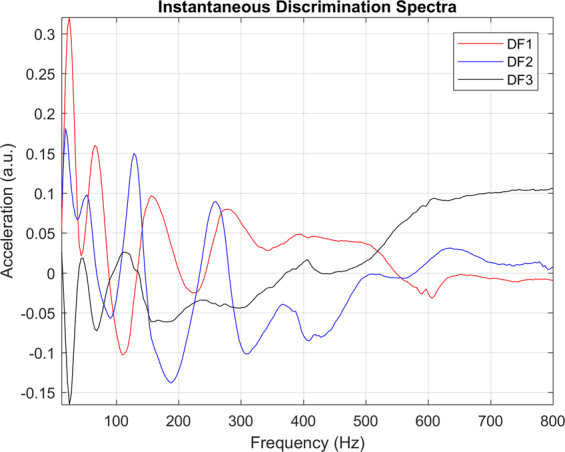
The optimised instantaneous discrimination spectra (DF1, DF2 and DF3) as defined by the DFA algorithm. Frequency along the X-axis is cropped between 18 and 800 Hz; the Y-axis shows the logarithm of acceleration. High (positive or negative) acceleration values indicate spectral areas of particular importance to swarming prediction, whilst low values, approaching zero, correspond to vibrational frequencies which must be dismissed. This figure was created using the MATLAB (R2018b) software (uk.mathworks.com).

**Figure 3:**
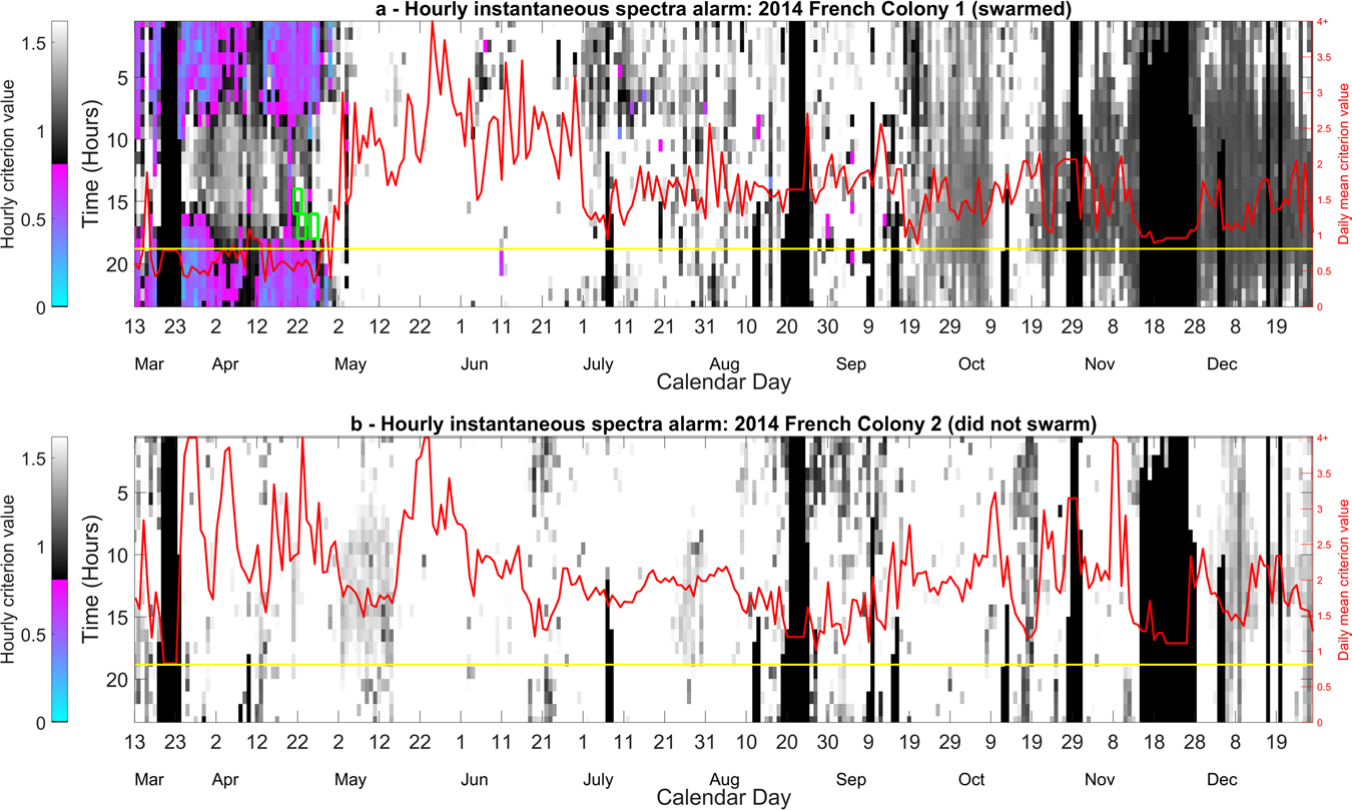
Time course of the swarming alert for a specific colony that (**a**) swarmed and (**b**) did not swarm, using the DF spectra in Fig. 2. The instantaneous alarm (computed without any contribution coming from past data) for these two colonies was monitored from the 13^th^ March until 1^st^ December 2014. The colour coding has been split to clearly convey two very different pieces of information. Greyscale tones correspond to the alarm values above the threshold (non-swarming state) and the remaining colour emphasises the alarm values below the threshold (swarming state), from pink to blue as the criterion approaches the swarming centroid. Green rectangles show the occurrence of three swarms within this dataset, with the earliest one being the primary swarm. Superimposed is another, red curve, with its own Y-axis shown on the extreme right, showing the average of the previous night’s alarm values taken between midnight and 5:00 AM. The yellow line displays the alarm threshold. Note that the black sections seen within the hourly alarm plots are the result of missing data, due to power cuts. This figure was created using the MATLAB (R2018b) software (uk.mathworks.com).

**Figure 4:**
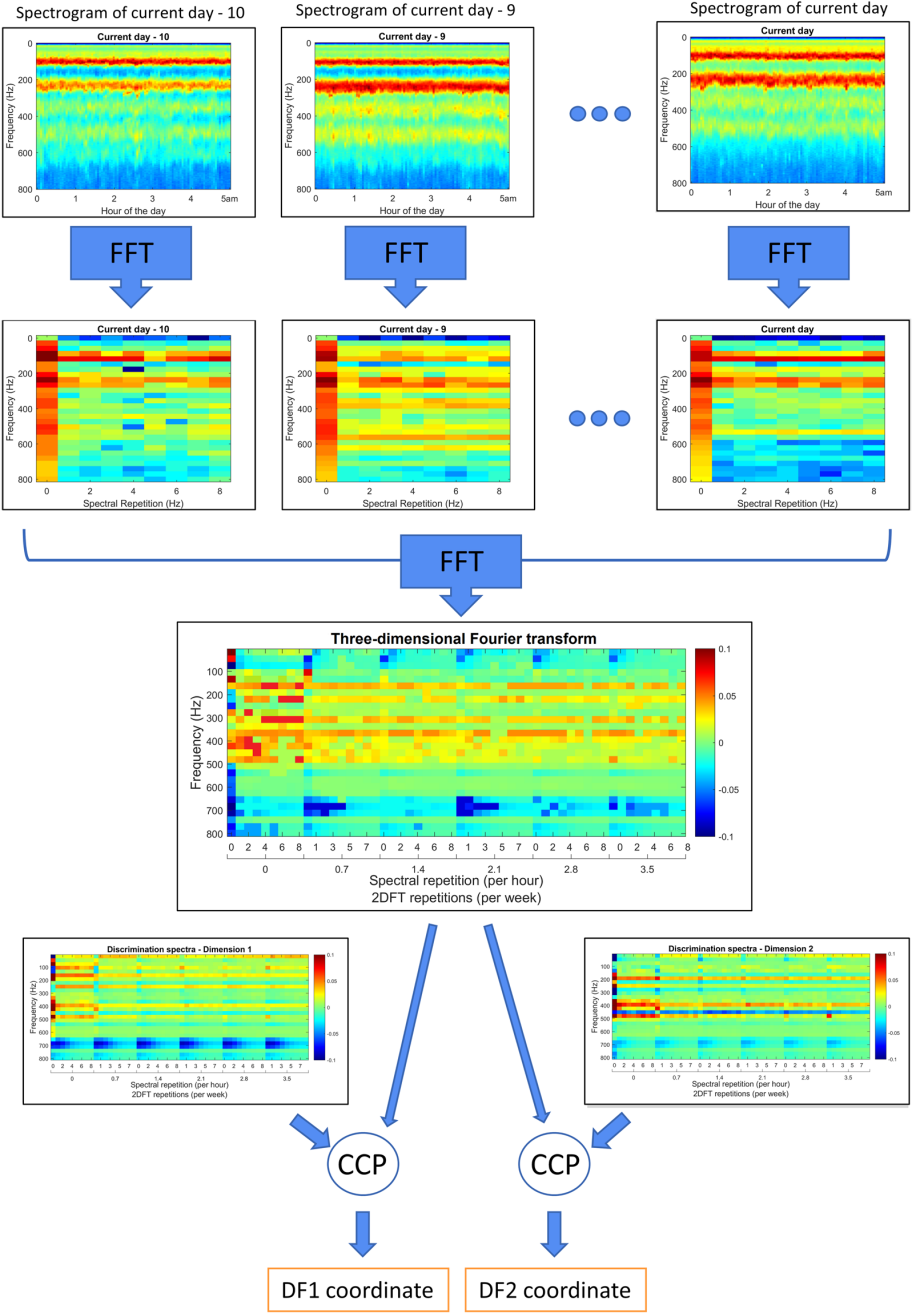
The spectral evolution alarm procedure. The images here are solely for illustrative purposes. This figure depicts the process for the detection of swarming in the post-training phase. In order to account for the history of the vibrational spectra, the FFT (Fast Fourier Transform) is performed on the time course of the magnitude of all the uploaded spectral frequencies for each day between midnight and 5:00 AM, yielding 2DFT (two dimensional Fourier Transform) images (second line). The FFT is then further calculated over each pixel of the series of 2DFTs found in the preceding days (third line). Each 3DFT (three dimensional Fourier Transform) is compared to a pair of Discriminant Functions, as determined by the DFA algorithm (Fig. 5), using the cross-correlation product (CCP), the values of which determine the co-ordinates for a single point in the DF space. The remainder of the procedure is identical to that described in Fig. 1. In this figure, for ease of representation, we give an example using two discriminant functions, however, in this study, three discriminant functions were used. Plots used in the creation of this figure were generated using the MATLAB (R2018b) software (uk.mathworks.com).

**Figure 5:**
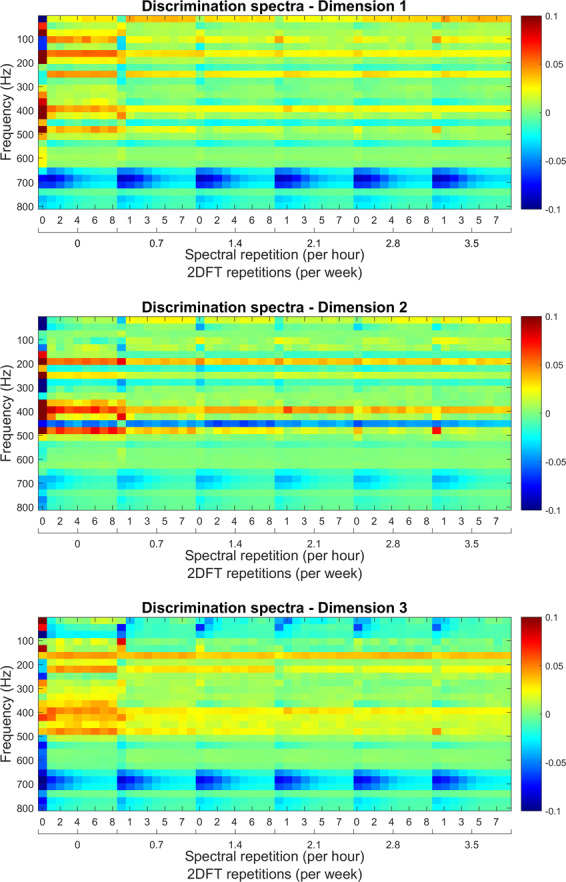
The three discrimination spectra for the spectral evolution algorithm. The pixel colour indicates the negative (blue), positive (red) or zero (green) contribution the user must give to a particular part of the vibrational 3DFT in order to get the first (top), second (middle) or third (bottom) DF score. The frequency components of the FFT of the 2DFTs are shown stacked from left (0 repetitions per week) to right (3.5 repetitions per week) for ease of viewing. This figure was created using the MATLAB (R2018b) software (uk.mathworks.com).

Two strategies are presented here that both use averaged vibrational spectra for the prediction of swarming: the first (Fig. 1), utilises discriminant functions created from instantaneous (dismissing the influence of any past data) averaged vibrational spectra (Fig. 2), which only performed well when restrictively applied over the swarming season (Fig. 3), and a second (Fig. 4) that uses three dimensional Fourier analysis (3DFT) to account for the evolution of the previous ten days’ spectra. For both strategies, in the first instance, a numerical search was required to define those Discriminant Functions (Fig. 2 for instantaneous functions and Fig. 5 for spectral evolution functions) that best enable the classification of vibrational spectra as being that of a colony in a state of intending to swarm or not, as described for each strategy in Figs. 1 and 4. In-line with Ramsey *et al*.^15,16^, this numerical search consists of successively applying a data reduction exercise (Principal Component Analysis, PCA) followed by a supervised clustering exercise (Discriminant Function Analysis, DFA) on the selection of PCA scores. To best enable this to identify those measurements that correspond to (i) colonies preparing to swarm and (ii) colonies not preparing to swarm, specific measurements that exhibited the most generic features for these two conditions (see Bisele, *et al*.^28^) were supplied as ‘training data’. For more information on how the two conditions were identified and discriminated, refer to the Methods.

### Strategy 1 – Discrimination of instantaneous spectra

From a possible 253 one-hour-long averaged spectra from colonies *preparing to swarm* (PTS) and 1754 from *non-swarming* (NS) colonies (see Methods), parametric optimisation (see Methods) revealed that the best performance from the software was obtained when using a training database size of 24 PTS and 32 NS 3-minute averaged logarithmic spectra. These are further averaged between 3:00 and 4:00 AM over the 13 days leading up to the primary swarm and were cropped over a bandwidth of 18 to 800 Hz, with 40 percent of the PCA scores being projected onto three DF scores (Fig. 2). These parameters yielded a 6.3% classification error (percentage of misclassified averaged spectra over the entire collection of available data). The three discriminant spectra in Fig. 2, resulting from our numerical search, have very different spectral features, and they reveal the highest contribution to originate at around 20 Hz, with DF3 exhibiting the majority of its overall information at 24 Hz and its harmonic frequencies. Vibrations above 200 Hz appear to contribute modestly to the discrimination, and those towards the highest frequencies up to 800 Hz, even less so.

In Fig. 3, two specific colonies’ instantaneous alarm is computed across the entirety of their respective datasets, showcasing the outcome of the algorithm for a colony that swarmed (Fig. 3a) and another that did not (Fig. 3b). For the extensive collection of alarm plots pertaining to all colonies see Figs. S1–S14 (non-swarming) and Figs. S15–S32 (swarming) of the supplementary material.

In Fig. 3a, we see a representative example that shows that our discriminant functions (Fig. 2) are successful in discriminating between the change of preparation status between a colony that is aiming to swarm and the comparatively steady-state of the preparation status of a colony that is not aiming to swarm (Fig. 3b). Affected by the spectral variations induced by foraging activities, the alarm is not triggered for data leading up to the primary swarm that were collected between 9:00 AM and 4:00 PM. The spectra at these peak foraging times must therefore resemble that determined by our software as a non-swarming spectrum. Similarly, the hourly alarm is also triggered during foraging hours later in the season, as shown by occasional purple pixels in July and August. By averaging the night time alarm between 12:00 AM and 5:00 AM, giving a daily assessment of a colony’s preparation to swarm, we show that our algorithm, for this specific colony, is able to determine that the colony was going to swarm one month before the exit of the primary swarm. We also show that for a specific, representative colony that did not swarm, the alarm was never triggered (Fig. 3b) either on an hourly or daily basis. Alarms displayed across Supplementary Figs. S15 to S22 show that the placement of the accelerometer across the frames of the broodbox is nearly irrelevant for the prediction of swarming, with all eight frames monitored across the season for this specific colony successfully producing a swarming alarm several days before the swarming event. This suggests that the accelerometers embedded in the honeycomb pick up the entire colony’s overall buzzing, irrespective of the frame on which they were affixed.

For swarming colonies (Supplementary Figs. S15–S32), based on the mean (red line) of the night time alarm, the alarm was successfully triggered in 15 out of 18 datasets before the exit of the primary swarm. Moreover, the alarm was triggered on average twenty-two days before the exit of the primary swarm. For the non-swarming colonies, no false positives are found across the collection of datasets (Supplementary Figs. S1–S14), based on the mean night-time alarm, giving a 91% success rate in the software’s ability to discriminate between our swarming and non-swarming colonies. Note that the black sections seen within the hourly alarm plots across the 2014 French colonies between the 17^th^ and 24^th^ March, the 17^th^ and 24^th^ August and the 14^th^ and 27^th^ November are the result of missing data in the datasets, due to power cuts. This caused the mean alarm to drop below the threshold, as their values are strictly zero; those values were not considered as false detections.

Despite all our efforts, false positives are still triggered occasionally on an hourly basis, and this becomes exacerbated when the time duration of the season under scrutiny is extended to the rest of the summer. We also explored whether our algorithm was lacking the required sensitivity, or whether the data were genuinely lacking specificity to the preparation of swarming. To this end, a simple cross-correlation exercise was used to compare the similarity between individual swarming and non-swarming spectra which make up each training database. This revealed that night-time instantaneous spectra taken from a colony preparing to swarm can, in some instances, be virtually identical to that taken outside of the swarming season, or from a colony not preparing to swarm (Supplementary Fig. S33). This lack of specificity of these particular data therefore suggests that there is no need to seek for more advanced algorithms to discriminate the required categories. We therefore moved on to the exploration of a new feature, one accounting for the recent history of the evolution of the spectra, effectively removing the flaw of finding identical spectra in the PTS and NS clusters.

### Strategy 2 – Discrimination between Spectral Evolutions

**Figure 6:**
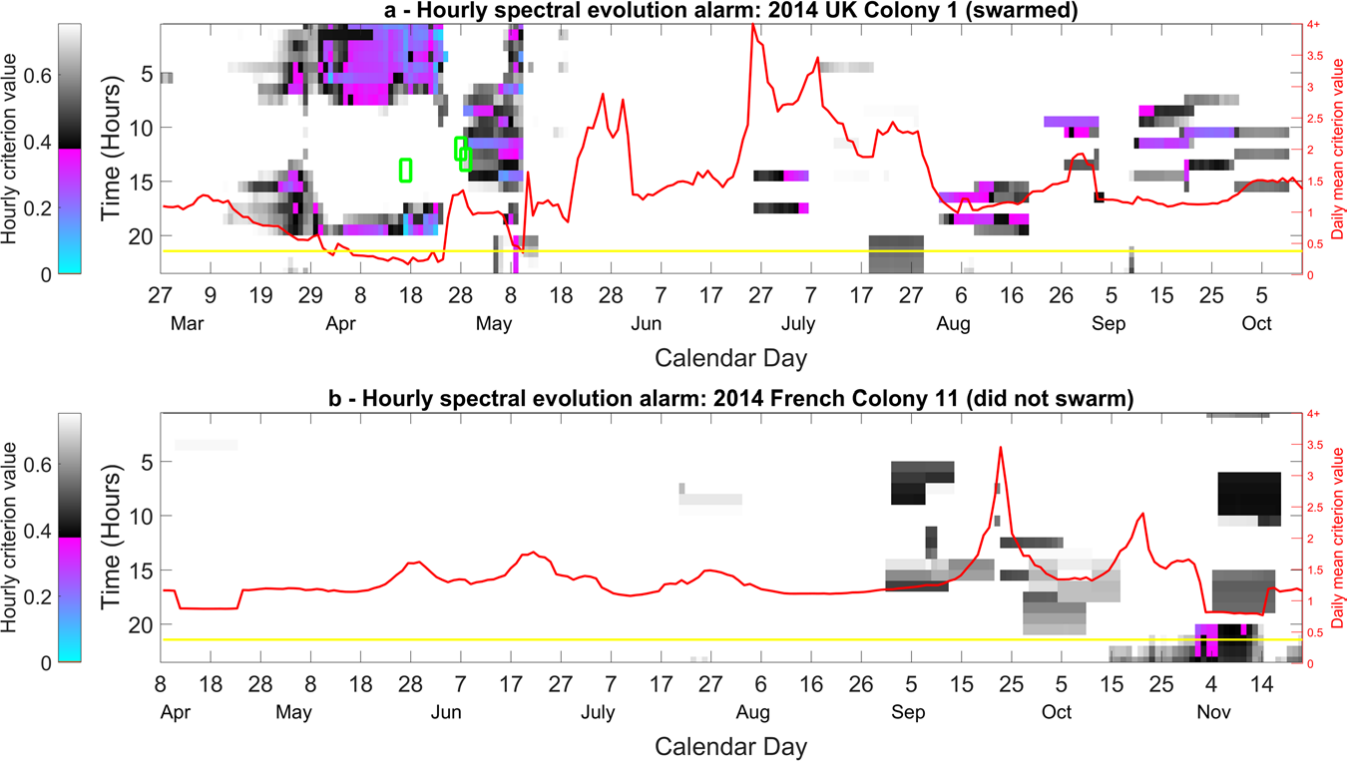
Swarming alarm based on the 10-day evolution of spectra for a colony that (**a**) swarmed, situated at Nottingham Trent University and (**b**) did not swarm, situated at INRA, France. Each colony was monitored across the active season in 2014. The colour coding is identical to that of Fig. 3. Green rectangles display the timings of three swarms that occurred within this dataset. Super imposed is another graph with the y-axis (at right) being the daily mean criterion value computed from data between midnight and 5:00 AM; the yellow line displays the alarm threshold. This figure was created using the MATLAB (R2018b) software (uk.mathworks.com).

Parametric optimisation explored a collection of 126 swarming and 1512 non-swarming 3DFT (three dimensional Fourier transform) spectra, and used an optimised training database based on 20 swarming and 21 non-swarming spectra, obtained between midnight and 5:00 AM, incorporating the previous 10 days of history leading up to the primary swarm, and cropped over a bandwidth of 25 to 800 Hz, with 40 percent of the PCA scores being projected onto three DF scores (see Fig. 4 for more information). This resulted in the best performance from the software (as displayed in Fig. 6). These parameters yielded a 1.92% classification error between the 1638 (126 swarming and 1512 non-swarming) data points used in the clustering exercise. The DF spectra in Fig. 5 exhibit features that are very different from those seen in Strategy 1, with most variation observed below 700 Hz.

**Figure 7:**
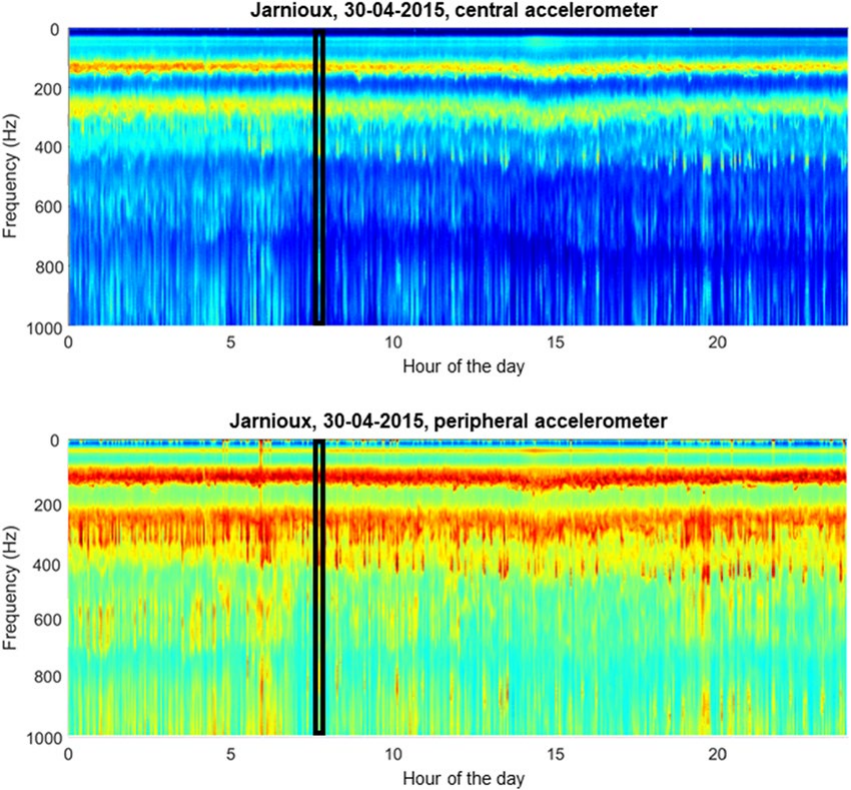
Continuous raw accelerometer data, averaged over three minute intervals, for frequencies between 0 and 1000 Hz, recorded from a single colony in Jarnioux, France, on 30^th^ April 2015. Data are shown for the accelerometers situated at the centre (**a**) and at the periphery (**b**) of the frame. Data shown were recorded on the 30 April 2015. Colour coding represents logarithmic spectral amplitude from low (blue) to high (red) in arbitrary units. Highlighted in the black rectangle is the short sample of data that is represented in Fig. 8. This figure was created using the MATLAB (R2018b) software (uk.mathworks.com).

**Figure 8:**
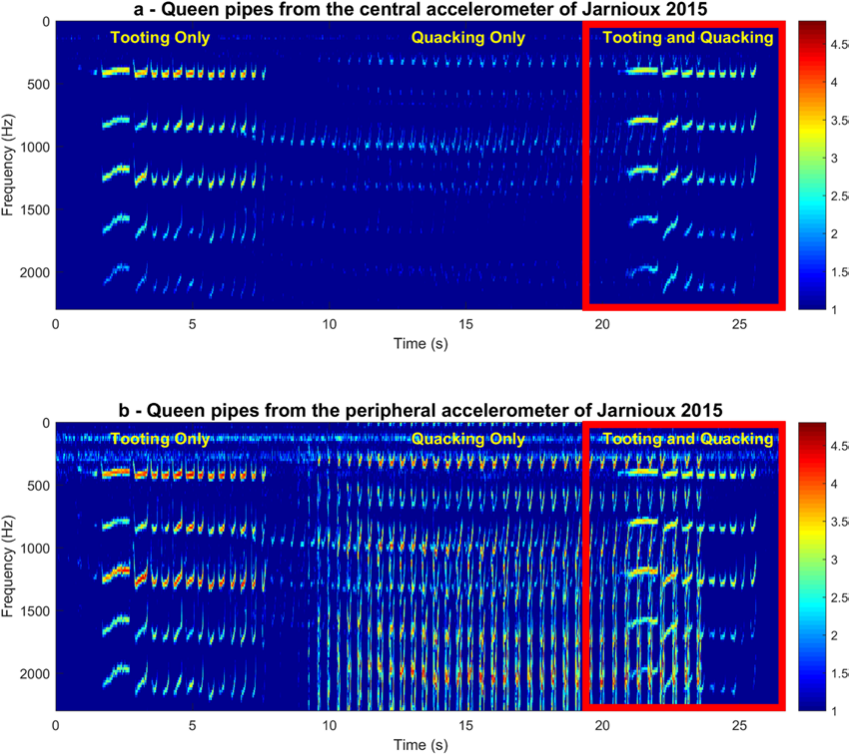
Spectrogram of raw spectra containing the toots and quacks found in the section highlighted in Fig. 7. Data are shown for the accelerometers situated at the centre (Top, **a**) and at the periphery (Bottom, **b**) of the frame. Pixel intensity represents logarithmic spectral amplitude from low (blue) to high (red) in arbitrary units. Highlighted in the red rectangle is the small sample of data further analysed in Fig. 9. This figure was created using the MATLAB (R2018b) software (uk.mathworks.com).

Swarming alarm values based on the ten-day evolution of spectra for two individual colonies are computed across the entirety of their respective datasets, showcasing a colony that did (Fig. 6a) and did not (Fig. 6b) swarm. For the extensive collection of alarm plots pertaining to all non-swarming and swarming colonies see Supplementary Figs. S34–S47 (non-swarming) and Supplementary Figs. S48–S64 (swarming).

In Fig. 6, we demonstrate that our discriminant functions incorporating the 10-day spectral evolution (Fig. 5) are successful in generating mean criterion values that discriminate between a colony that is and is not preparing to swarm, with the alarm triggered and sustained 16 days before the exit of the primary swarm in the example. In addition, the daily mean midnight to 5:00 AM criterion value did not trigger the alarm after the exit of the final afterswarm (except perhaps temporarily on the 10^th^ May). Due to the variation induced by foraging activities, as seen for the instantaneous method in Fig. 3, the alarm is not triggered for spectral data from between the hours of 6:00 AM and 4:00 PM leading up to the primary swarm. The spectra at this prominent foraging time must therefore, again, more closely resemble those determined by our software as non-swarming spectra. In addition, the hourly alarm is sporadically triggered during foraging hours later in the season, e.g. 9^th^ August and 15^th^ September, at a similar rate to that of Fig. 3. These false positives can be effectively dealt with by averaging the resulting alarm from midnight to 5:00 AM. Alternatively, these alarms may be meaningful, as in north-eastern North America scientists have shown that in addition to a major period of swarming in May/June, a minor period of swarming also exists in September.

We also show a representative example of a colony that did not swarm (Fig. 6b) where the alarm is never triggered, based on the alarm averaged between midnight and 5:00 AM. Note that the hourly alarm for the non-swarming colony is only ‘mildly’ triggered a few times around 8:00 PM between the 1^st^ and 4^th^ November, as alarm values are very close to that of the threshold, performing similarly to that of the instantaneous spectrum alarm in Fig. 3b.

For the non-swarming colonies (Supplementary Figs. S34 to S47), based on the mean midnight to 5:00 AM alarm, only two colonies produce false positives within the midnight to 5:00 AM average alarm, across the entire collection of datasets. These false alarms occurred within the 2014 French colony 5 dataset between the 28^th^ April and 3^rd^ May 2014 (Supplementary Fig. S37), and the 2014 French colony 22 between the 10^th^ and 20^th^ July (Supplementary Fig. S47). For swarming colonies (Supplementary Figs. S48–S64), based on the mean of the night time alarm, in 13 out of 18 datasets, the alarm was successfully triggered before the exit of the primary swarm, but it was not successful in predicting swarms for the 2014 French colonies 10 (Supplementary Fig. S58) and 13 (Supplementary Fig. S59), as well as 2015 French colonies 15 (Supplementary Fig. S63), and 19 (Supplementary Fig. S64). This gives an overall success rate of 80% in the software’s ability to discriminate between our swarming and non-swarming colonies, with an average of 10 ± 2 days (n = 11) warning (calculated from those colonies that were correctly classified as swarming colonies), when using the mean midnight to 5:00 AM mean criterion values to assess the swarming preparation of honeybees. As with the previous method using the instantaneous spectra, Fig. 3a and Supplementary Figs. S48 to S54 reveal that 8/8 frames monitored across the UK colony 1 hive successfully triggered a swarming alarm several days preceding the swarming event, suggesting again that the lateral placement of the accelerometer does not affect the prediction of swarming using this technology.

### Comparison between the two strategies

In Table 1, we see a direct quantitative comparison between the performances of the two strategies for the prediction of swarming using averaged vibrational spectra. With 22 days compared to 10.1 days, we can see that strategy 1, using instantaneous spectra, provides a much longer swarm warning than strategy 2, using the spectral evolution. When using the average daily criterion values, only 3 colonies were wrongly classified from the collection of 32 when using strategy 1, compared to the 7 misclassified by strategy 2. ‘Raw alarm false positives’ refers to the number of colonies that have hourly alarm triggers (criterion value falls below established swarming threshold) when considered not in a state of preparation to swarm, i.e. after the last queen pipe or from a non-swarming colony. We see here that strategy 1 and 2 performed equally, with all colonies exhibiting at least some false positives on an hourly level. ‘Raw alarm false negatives’ refers to the number of colonies where, on an hourly basis, no alarm trigger occurred when the colony was considered to be in a state of preparation to swarm, i.e. in the two weeks leading up to the primary swarm. In this respect, strategy 1 again outperformed strategy two, with only two swarming colonies lacking an hourly alarm trigger compared to the three of strategy 2. It is clear from this comparison that strategy 1 was much more effective than strategy 2 and that the calculation of the early morning mean daily criterion value is an essential tool for averaging out hourly false positives.

**Table 1:** Quantitative comparison of the performance evidence for each swarm prediction strategy discussed in this paper.

Quantitative criterion	Strategy 1 – Instantaneous Spectra		Strategy 2 – Spectral Evolution	
Averaged predictive warning	22 days		10.1 days	
Averaged alarm errors (number of colonies which produced false negatives (swarming) or false positive (non-swarming) when using the mean criterion value)	*Swarming Colonies*	*Non-Swarming Colonies*	*Swarming Colonies*	*Non-Swarming Colonies*
	3/18	0/14	5/18	2/14
Raw alarm false positives (number of colonies with hourly alarm triggers when considered not in a state intending to swarm)	*Swarming Colonies*	*Non-Swarming Colonies*	*Swarming Colonies*	*Non-Swarming Colonies*
	18/18	14/14	18/18	14/14
Raw alarm false negatives (number of colonies with no hourly alarm triggers when considered in a state of intending to swarm)	*Swarming Colonies*	*Non-Swarming Colonies*	*Swarming Colonies*	*Non-Swarming Colonies*
	2/18	N/A	3/18	N/A

## Queen Pipes

### Toot and quack signals characterisation

**Figure 9:**
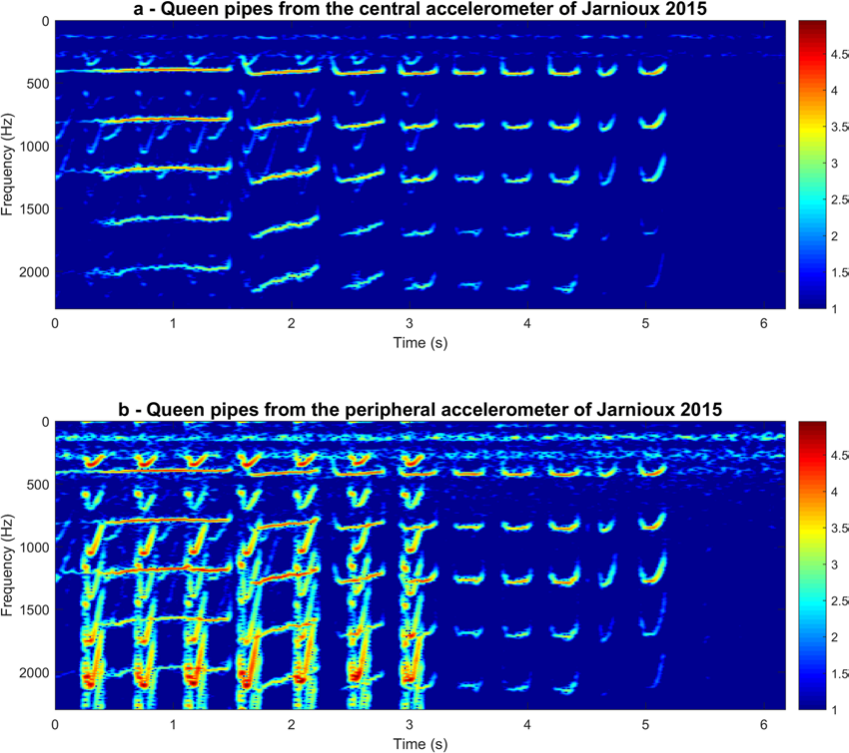
Spectrogram of raw spectra containing the toots and quacks found in the section highlighted in Fig. 8. Data shown are for the accelerometers situated at the centre (Top, **a**) and at the periphery (Bottom, **b**) of the frame. Colour coding represents logarithmic spectral amplitude from low (blue) to high (red) in arbitrary units. This figure was created using the MATLAB (R2018b) software (uk.mathworks.com).

We now proceed to carefully examine the queen pipes residing within our measurements. As raw audio recordings were also available for the Jarnioux 2015 dataset, we combined them with the daily spectrograms of the 3-minute averaged spectra to draw further conclusions regarding the occurrence of queen toots and quacks in each of our datasets (Supplementary Figs. S65 to 72). In Fig. 7, a daily spectrogram of vibrations can be seen for the Jarnioux dataset of 30^th^ April 2015, 9 days after the primary swarm, for both the central (Fig. 7a) and peripheral (Fig. 7b) accelerometers. Within the data of this particular day, we see the general buzzing of the bees that occurs at 125 Hz and 250 Hz, its upper harmonic. In addition to these two highly reliable continuous traces, two sets of intermittent peaks can be seen between 300 and 350 Hz that diverge at around 5:00 AM, with one set exceeding 400 Hz whilst the other remains at 300 Hz. A small section of these data from 8:00 AM, located within the black rectangle, was extracted, analysed separately and displayed in Fig. 8. Through critical listening and visual inspection, we ascertained that the higher frequency band at around 400 Hz specifically pertains to queen toots and the lower frequency vibrations at around 300 Hz correspond to the quacking of encapsulated gynes (Fig. 8). The audio sample of these virgin queen vibration signals presented in Fig. 8 can be found in Audio S1. When we further home onto these (Fig. 8), we can see the differences in the spectral properties of the two types of queen pipes. In the example showcased in Figs. 8 and 9, queen tooting consists of a 1-second-long narrowband pipe followed by a train of multiple (eight in this case) 0.2-second-long pulses of the same frequency. In contrast, the quacking by cell-captive mature gynes held in their cells by worker bees is comprised of a series of usually many 0.2-second-long pulses that occur around 300 Hz. These are much more severely frequency-modulated and resemble the U-shaped figure of the train of pulses that make up the last few pulses of the queen toot, only at a lower frequency. In particular to the signal showcased here, seen in Fig. 9, tooting occurs in intermittent 5-second-long signals, whereas quacking persists uninterrupted for over 20 seconds. It can also be seen that the toots and quacks are much more prominent on the spectrograms pertaining to the peripheral accelerometer compared to that of the central one. This is due to the location upon the comb in which each type of signal was produced. During the swarming process, queen cells are usually built at the periphery of the frame (as opposed to the centre during emergency queen rearing), and queens tend to toot nearby (Grooters, 1987). As a result, both piping signals have a much greater magnitude on the peripheral accelerometer as compared to the central one (Figs. 7, 8 and 9).

### Chronology of toots and quacks in disturbed and undisturbed colonies

**Figure 10:**
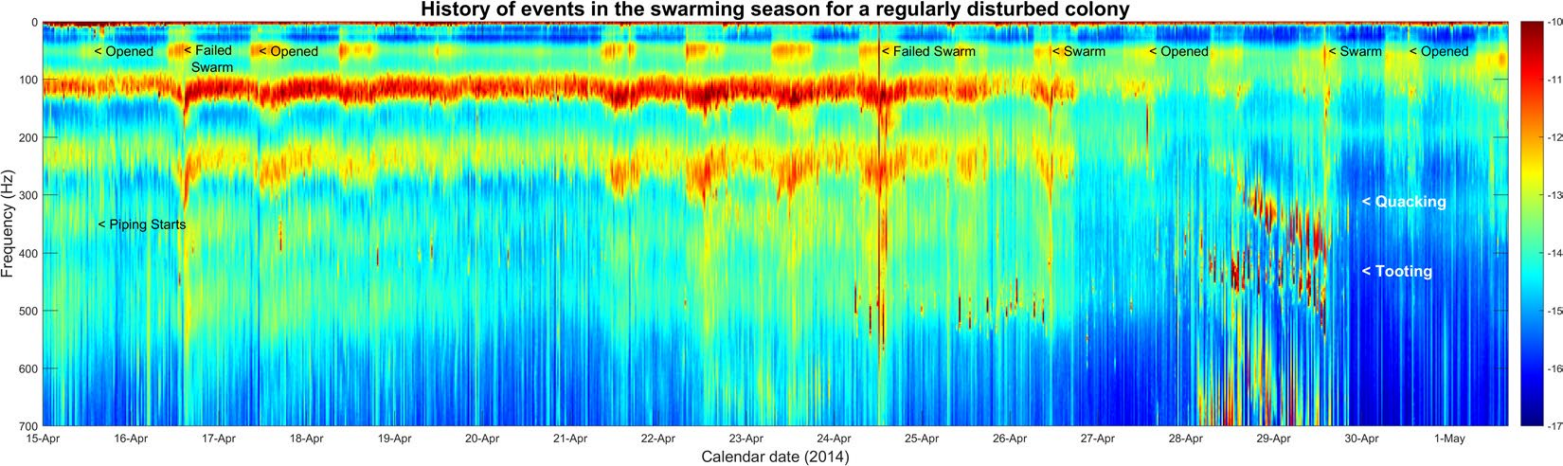
Spectrogram image of 3-minute averaged spectra extracted from the 2014 French colony 14 dataset, showcasing the occurrences of queen piping recorded within a colony that contained a clipped queen and was disturbed throughout its swarming activities between 15^th^ April and 2^nd^ May 2014. Midnight of each day begins at each date label on the horizontal axis. Pixel intensity represents logarithmic spectral amplitude from low (blue) to high (red) in arbitrary units. This figure was created using the MATLAB (R2018b) software (uk.mathworks.com).

**Figure 11:**
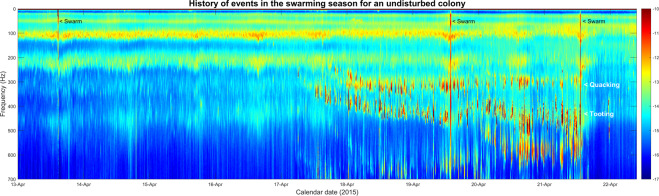
Spectrogram image of 3-minute averaged spectra extracted from the 2015 French colony 22 dataset, representing the occurrences of queen piping recorded within a colony with an original queen that was left unclipped and was left undisturbed throughout its swarming activities between 13^th^ and 22^nd^ April 2015. Midnight of each day begins at each date label on the horizontal axis. Pixel intensity represents logarithmic spectral amplitude from low (blue) to high (red) in arbitrary units. This figure was created using the MATLAB (R2018b) software (uk.mathworks.com).

In Fig. 10, we see data from a colony (French Colony 14, 2014) that originally had a clipped queen, and whose hive was opened several times during the swarming season. Faint, scarce and irregular virgin queen tooting started on the 15^th^ April 2014 two hours after a large colony level disturbance caused by planned hive maintenance that took place. It suggests that the event most probably resulted in the early release of an encapsulated gyne. It is difficult to see the tooting on the spectrogram in Fig. 10 due to the low density of its occurrence, however in Supplementary Fig. S73, we depict the hour pertaining to the beginning of tooting on the 15th April. This shows a high amplitude broadband spectrum, synonymous to that obtained when a hive is opened, that occurred at midday on the 15^th^ April. Piping then started two hours later, as highlighted in Supplementary Fig. S73. A swarm was attempted and failed the next day on the 16^th^ April, likely due to the clipped wings of the original queen. The swarm is identified by a high amplitude broadband vibrational peak, especially at ultra-low frequencies around 60 Hz, twice lower than that of the 125 Hz buzzing, which occurs at the time of the lift-off, with a significant drop in signal amplitude thereafter (see Supplementary Fig. S74). When a swarm fails and returns to the hive, a large peak occurs at a fundamental frequency of around 150 Hz, just after the swarming event, as the bees re-enter which causes a buzzing vibrational amplitude increase rather than the expected decrease (Supplementary Fig. S74). The hive was opened again on the 17^th^ April. Following this, the 400 Hz virgin queen tooting can be seen with greater intensity than previously observed. However, the toots appear much scarcer and sporadically distributed, (24 hours between consecutive toots) than would normally be expected from a newly emerged tooting queen (see the 17^th^ April onwards in Fig. 11). This is perhaps another consequence of a queen having been released early due to human intervention. Another failed swarming attempt occurred on the 24^th^ April. A successful swarm finally ensued on the 26^th^ April, which resulted in the large decrease of the buzzing vibrational signal and the abrupt cessation of queen tooting, strongly suggesting that a virgin queen left with the swarm. This phenomenon is also seen for 2014 French colonies 1, in Supplementary Figs. S65 and S6, in Supplementary Fig. S66, where tooting can be observed before the exit of the first successful swarm. As seen in Fig. 10, the hive was opened for a third time on 27^th^ April, resulting in new queen tooting a few hours later. Quacking then ensued after six hours. Tooting and quacking both increased in frequency from 400 Hz to 500 Hz, and from 300 Hz to 400 Hz, respectively. Both tooting and quacking then terminated upon the exit of the final afterswarm, suggesting that an emerged virgin queen left with the swarm and an encapsulated gyne emerged and stayed within the colony.

In Fig. 11, the data for the swarming events of French Colony 22 in the 2015 season are shown. The original queen was unclipped, and this particular colony was left undisturbed throughout its swarming activities. As expected, no piping can be seen before the primary swarm that issued at 2:00 PM on the 13^th^ April. Frequent queen tooting started four days later on the 17^th^ April and increased in signal frequency from 300 Hz to 450 Hz in the two days leading up to the first afterswarm. Six hours after the start of tooting, quacking can be observed to increase from 280 Hz up to 350 Hz, which then holds stable until cessation at the second afterswarm. In all cases (Supplementary Figs. S65–S72), tooting always preceded quacking. After the first after swarm, there was a three-hour hiatus in queen tooting for this particular colony, whilst the quacking persists, and there appears to be a reduction in the occurrence of quacking, a phenomenon also seen across the other datasets (Supplementary Figs. S65–S72). This suggests that the emerged virgin queen left with the swarm and a previously encapsulated gyne emerged. Both tooting and quacking then persisted until the final swarm after which, like all other colonies (Supplementary Figs. S65–S72), they are no longer apparent in the dataset.

## Discussion

In this work, we disclose a non-invasive method for the prediction and the monitoring of the swarming process within honeybee colonies. As such, two machine learning algorithms, along with their respective discriminant functions (Fig. 2 for instantaneous spectra alarm and Fig. 6 for the spectral evolution alarm) that act as generic signatures for swarming and non-swarming classification, are presented for the prediction of swarming. These discriminant functions are based on the overall three-minute averaged vibrational spectra recorded using accelerometers placed in the comb among brood cells at the heart of honeybee hives. Averaging the accelerometer data removes the need to create exceptionally large databases containing the raw data pertaining to each hive, as seen for Ramsey *et al*.^15,16^, and provides data relevant to the overall colony buzzing states, irrespective of the frame of the hive in which the sensor is placed.

The primary swarm is remarkably difficult to predict from accelerometer measurements since it is not associated with any queen piping. On the contrary, secondary swarms always appear to take place after many hours of queen pipes that are straightforward to detect as soon as they appear in the colony. The main challenge that this work endeavoured to overcome is the prediction of primary swarm events when no obvious swarming-specific features exist within the averaged spectrum. As such, both of our algorithms can successfully discriminate between swarming and non-swarming colonies with a high degree of accuracy, with the analysis based on instantaneous spectra performing with a classification error of 6.3% within the swarming season, and the spectral evolution analysis performing with an error of 1.91% without any restriction on the season under scrutiny. In this paper, we also disclose an original analysis of the accelerometer trace pertaining to, and an extensive collection of, queen toots and quacks, which allowed for us to disclose new features of queen piping in relation to other colony events, over the entire swarming season for each hive, both for inspected and non-inspected colonies.

Provided that the user restricts the application of the instantaneous spectra method for swarming prediction to data from the swarming season alone, this algorithm works remarkably well. Even whilst necessitating one hour of recording each day between 3:00 and 4:00 AM, the instantaneous spectra only failed to predict three out of eighteen primary swarms and did not exhibit a single false positive from the non-swarming colonies. Further investigation into the false positives exhibited outside of the swarming season revealed that instances exist throughout the swarming and non-swarming training databases where the night-time spectra taken from colonies that are considered in a state of preparing to swarm are almost identical to spectra extracted from outside the confines of swarming (see the example in Supplementary Fig. S33). This demonstrates the poor specificity of instantaneous spectra to swarming prediction and encouraged us to explore an alternative history-carrying feature in order to lift the obvious lack of specificity of instantaneous spectra. Between the three discriminant functions generated as part of the training phase of our discrimination exercise, we also show that there is little information above 700 Hz useful to the prediction of swarming. In fact, the largest variation, and therefore the most important information, is shown to occur around 20 Hz, which matches remarkably well with the frequency of the DVAV signal^16^. In previous work^16^, we have shown that our accelerometer technology is very effective for the detection of DVAV signals, for which the generally accepted function is that of “prepare for greater activity”^29^ and that enhancements in this signal can be seen prior to the exit of the primary swarm. From the stronger emphasis that our discriminant spectra demonstrate around 20 Hz compared with frequencies above 100 Hz, we can deduce that the general buzzing of the bees alone does not give important or reliable information pertaining to a colony’s intent to swarm. This is in direct contrast to Woods^18^, Ferrari *et al*.^19^ and Vancata^20^ who have suggested that sound spectra above 200 Hz are modulated by honeybee activity with high specificity in the days leading up to the primary swarm with high specificity. The 20 Hz feature we reveal to be imperative to the prediction of the swarming process using spectral information also undermines previous authors work using microphones^18–21^ because DVAV signals are a non-audible signal and microphones are incapable of detecting frequencies as low as 20 Hz. An accelerometer, on the other hand, has no limitations at such low frequencies, adding further substantiation to the use of accelerometers for monitoring honeybee hives.

The improved accuracy of the discrimination procedure based on the ten-day spectral evolution can easily be seen in the hourly alarm plots, with far fewer false positives occurring on an hourly basis after the end of the swarming season for the ten-day spectral evolution than that based on the instantaneous spectra. One drawback of the spectral evolution method, however, is that at least ten days of data are required before the alarm value can be computed and assessed, and this requires a much larger storage capacity. Additionally, the software to run the analysis is much more computationally demanding when compared to analyses based on the instantaneous spectra. The alarm for each method is significantly further improved if it is averaged over the hours of midnight and 5:00 AM, for the instantaneous spectra, and 8:00 PM and 1:00 AM for the alarm based on the spectral evolution. This provided a daily averaged alarm that maintained true alarms and removed sporadic false positives (See Fig. 3). From this, it is seen that the instantaneous spectra method outperformed that of the spectral evolution in terms of the number of days prior to swarming that the alarm was triggered and the low number of false positives within the non-swarming data. This indicates that, in one way or another, information regarding the ‘history’ of the spectra is required. In the context of a commercial product aimed at aiding the beekeeper, it could be argued that false positives that arise using either algorithm after the end of the swarming season for colonies that did swarm are irrelevant, because a swarming detector would be unnecessary after the departure of the primary swarm or after the swarming season has come to an end (after July). Nevertheless, late (e.g. September) swarming and absconding outside the typical swarming season are phenomena known to occasionally take place^30^. In future work, it would be highly interesting, should such data be available, to see whether our alarm would successfully detect fall swarming events. The lack of specificity of the features found in the instantaneous spectra causes numerous false alarms during busy foraging times of the day. The use of early morning data is best for colony status assessments, as successful foraging afternoons, can lead to enhanced vibrational activities until midnight.

Both strategies could perhaps be further improved through the inclusion of additional grouping clusters at the training phase from colonies that exhibit data that account either for honeycomb density due to differing brood levels or comb contents (e.g. honey and pollen), or that have some feature (e.g. ‘varroa infested’ or ‘not varroa infested’) that makes their vibrational spectra differ from others. These algorithms can also be easily developed to determine the likelihood (percentage) that a colony is going to swarm, based on the alarm values calculated from the ratio of distances to each centroid, of the percentage likelihood that a colony is going to swarm. This would allow the beekeepers to prioritise the hives that require the most urgent inspection.

### Queen pipes

In addition to our algorithm for the prediction of primary swarms, our daily spectrograms give new insight into the physical features of queen piping following the primary swarm. As such, this work allowed for comparison of the waveform properties of toots and quacks across the entire swarming season obtained by our accelerometer measurements to that of the laser vibrometer (a method unable to provide the long-term measurements that accelerometers can) used by Michelsen *et al*.^10^ (described in the introduction). Our results substantiate theirs. For tooting, we show that there is a frequency increase from 350 Hz to 500 Hz that occurs gradually as the first gyne to emerge ages, reaching a steady state just before she exits with an afterswarm. The analysis of quacking also showed a similar phenomenon consisting of a rise in frequency from 200 Hz up to 350 Hz over a few successive days. It was also found, through critical listening and close examination of spectra that pertained to the raw accelerometer audio of the Jarnioux 2015 dataset, that queen tooting consists of a 1–2 second pipe followed by a train of 0.2 second pulses of the same frequency, as described by Wenner^9^ and Michelsen^10^. In contrast, quacking by gynes confined in queen cells consists of long series of 0.2 second pulses that occur around 350 Hz. These results also correspond very well with the physical properties described by Kirchner^11^. We also found that queen quacks exhibit much broader spectral variation around the fundamental frequency than the much narrower band found in queen toots (Fig. 7). Owing to these spectral differences, queen toots and quacks can be discriminated by eye in most instances, within the 3-minute average spectra (e.g. see the 5:00 AM divergence of 350–500 Hz toots from steady 350 Hz quacking in Fig. 7), allowing for the visual detection of their natural occurrences within the continuous vibrational datasets available to us in this study.

The Jarnioux 2015 colony (Supplementary Fig. S72) and the 2015 colony 22 (Fig. 11) were left undisturbed with non-clipped queens during their swarming processes. As can be seen in the data and corroborated by the raw accelerometer audio, queen tooting started four to seven days following the exit of the primary swarm, with quacking ensuing after the initiation of tooting. This duet of tooting and quacking persisted until the exit of an afterswarm when tooting ceased for a few hours before being logged again, strongly suggesting that the next gyne had been released from her cell. This sequence of events repeats itself until the exit of the final afterswarm, at which time there is not a single gyne remaining in her cell. In some instances, queen tooting continues for several days after the exit of the final swarm. As such, our ethological approach provides strong evidence in support of the theory of the queen piping being a colony-level communication to aid the worker population in the co-ordination of the release of queens by conveying information about how many queens are free and encapsulated to avoid competition between them (as described in Winston^6^). In the undisturbed colonies, if it were not for the worker bees actively keeping the encapsulated gynes captive, then two or more queens would be observed tooting contemporaneously (this is never heard in the raw accelerometer recording). The timings of our logged toots and quacks are very much in line with the work of Grooters^12^, further substantiating their conclusions that queen tooting was an important mechanism for the prevention of rival queen emergence. Our results also further substantiate the well-accepted idea that only mobile queens produce tooting signals, since the issue of secondary swarms consistently resulted in the abrupt cessation of tooting, even if only for a few hours.

An exciting further experiment would be to drive artificially generated toot signals into the hive of a colony immediately following the exit of the first afterswarm to reveal if workers bees extend the duration in which gynes remain captive. In a study by Simpson and Greenwood^31^ artificial 650 Hz pulsed vibrations were generated to mimic queen pipes in hives of colonies containing a single mobile gyne and concluded that this encouraged them to swarm. The conditions under which this experiment was conducted does not allow for us to explore the effect that this artificial piping could have had on worker bees maintaining encapsulated gynes, and possibly suggests that tooting sensed by a mobile gyne, indicating the presence of a second one, in the absence of quacks, is more likely to result in her exit with a swarm than in the absence of any pipe.

In colonies that were disturbed several times during their swarming procedures or in colonies containing queens with clipped wings, an entirely different set of phenomena were observed. Hive 1 (Supplementary Fig. S65), 10 (Supplementary Fig. S67) and 13 (Supplementary Fig. S68) from France in 2014, for example, had several failed swarming attempts, which can be seen as a large high-magnitude buzzing trace that appears after the swarm as the bees unexpectedly re-entered their hive, as opposed to the expected and otherwise observed loss of vibrational signal that would normally result from a large amount of bees exiting the hive. This transpired because the queen with clipped wings was unable to fly with the swarm. These colonies were also opened several times during the early stages of the swarming process. This led to queen pipes occurring before the first swarm that ceased upon the successful exit of the swarm. This suggests two possibilities 1) there were two (or more) emerged queens (the old queen and a newly emerged virgin gyne) present in the hive at the same time, due to inspection disturbance causing the early release of a virgin queen, and the primary swarm left with the additional virgin queen, or 2) it was the original mated queen that was piping during this time, who then left with the primary swarm, indicating that mated queens, one or more years of age can also pipe^32^. However, the ages of the queens were not known. In colonies with a queen that cannot fly, a range of phenomena can take place (see footnote).

Footnote for this page only: personal communication from Professor Gard Otis, who observed 4 colonies that swarmed with virgin queens without having issued a prime swarm with the mated queen. He referred to those swarms as “afterswarms” because they had virgin queens. In two instances the mated queens were unable to fly with the swarm, and they disappeared once a virgin queen emerged from her cell; those colonies proceeded to issue afterswarms with virgin queens. However, in one case, the mated queen remained in the hive as it swarmed with her present colony; two afterswarms with virgin queens issued, after which the original mated queen once again became the egg-laying queen of the colony. She later did leave with a prime swarm in the subsequent swarming cycle (see Otis^33^).

Our method and results strongly suggest that fundamentally important colony disturbance can be brought by beekeeping activities during the rearing of virgin queens, which is a time when more frequent beekeeping inspections are recommended. In colonies left undisturbed with non-clipped queens, our methods have preserved what can be considered the natural pre- and post-swarming behaviour of honeybees. We have demonstrated that such natural behaviour can be lost through invasive inspections, resulting in the early release of a virgin queen, so that more than one mobile queen is present within the hive. This is not a natural situation for the colony, which otherwise delivers an orderly release of gynes in relation to swarming events^6^. This also supports the suggestion that tooting acts to aid in the prevention of the simultaneous emergence of rival queens, as during physical inspections by beekeepers, tooting would be interrupted on each frame as it is removed for inspection, allowing the opportunity for the encapsulated gyne to make the final cuts to the cell cap and emerge from its cell. It may also be suggested that the swarming event itself may stimulate the emergence of encapsulated gynes. For those colonies containing clipped queens, failed swarms ensued and in the cases of 2014 French colony 6 (Supplementary Fig. S66), 10 (Supplementary Fig. S67) and 12 (Fig. 11), the early tooting that occurs could also be the consequence of a queen emerging due the swarming attempts, and not that of opening the hive, causing the early release of an encapsulated gyne. It could therefore be considered that clipping queens causes havoc within the natural swarming procedure of a colony. If made available to beekeepers, our swarming prediction alarm would remove the necessity of queen clipping altogether, as well as minimise the need for other repeated invasive procedures, and allow for colonies to develop more naturally.

## Conclusions

To conclude, what we present here is a significant advancement in the detection of pre-swarming cues with the use of averaged vibro-acoustic information to non-invasively monitor the swarming activities within honeybee colonies, without the need for invasive visual observations. Through novel analytical techniques, we developed two methods for the prediction of swarming in honeybee colonies. Each provides a generic signature that enables automatic assessments of a colony’s intent to swarm, further substantiating the usefulness of accelerometer technology for the *in-situ* non-invasive monitoring of honeybee colony status. The timings of the toot and quack signals that we have recorded, relative to those of swarms, strongly support the idea that tooting and quacking enable the colony to regulate afterswarming and ensure that the parental colony is always left with at least one mature gyne after a swarm has issued.

## Methods

### Continuous recording of vibrational data

**Figure 12:**
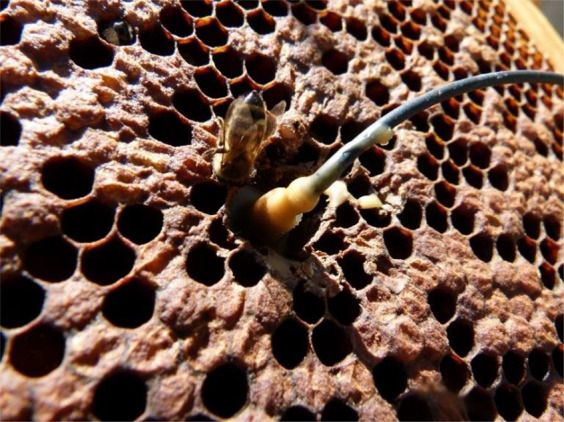
A Brüel and Kjaer piezoelectric Type 4508 accelerometer installed in the centre of a frame from a bee hive.

Accelerometers were embedded in the wax at the centre of the central frames of twenty-five honey bee hives containing various subspecies of *Apis mellifera*. All accelerometers were placed among bee brood^14^. Twenty-two hives at the Institut National de la Recherche Agronomique (INRA) were monitored using high sensitivity (100 mV/g) Brüel and Kjær Type 4508 piezoelectric accelerometers. One colony at an apiary in Jarnioux, France and two at the Nottingham Trent University (NTU) Clifton Campus, UK were monitored using ultra-high sensitivity (1000 mV/g) accelerometers of the same type. These accelerometers are of exacting specification with a quoted frequency-dependent sensitivity variation of about 2% over this range, an operational temperature range of −54 to 121 °C and a temperature coefficient of sensitivity of +0.006% per 1 °C. The Brüel and Kjær software PULSE was used to process the signal digitised by the B&K Type 3053-B-120 conditioner, and collect these measurements continuously, in the form of power spectra averaged every 3 minutes, with a bandwidth of 5500 Hz and a frequency resolution of 3 Hz. Over 30 million individual frequency resolved data points were logged and analysed. The apiary at INRA was comprised of one Langstroth and nineteen Dadant hives that were established two meters apart in a row approximately 40 meters in length in Avignon, France, in March 2014. Additional accelerometers were also placed in the wax at the centre of seven other frames within one of the NTU hives. All accelerometers were oriented so that their sensing direction was perpendicular to the plane of the comb to obtain the greatest sensitivity. To install the accelerometers, a cavity (1 cm^3^) was made at the centre of the focal comb, an accelerometer was then placed inside, and a small amount of molten wax was applied to aid in securing its position and prevent any direct exposure of the metal components to the bees (Fig. 12).

Hives were continuously monitored for vibrations throughout the 2014 and 2015 seasons, apart from short electrical power cuts after which the data loggers resumed automatically once power was restored. Each day, the previous day’s spectrogram of vibration was generated to continuously monitor the progress of each of the twenty-four hives throughout the entirety of their time under observation. An excellent array of swarming events (a total of eleven primary swarms and nineteen afterswarms) were captured in the data across both the 2014 and 2015 seasons.

### Swarming prediction using averaged vibrational spectra

#### Training Phase

##### Strategy 1 – Discrimination between Instantaneous Spectra

A specific swarming feature that worked over an entire season was not found during the development of this algorithm. However, a specific signature was obtained when restricting the data to the swarming season only.

Datasets from colonies that swarmed were selected through visual inspection of the daily spectrogram of vibration (see Fig. 7 and Supplementary Fig. S74). To compile the average spectra to be used in the *Preparing to Swarm* (PTS) training database, data for the thirteen days prior to the primary swarm were selected from each hive that swarmed. For *the Non-Swarming* (NS) dataset, spectra gathered after the last queen pipe recorded in the swarming colonies were further labelled as ‘not preparing to swarm’. Spectra were also compiled and added to the training database from the entire swarming season (April – July) for those colonies that did not swarm. As honeybees (*Apis mellifera* spp.) remain in their hives overnight, only data logged from midnight to 06:00 were included to ensure they were not influenced by foraging activity, which upon inspection was found to yield highly variable and unpredictable spectral information. Data were averaged to give a mean spectrum for each hour of recording and were separately uploaded into the software. The data were then normalised and reshaped to create a linear array which could later be fed into the PCA/DFA algorithm (for further information see e.g. Bisele, *et al*.^28^, Bencsik, *et al*.^14^, Ramsey, *et al*.^15,16^) to identify spectral information unique to each category.

##### Strategy 2 – Discrimination between Spectral Evolutions.

We also attempted a second, much more computationally intensive, algorithm that included information about the spectral history in a procedure we term “spectral evolution”. The basic concept behind this algorithm was to use the temporal evolution of the spectrum of vibration that takes place over the days leading up to a primary swarm, rather than relying on a single instantaneous spectrum. To compute the spectral evolution, the FFT is calculated for the time course of the magnitude of all the uploaded spectral frequencies for each day between midnight and 5:00 AM, yielding 2DFT images. The FFT is then further calculated over each pixel of the series of 2DFTs found in the preceding days, the number of which was explored during parametric optimisation, resulting in a 3D spectroscopy exercise. The power spectra information in both the 2DFT and 3DFT revealed changes in the history of the spectra, without keeping the absolute timings of the changes. This prevents the risk of training the algorithm to absolute timings. The 3DFTs are then reshaped and compared to a series of discrimination curves identified through rigorous training of a Discriminant Function Analysis (DFA) algorithm (see below) for categorisation of a colony as being either in a swarming or non-swarming state.

To create these DF curves, two training databases were first established, made up of spectral information from colonies that either did or did not prepare to swarm. The PTS database was comprised of data containing information (on the ten-day spectral evolution) of the six days leading up to issuance of the primary swarm of eighteen different datasets (11 colonies including one swarm made available from 8 independent accelerometer measurements in separate frames) from the UK and French colonies in 2014 and 2015. The NS database was made up of the 3D spectra from across the active season of fifteen colonies that did not swarm and the 3D spectra after the final afterswarm of the ten colonies that did swarm. To compute the spectral evolution from a time period not affected by the variation induced by foraging, the three-minute power spectra were uploaded each day between the hours of midnight and 5:00 AM.

##### Principal Component and Discriminant Function Analysis.

For both strategies, the training databases were reshaped to create a linear array, normalised with respect to their mean and, only in the case of the instantaneous spectra algorithm, log transformed to help scale-up minute differences in spectra. The databases then underwent reduction using Principal Component Analysis (PCA) to create a series of PCA scores that best describe the data deviations.

For each method, the categories were carefully selected within the training database and their PCA scores were calculated. By using a triplet of cross correlation products with three discriminant functions identified by the DFA algorithm (previously made available online, Bencsik, *et al*.^15^), three discriminant function coordinates, or ‘DF scores’, could then be calculated for each measurement. The centroids for each group were then calculated. From these, a “threshold value” for swarming detection could be determined for the ratio of distances from the point in DF space to each centroid, considered the “alarm” value, between the PTS and NS centroids that dictated whether a measurement was classified as being in a state of ‘preparing to swarm’ or ‘not preparing to swarm’. The optimum threshold value was that which allowed the highest number of successful classifications. To reduce the possibility of over-fitting, and to find the most generic discrimination functions, an iteration procedure similar to that of Bisele, *et al*.^28^ was also implemented. Every pulse combination was explored within the training database, removing all spectra except for a reduced collection that best represents the overall information pertaining to each category, allowing for clustering with the lowest overall misclassification. However, spectra removed from the training database that were not included within the computer training stage were still included in the clustering process to find their DF space co-ordinates and contribute to the calculation of the overall classification error.

##### Parametric Optimisation.

The numerical search that we have performed was entirely self-contained within the MATLAB high level programming language, from data upload to the swarming signature extraction. The signal processing comprises numerous steps, all of which can be tailored by the user to refine the outcome of the search. This allowed us to run optimisation loops, in which a specific parameter along the processing stages was systematically changed, until the extracted swarming signature provided the best outcome, i.e. lowest overall misclassification. This numerical exercise, which we call ‘parametric optimisation’, works given that we have a scalar quantity that, at the end of a specific processing chain, provides an estimate of the performance or of our swarming detection. This scalar quantity was determined by calculating the proportion of incorrectly classified points; it defined what we call the percentage error.

To achieve optimisation, a number of parameters were explored to obtain the lowest percentage error of discrimination between points pertaining to each group. Within the optimisation loop, parameters that were changed independently from each other were: scaling (logarithm vs. linear spectral amplitude); the lower and upper limits of the frequency bandwidth of the spectra contributing to the search; the number of days of data leading up to the primary swarm included in the PTS training dataset; the specific hour over each day from which the data were extracted; the number of PCA scores contributing to the DFA search (too low will remove important information, too high will bring in ‘noise’ and overfitting artefacts); the specific spectra fed to the PCA algorithm to get the PCA scores (this will dictate the relevance of the first PC scores, as discussed earlier); and finally, the number of DF scores (two or three in our case) onto which the selected PCA scores are collapsed. To dismiss the variation that sometimes arises due to the influence of the wind (see Supplementary Figs. S75 and S76), frequencies below 18 Hz were systematically excluded. In the case of the strategy making use of the spectral evolution, the number of days contributing to the spectral evolution was also explored.

We tested the algorithms on the swarming season datasets for all colonies. For each dataset, spectra were averaged over each hour and then categorised as belonging either to a PTS or NS state, using a triplet of cross correlation products with the two discriminant functions identified by the DFA algorithm. The threshold value acted as the “alarm” which is triggered if a specific spectrum was deemed to be close enough to the swarming centroid. The alarm value was plotted for each hour of the day throughout the swarming season. The exact time when a primary swarm left the hive within each dataset was then also displayed. This allowed to visually assess how many days prior to that time the alarm was triggered and to also identify any false positives or negatives.

### Continuous in-hive monitoring of queen pipes

In 2015, an additional hive was equipped with a 1000 mv/g Brüel and Kjaer piezoelectric Type 4508 accelerometer for the exploration of raw accelerometer data. The data pertaining to this hive, named the Jarnioux 2015 dataset, are fully described and partially available as part of previous publications by Ramsey, *et al*.^15,16^. These data were intentionally transformed into daily spectrograms of individual spectra averaged over three minutes with parameters strictly identical to those used by the PULSE software that stored the other datasets. In particular, the frequency resolution was set to 3 Hz whilst the bandwidth was set to 5 kHz.

The spectral signature unique to each queen pipe was identified through critically listening to the raw audio of the Jarnioux 2015 dataset (e.g. S1 Audio) and cross referencing this to the three-minute average spectra at the associated time points. This allowed for visual discrimination between toots and quacks within vibrational datasets comprising of three-minute averaged spectra. The occurrences of toots and quacks were then examined visually within the daily images and presented alongside each other and other key colony-level events.

## Supplementary information

Supplementary Figures.

Audio S1 - Toots and Quacks
