# Supplementary material for: The prediction of swarming in honeybee colonies using vibrational spectra
Source: Sci Rep. 2020 Jun 16;10:9798. doi: 10.1038/s41598-020-66115-5 (PMC7298004; doi:10.1038/s41598-020-66115-5)
Supplement: Supplementary file 1 — Supplementary Figures. [file 41598_2020_66115_MOESM1_ESM.docx]

Title: The prediction of swarming in honeybee colonies using vibrational spectra.

Authors

Michael Ramsey^­1^, Martin Bencsik^1^*, Michael Newton^1^, Maritza Reyes^2^, Maryline Pioz^2^, Didier Crauser^2^, Noa Simon Delso^3^, and Yves Le Conte^2^.

^1^ Nottingham Trent University, School of Science and Technology, Clifton Lane, Nottingham, NG11 8NS, United Kingdom

^2^ INRAE, UR 406 Abeilles et Environnement, Domaine Saint-Paul, 84914, Avignon, France

^3^ Centre Apicole de Recherche et d'Information, CARI, 4, Place Croix du Sud, B-1348, Louvain-La-Neuve, Belgium

* E-mail: [martin.bencsik@ntu.ac.uk](mailto:martin.bencsik@ntu.ac.uk)

Supplementary Material

*Michael.Ramsey@HLSUK.LOCAL*

**Instantaneous spectra for colonies that did not swarm, showing the average daily criterion values for detection of swarming**

In this section, Fig S1 – S14 show the alarm based on instantaneous spectra for various colonies monitored across the 2014 active season. The plots have been grouped together because they represent colonies that **did not swarm**.


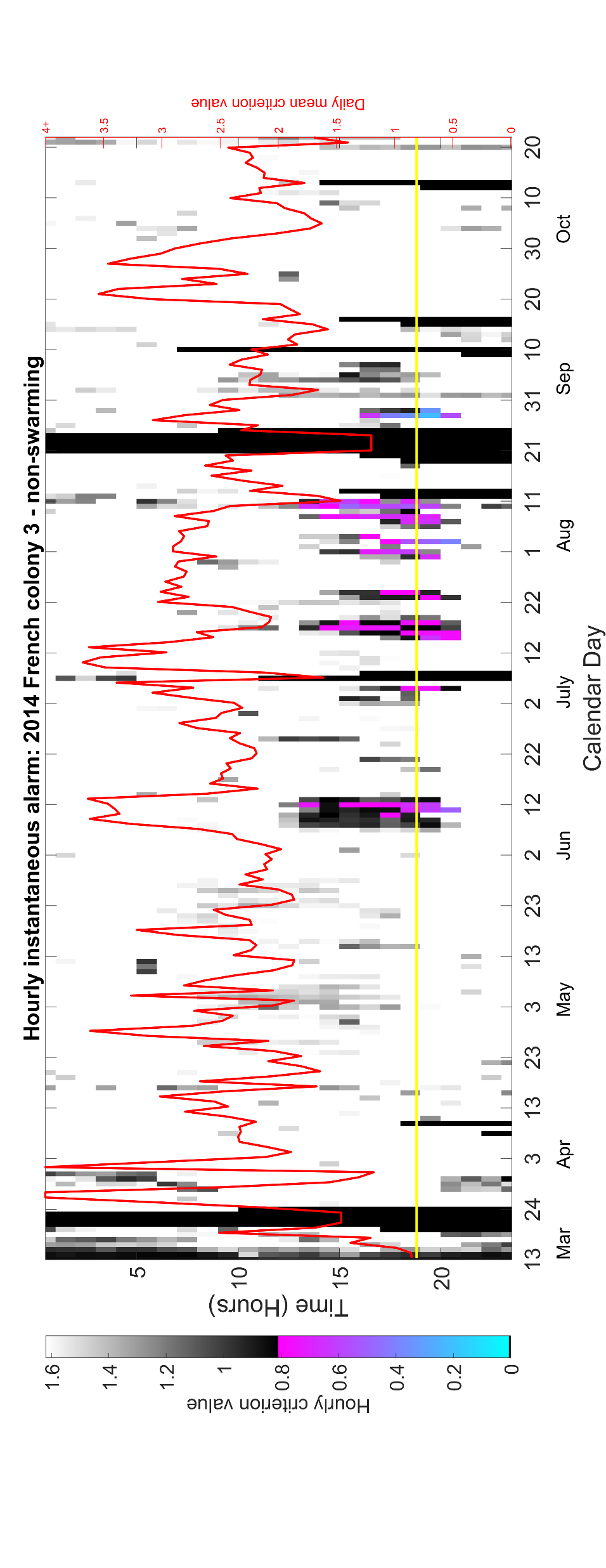

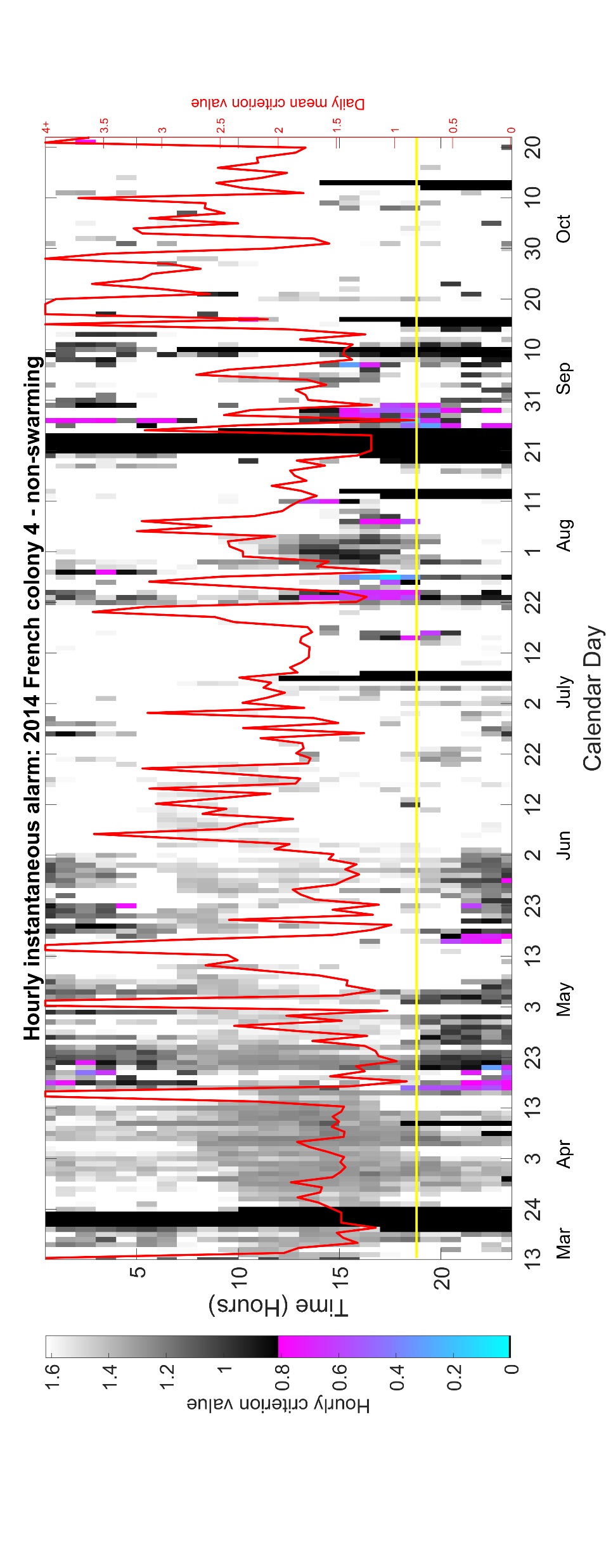

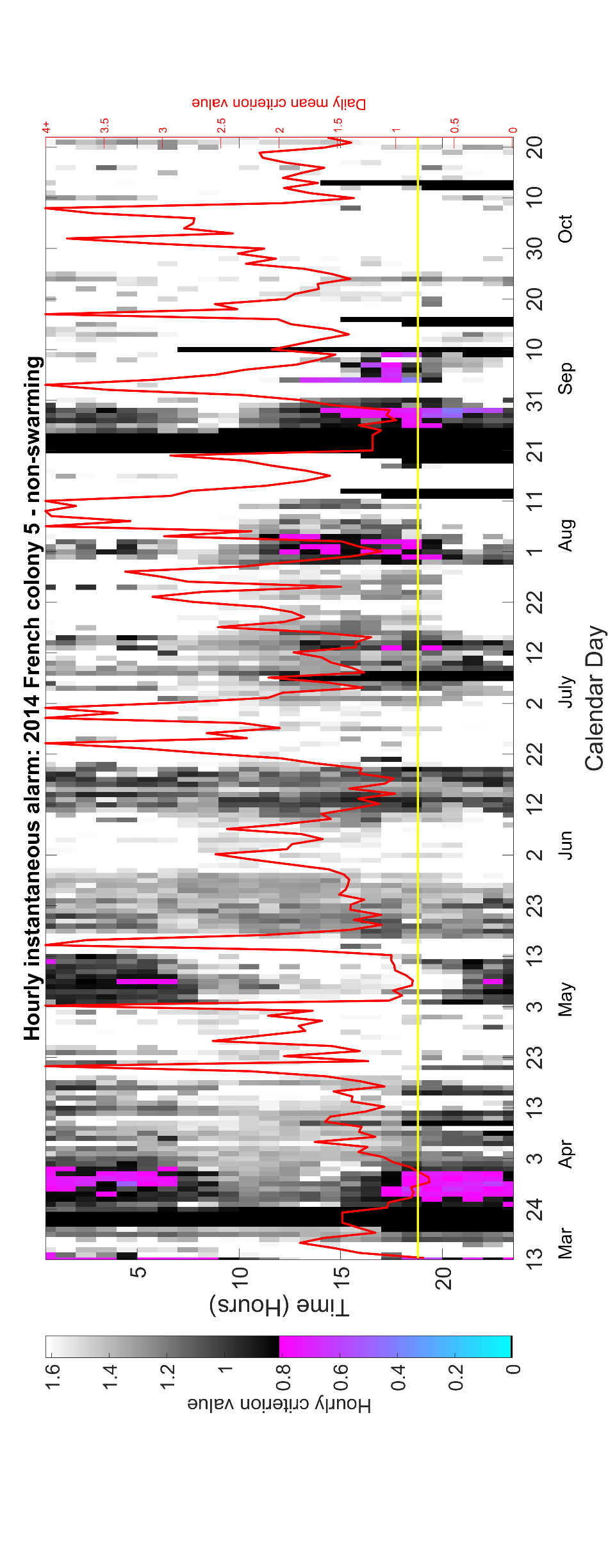

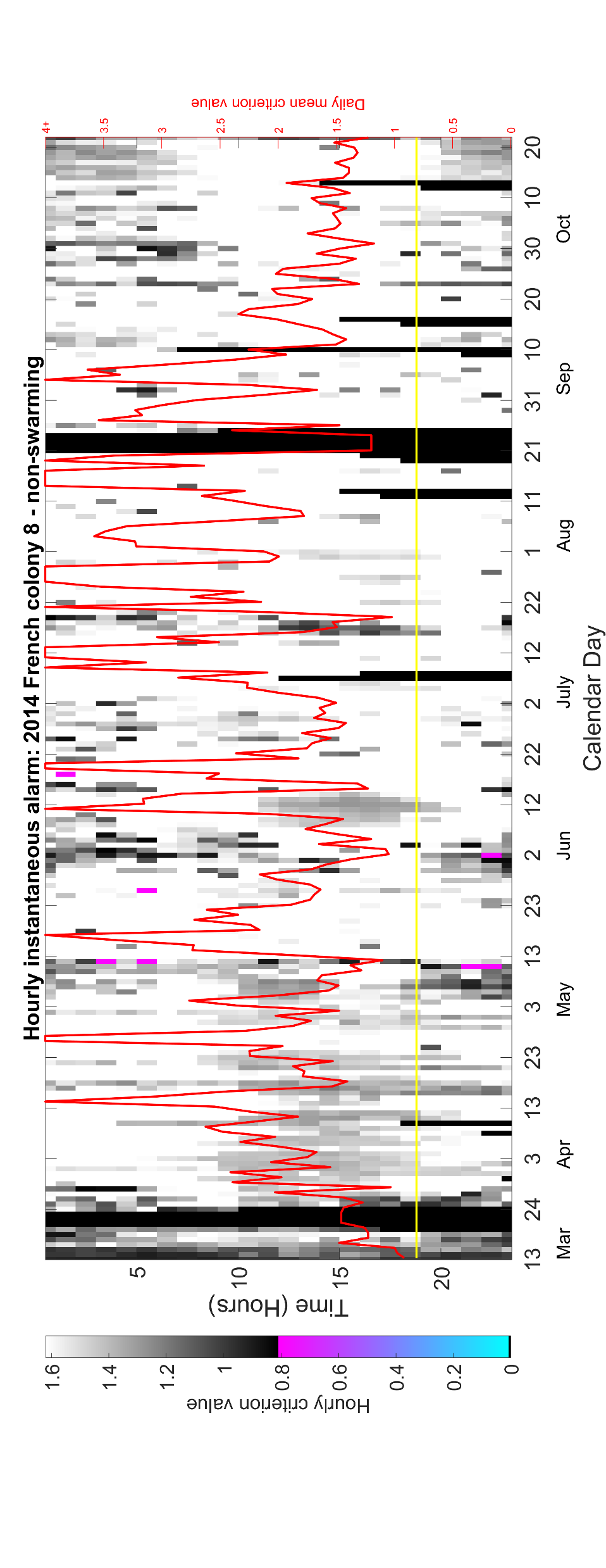

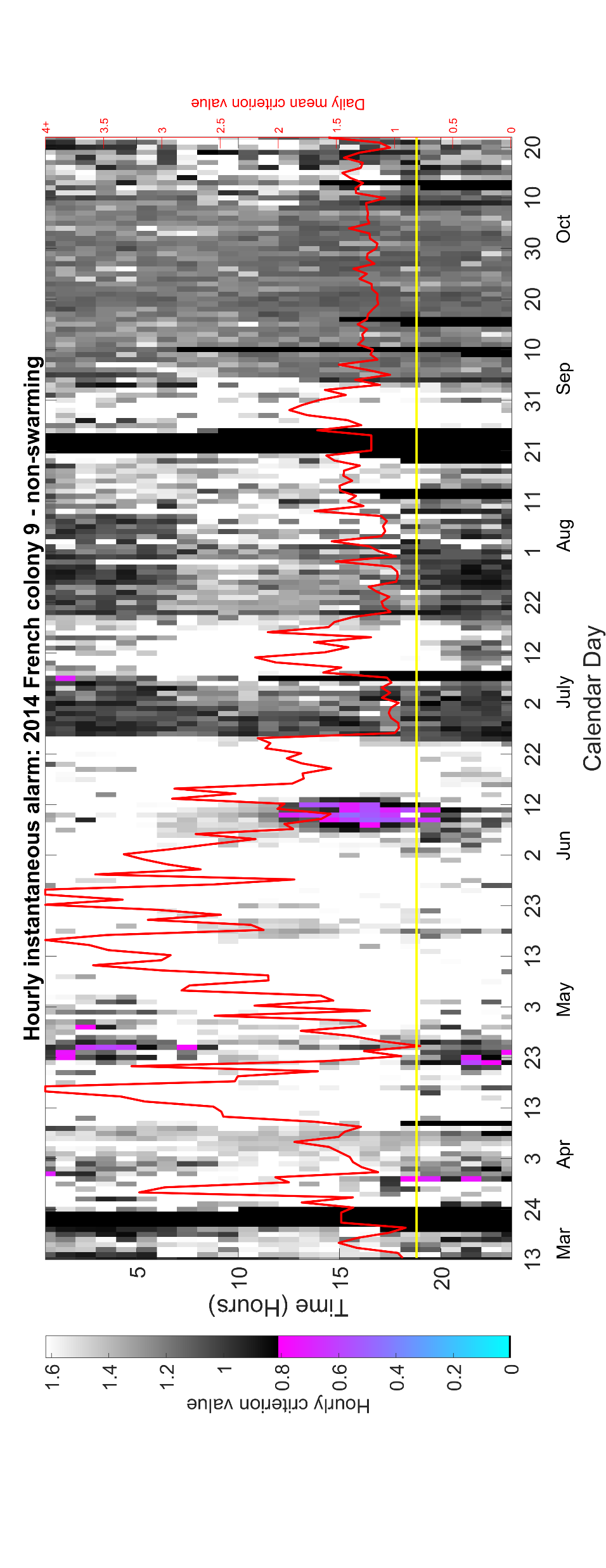

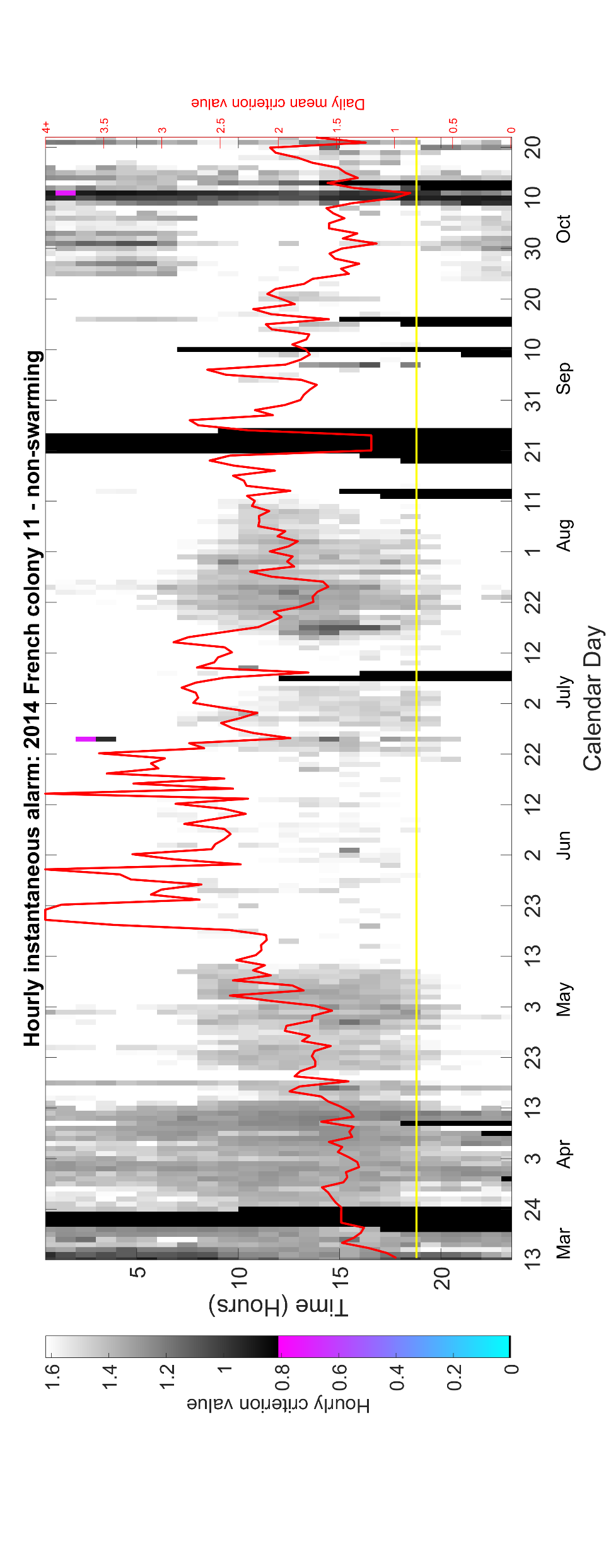

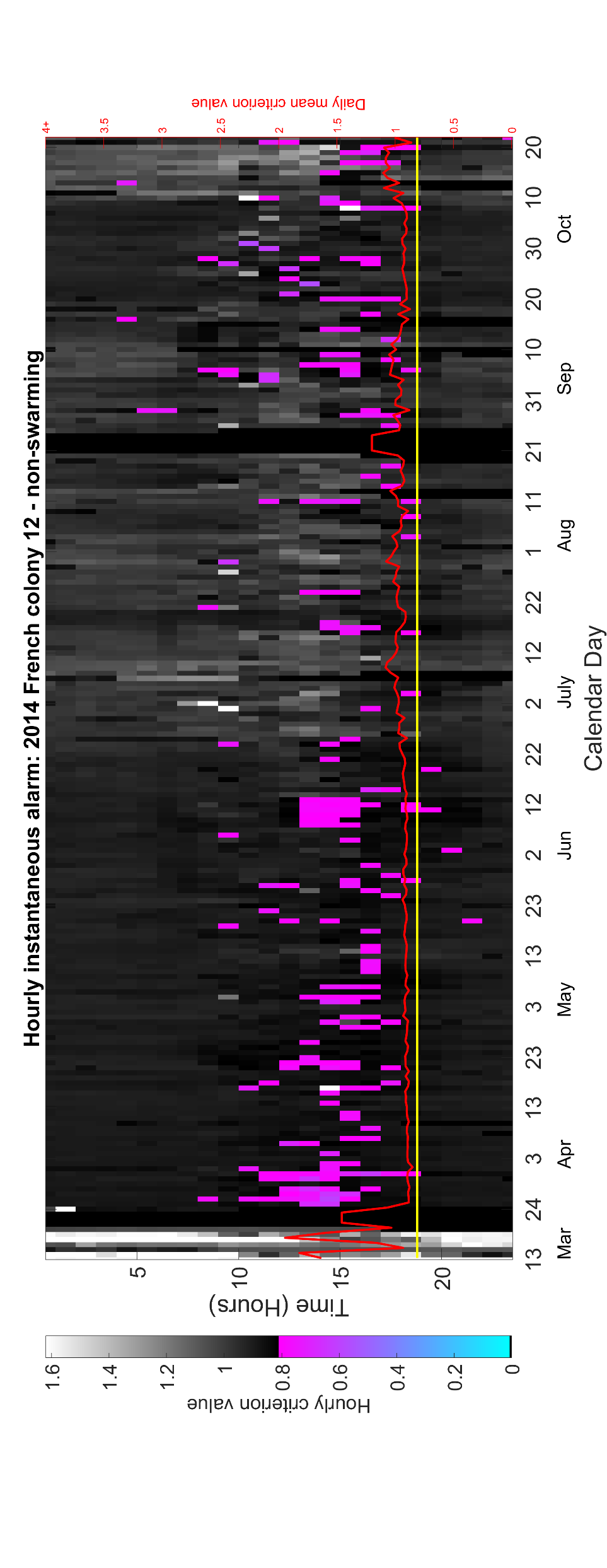


*Fig S1:* ***Non-Swarming Colony.***

***Instantaneous alarm for 2014 French colony 3*** *shown from the 13^th^ March until the 20^th^ October 2014. The colour coding has been split. Greyscale and coloured pixel intensity denote alarm values above (non-swarming state) and below (swarming state) the threshold, from pink to blue as the criterion approaches the swarming centroid. Superimposed is another set of axes showing the average of the previous night’s alarm taken between midnight and 5am, with the yellow line displaying the alarm threshold.*

*Fig S2:* ***Non-Swarming Colony.***

***Instantaneous alarm for 2014 French colony 4*** *shown from the 13^th^ March until the 20^th^ October 2014. All figure parameters are identical to that described in Fig S1.*

*Fig S3:* ***Non-Swarming Colony.***

***Instantaneous alarm for 2014 French colony 5*** *shown from the 13^th^ March until the 20^th^ October 2014. All figure parameters are identical to that described in Fig S1.*

*Fig S4:* ***Non-Swarming Colony.***

***Instantaneous alarm for 2014 French colony 8*** *shown from the 13^th^ March until the 20^th^ October 2014. All figure parameters are identical to that described in Fig S1.*

*Fig S5:* ***Non-Swarming Colony.***

***Instantaneous alarm for 2014 French colony 9*** *shown from the 13^th^ March until the 20^th^ October 2014. All figure parameters are identical to that described in Fig S1.*

*Fig S5:* ***Non-Swarming Colony.***

***Instantaneous alarm for 2014 French colony 5*** *which was monitored from the 13^th^ March until the recording ceased on the 20^th^ October 2014. All figure parameters are identical to that described in Fig S3.*

*Fig S6:* ***Non-Swarming Colony.***

***Instantaneous alarm for 2014 French colony 11*** *shown from the 13^th^ March until the 20^th^ October 2014. All figure parameters are identical to that described in Fig S1.*

*Fig S7:* ***Non-Swarming Colony.***

***Instantaneous alarm for 2014 French colony 12*** *shown from the 13^th^ March until the 20^th^ October 2014. All figure parameters are identical to that described in Fig S1.*

The data for colony 12 (Fig S7) appear very close to the threshold throughout the entire monitoring period. The data for this colony were examined and were probably biased, due to hardware anomaly that remains unidentified. The data show very little variation across the season, unlike other colonies.
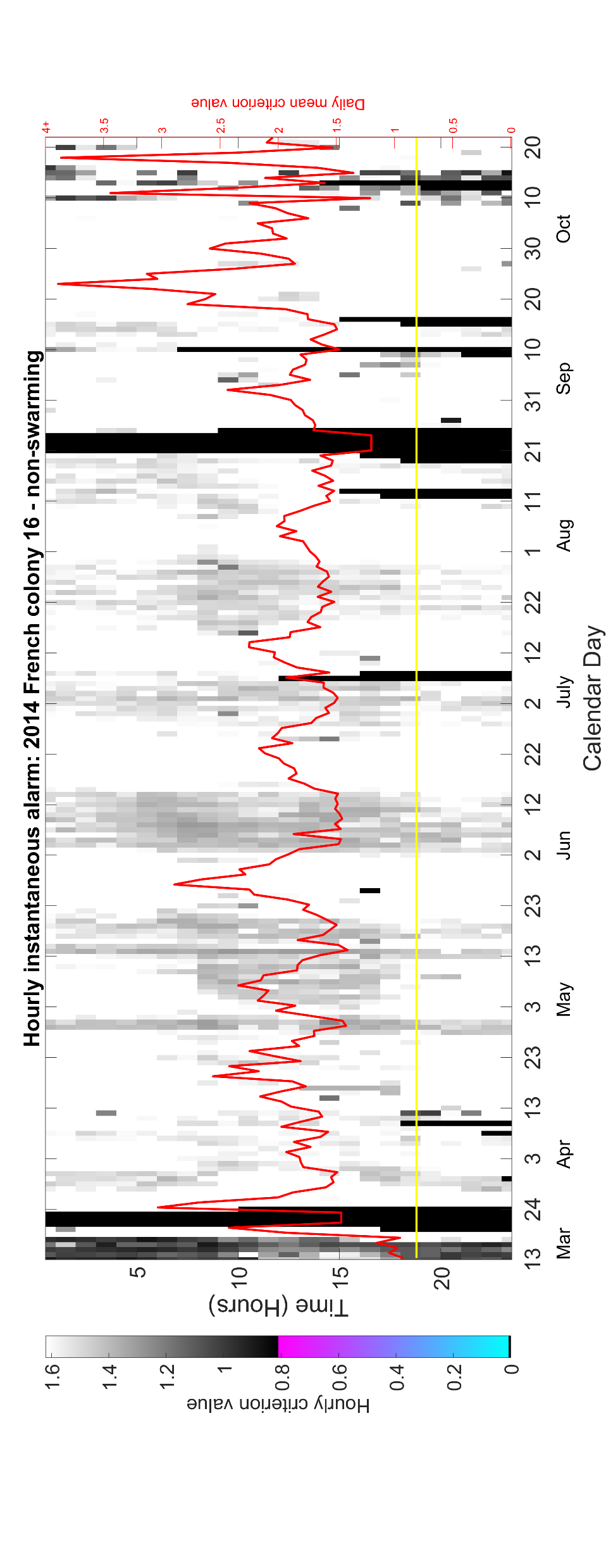

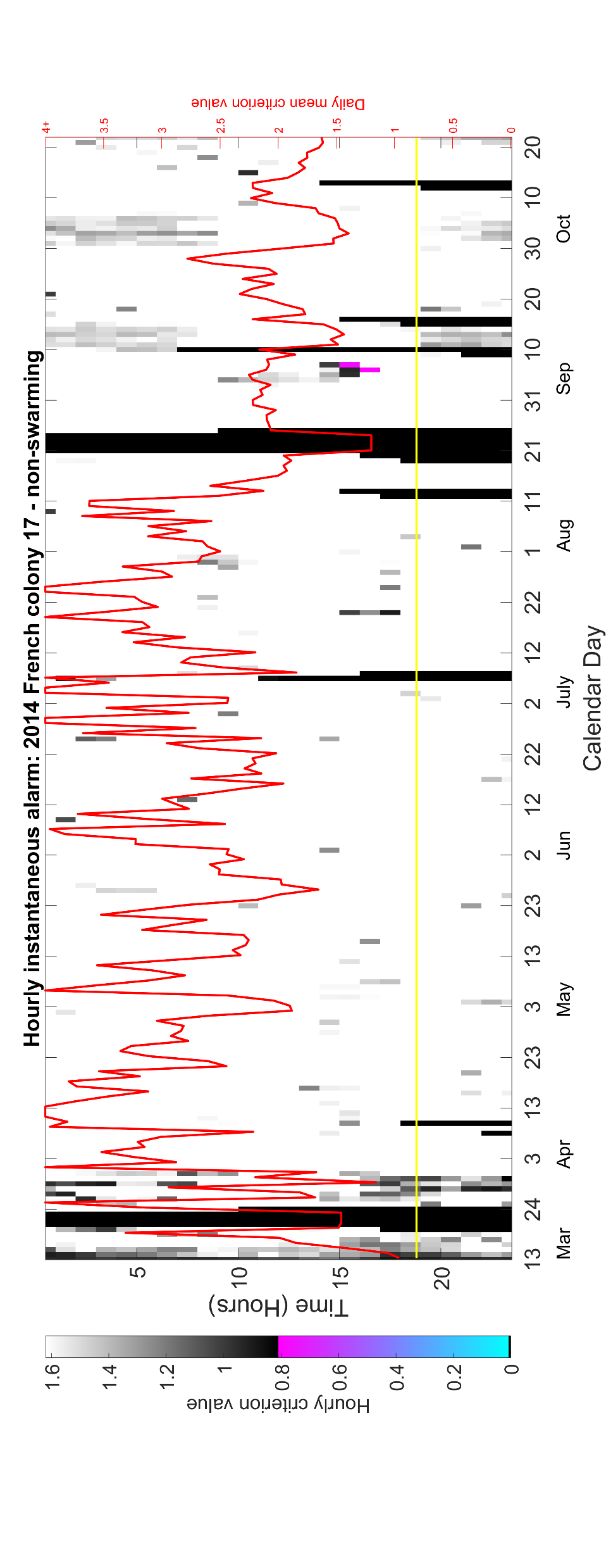

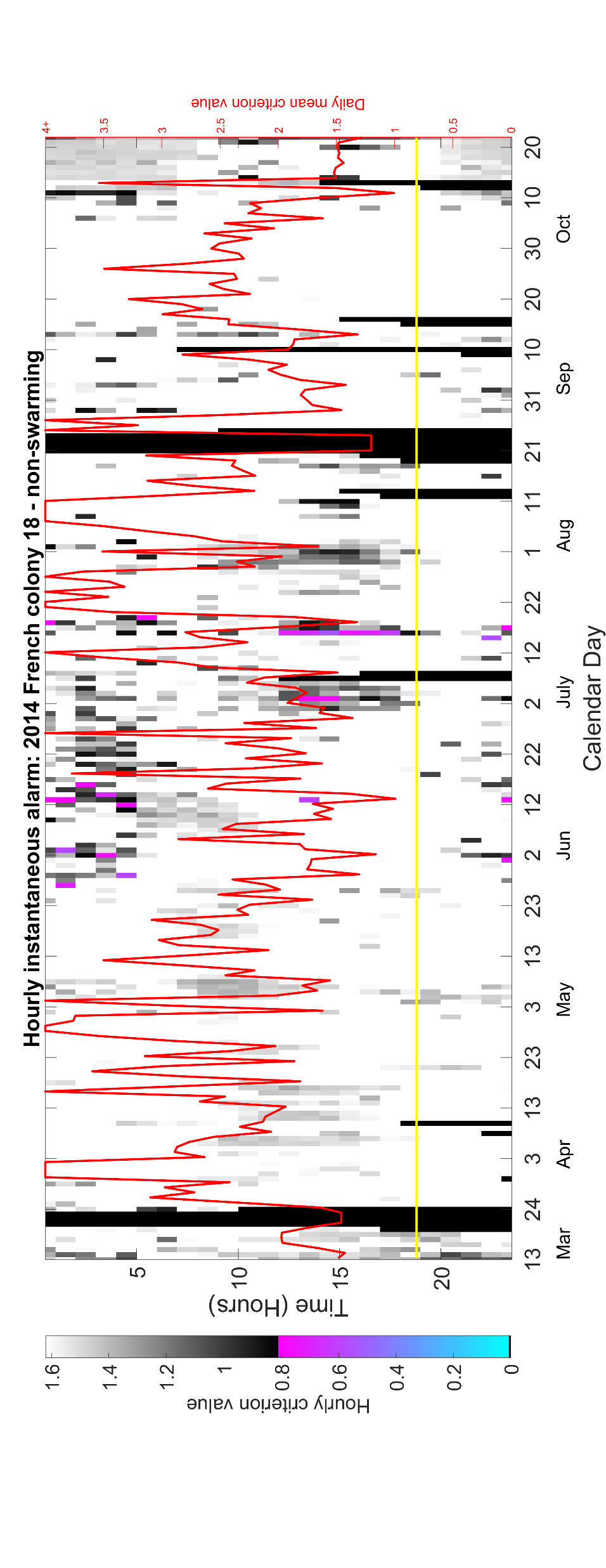

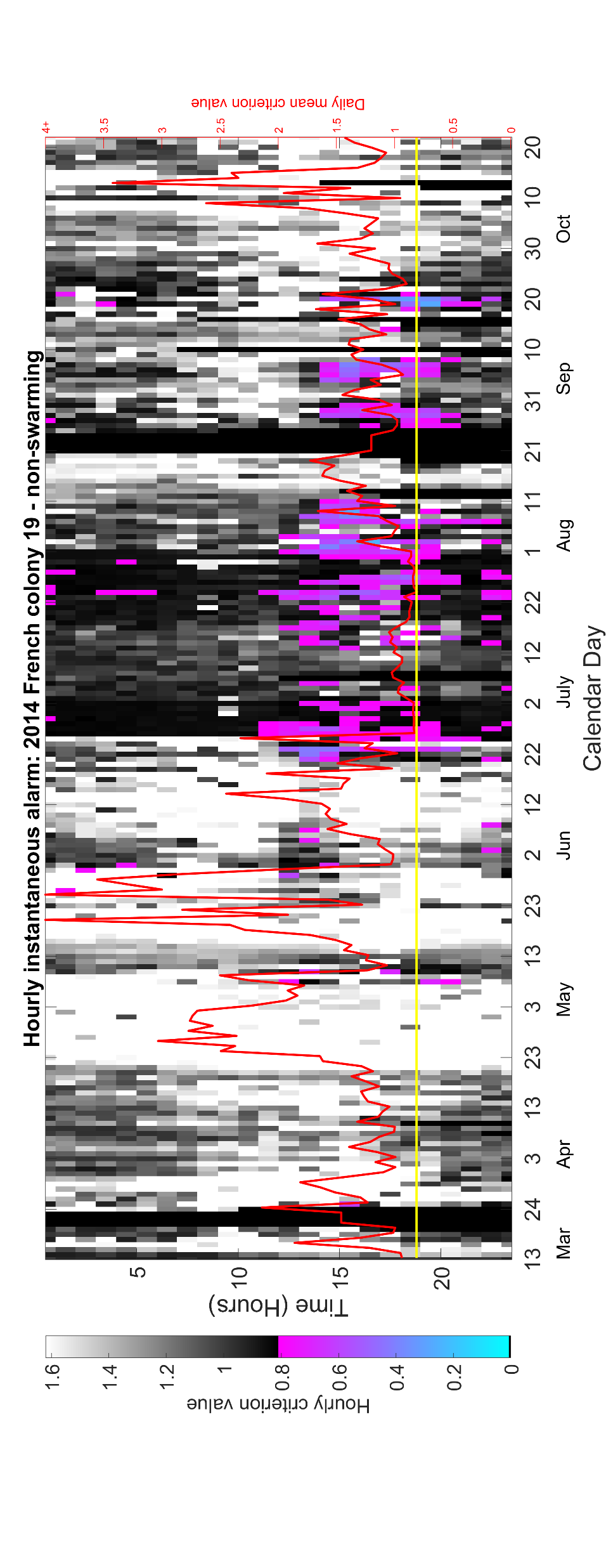

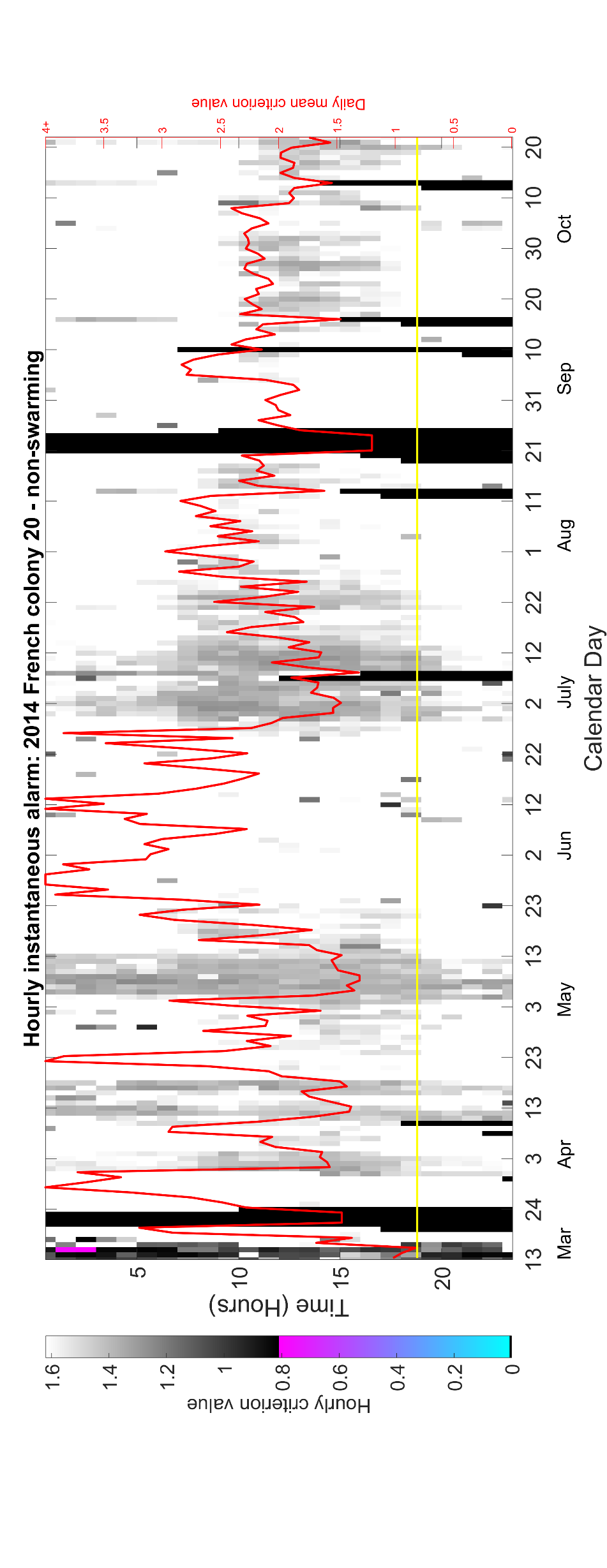

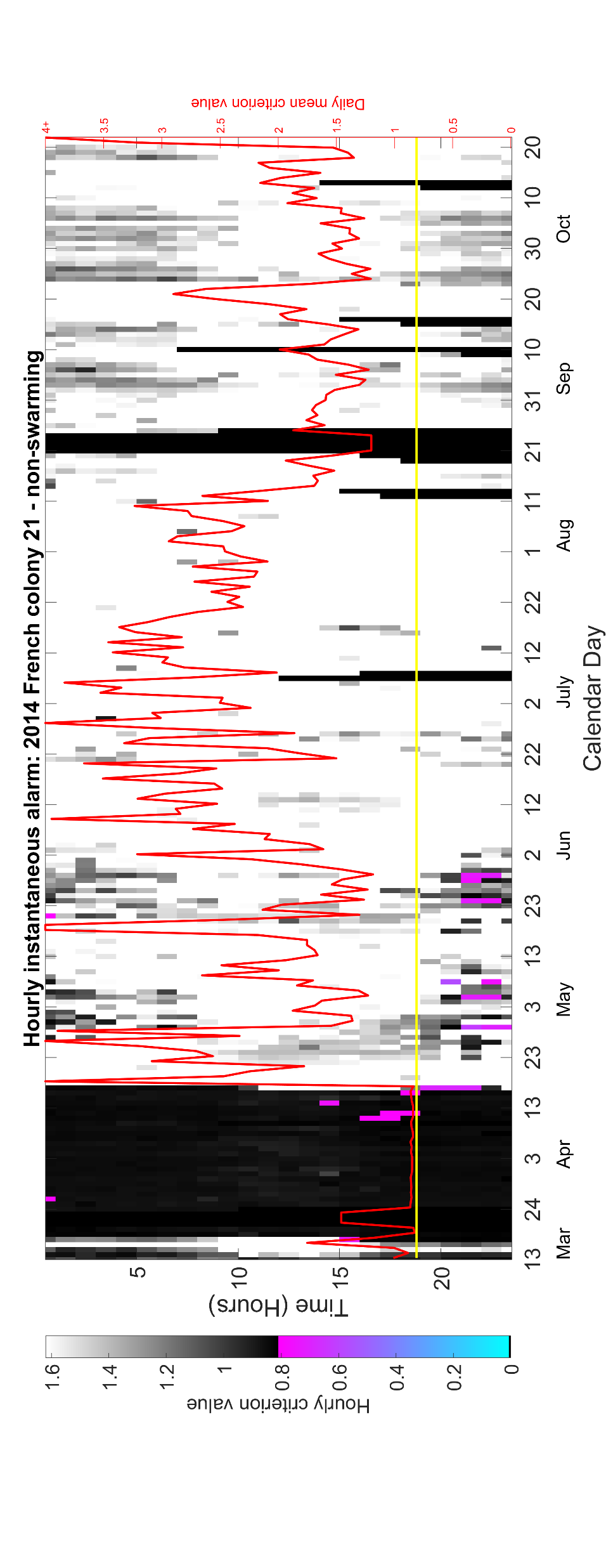

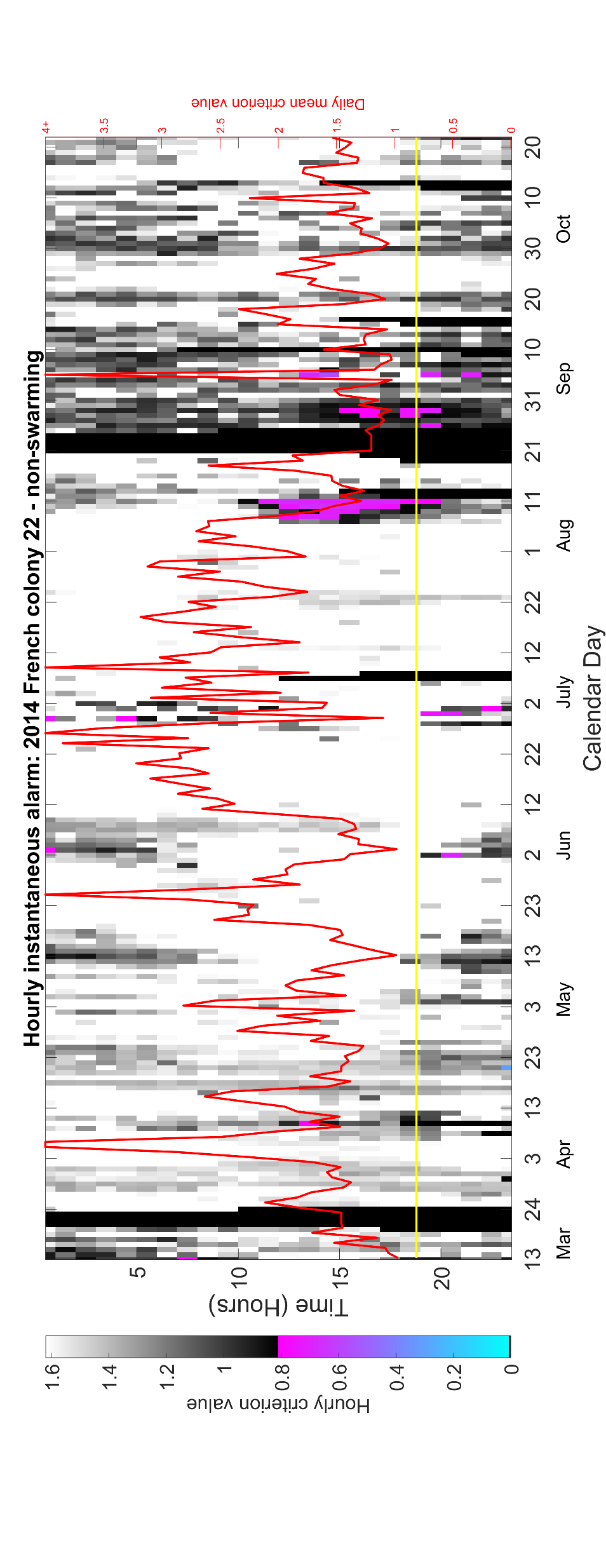


*Fig S8:* ***Non-Swarming Colony.***

***Instantaneous alarm for 2014 French colony 16*** *shown from the 13^th^ March until the 20^th^ October 2014. All figure parameters are identical to that described in Fig S1.*

*Fig S9:* ***Non-Swarming Colony.***

***Instantaneous alarm for 2014 French colony 17*** *shown from the 13^th^ March until the 20^th^ October 2014. All figure parameters are identical to that described in Fig S1.*

*Fig S10:* ***Non-Swarming Colony.***

***Instantaneous alarm for 2014 French colony 18*** *shown from the 13^th^ March until the 20^th^ October 2014. All figure parameters are identical to that described in Fig S1.*

*Fig S11:* ***Non-Swarming Colony.***

***Instantaneous alarm for 2014 French colony 19*** *shown from the 13^th^ March until the 20^th^ October 2014. All figure parameters are identical to that described in Fig S1.*

*Fig S12:* ***Non-Swarming Colony.***

***Instantaneous alarm for 2014 French colony 20*** *shown from the 13^th^ March until the 20^th^ October 2014. All figure parameters are identical to that described in Fig S1.*

*Fig S13:* ***Non-Swarming Colony.***

***Instantaneous alarm for 2014 French colony 21*** *shown from the 13^th^ March until the 20^th^ October 2014. All figure parameters are identical to that described in Fig S1.*

*Fig S14:* ***Non-Swarming Colony.***

***Instantaneous alarm for 2014 French colony 22*** *shown from the 13^th^ March until the 20^th^ October 2014. All figure parameters are identical to that described in Fig S1.*

**Instantaneous spectra alarm for colonies that swarmed**

In this section, Fig S15 – S32 show the alarm based on instantaneous spectra for various colonies monitored across the 2014 and 2015 active seasons. The plots have been grouped together because they represent colonies that **swarmed**.


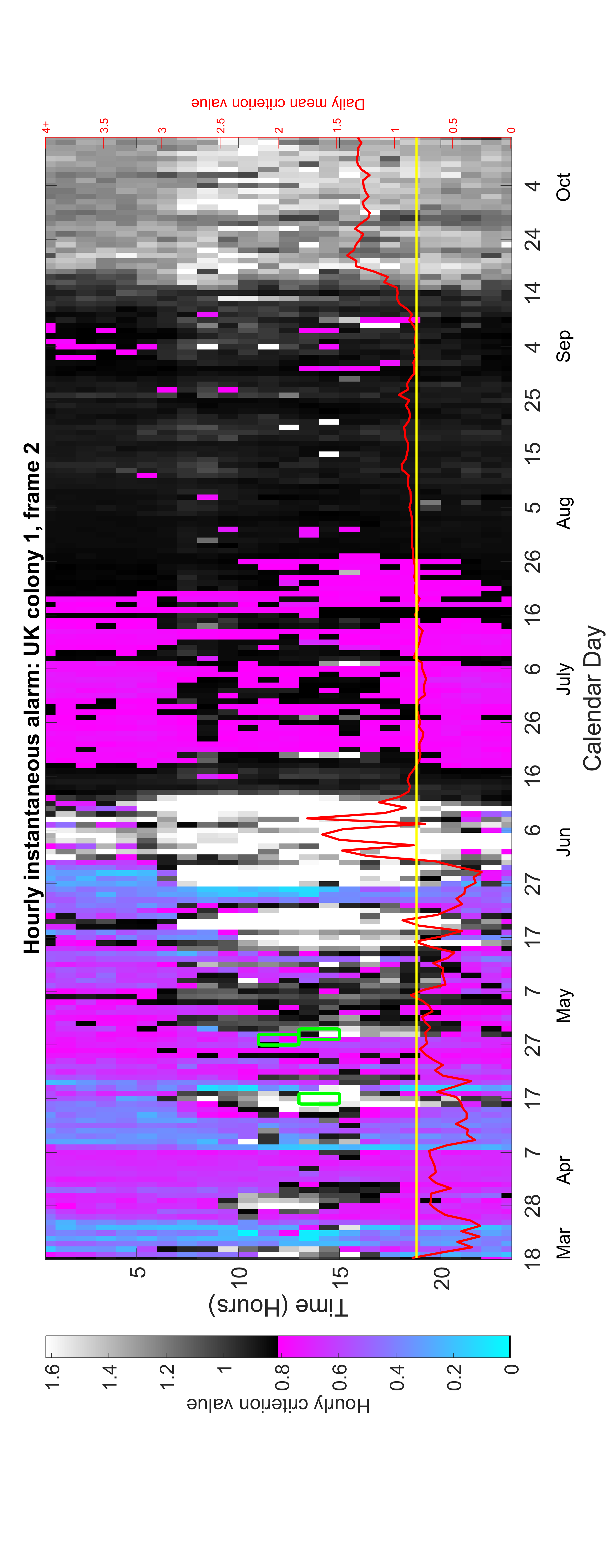

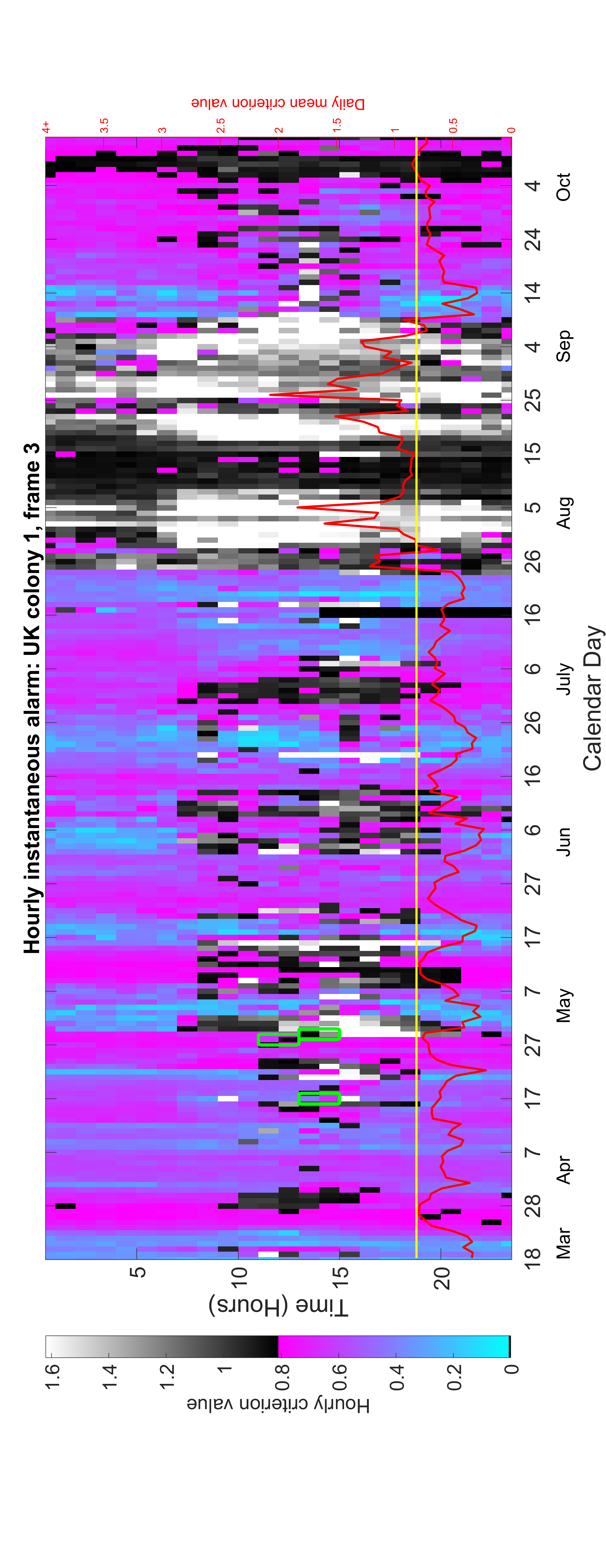

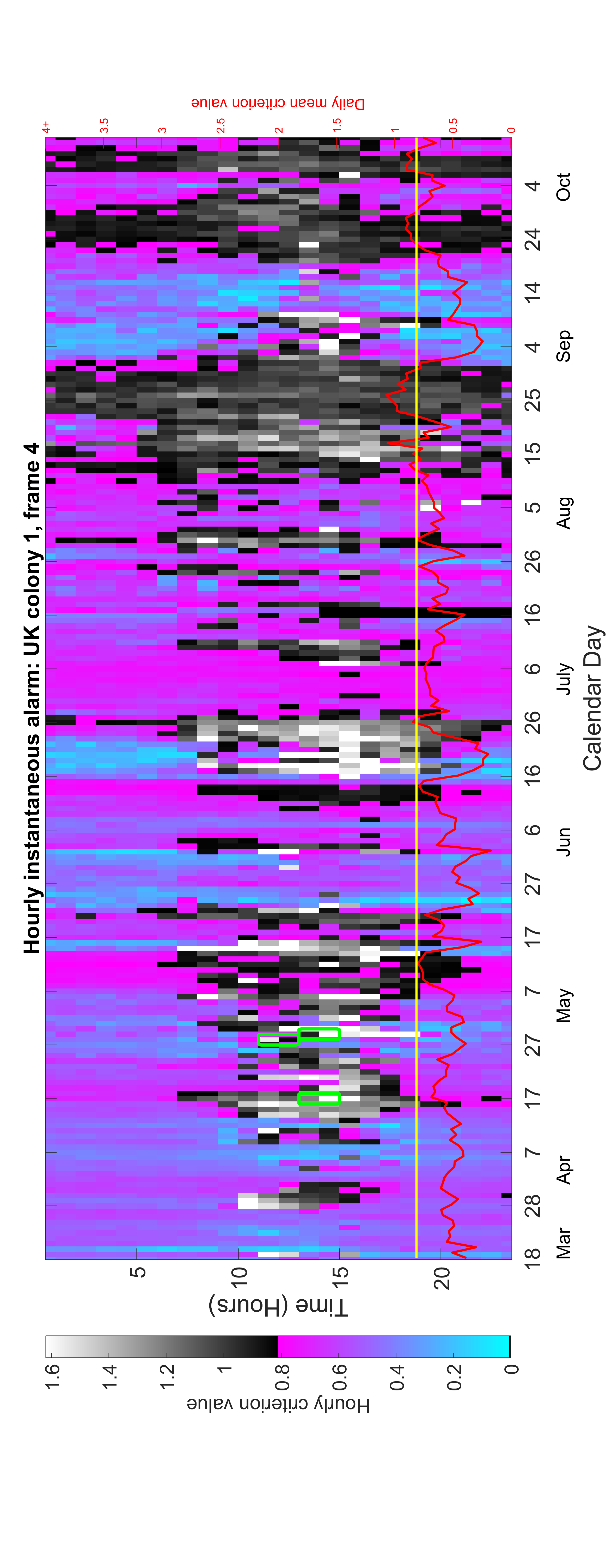

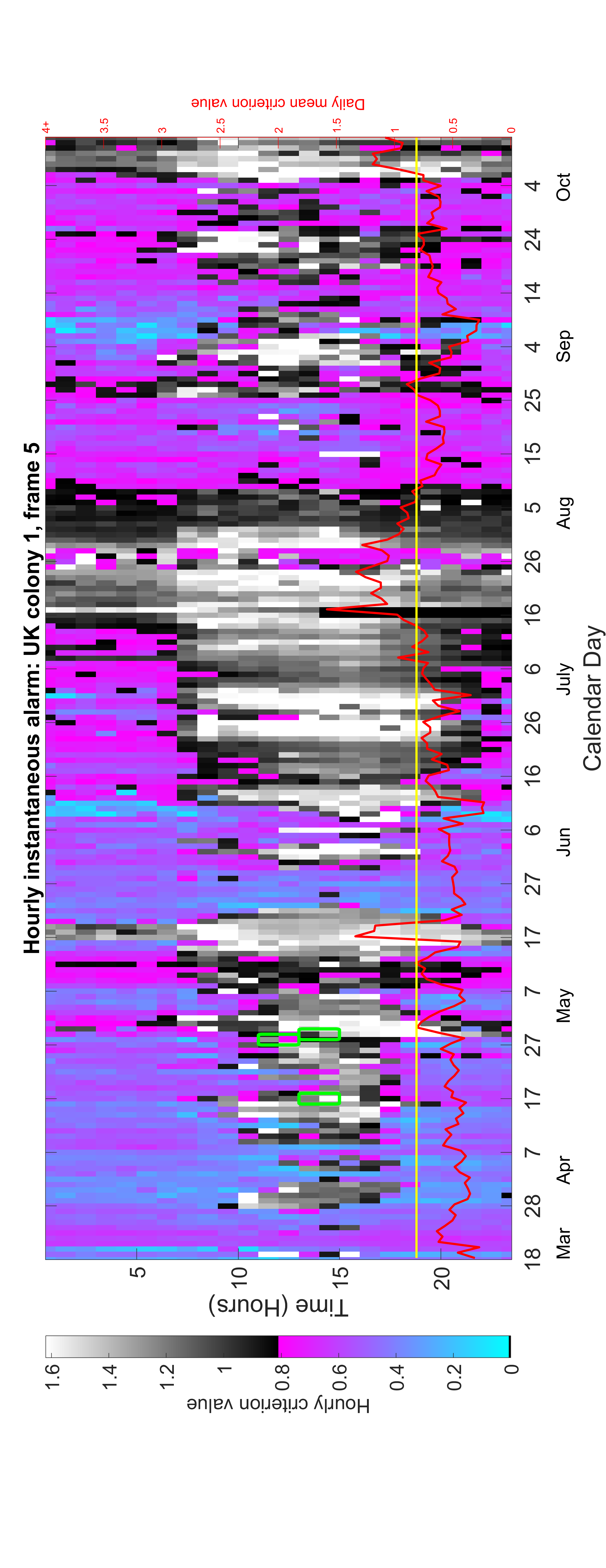

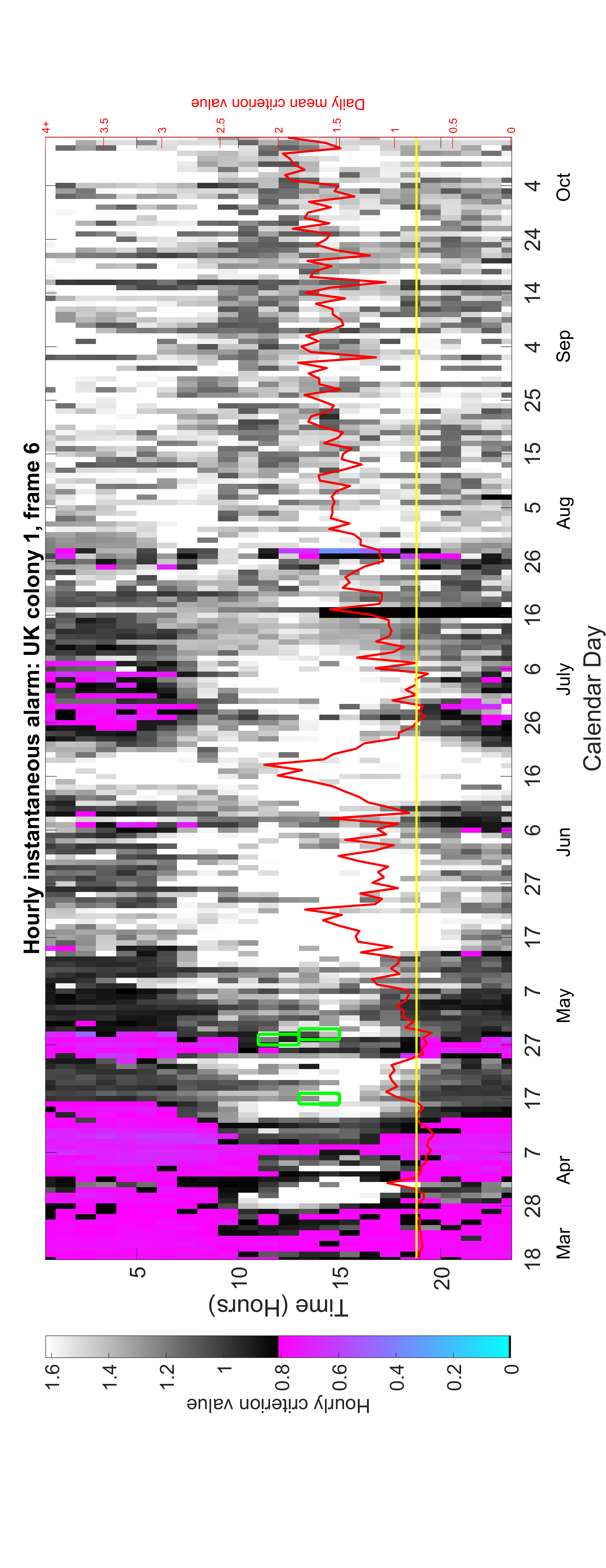

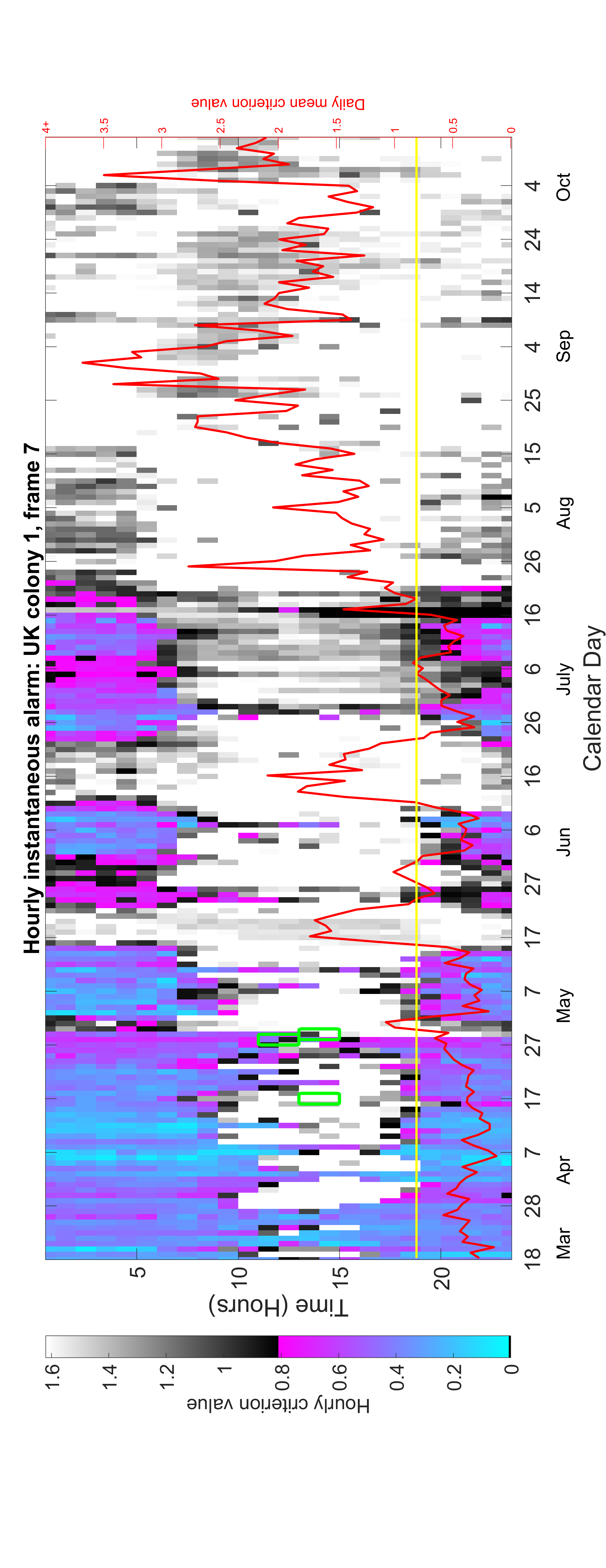

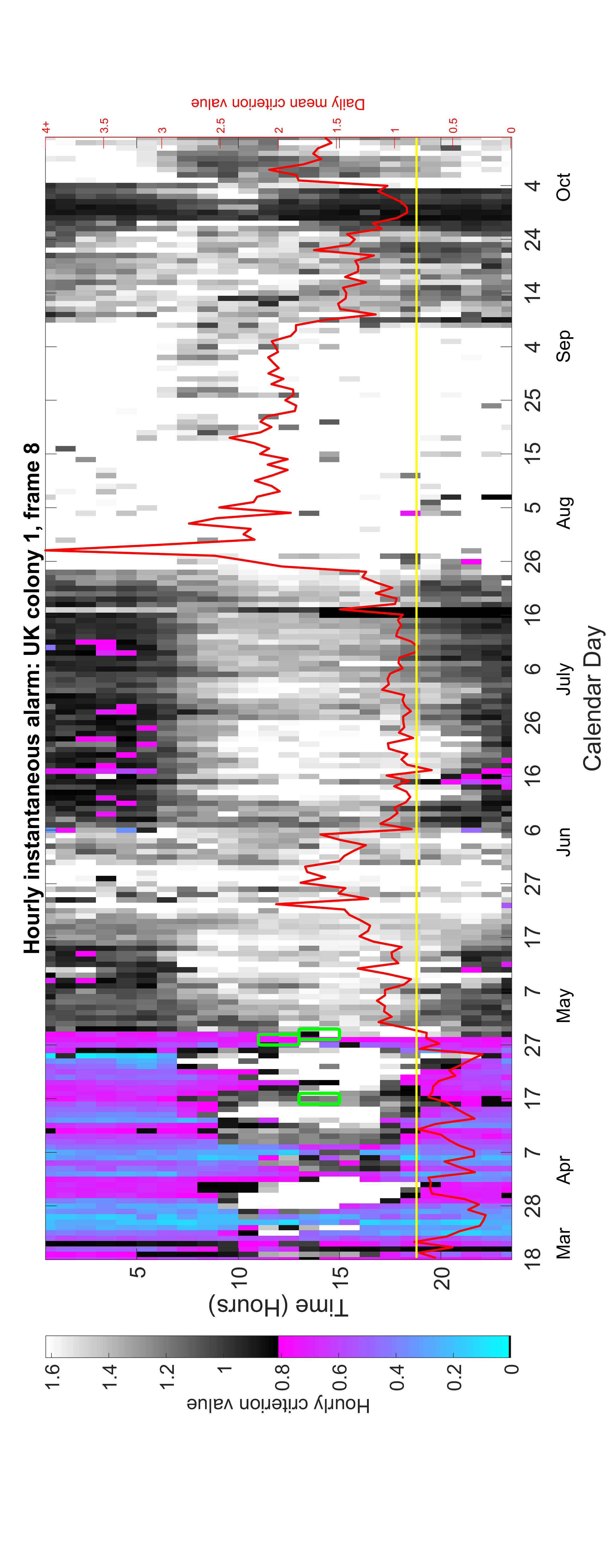

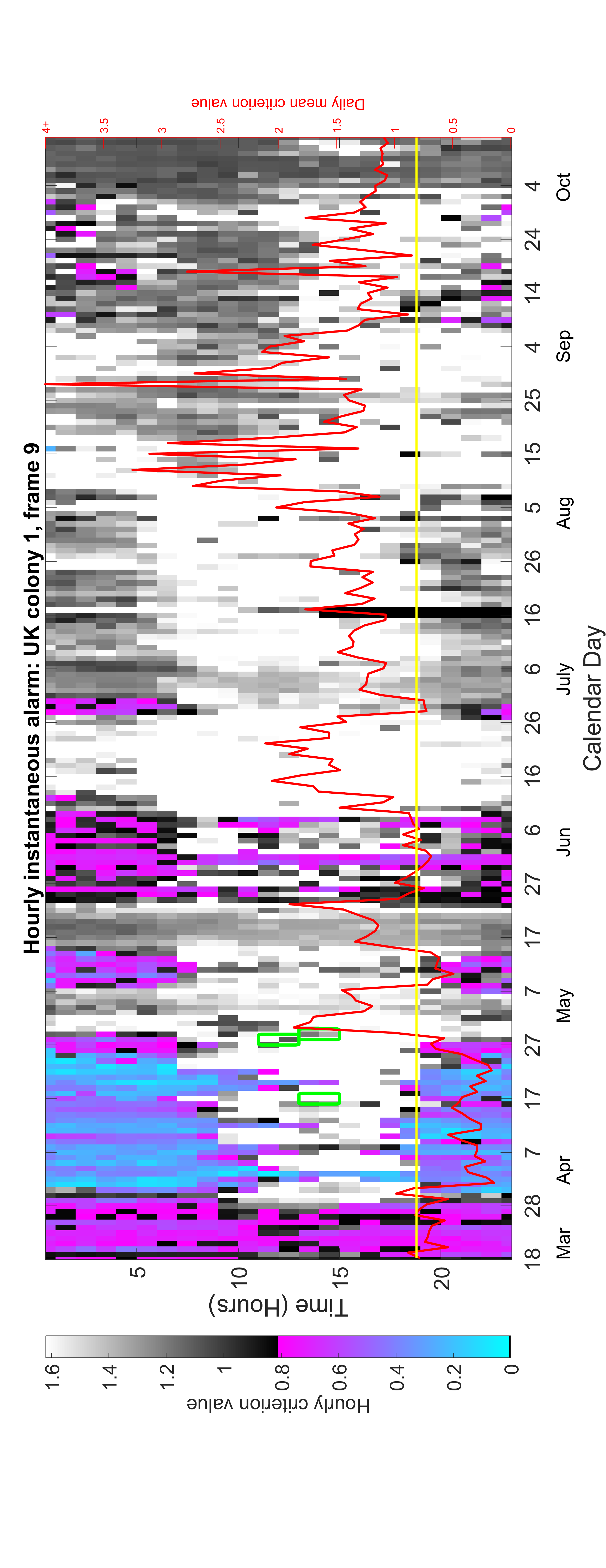

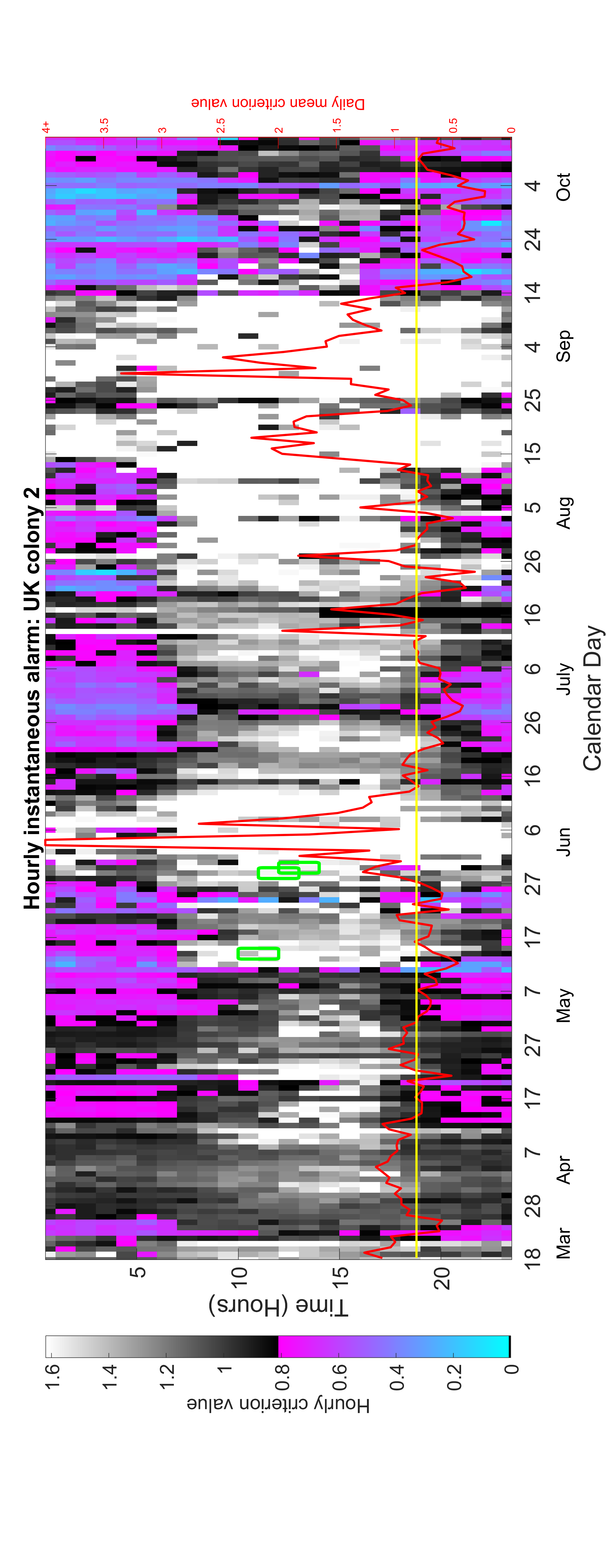

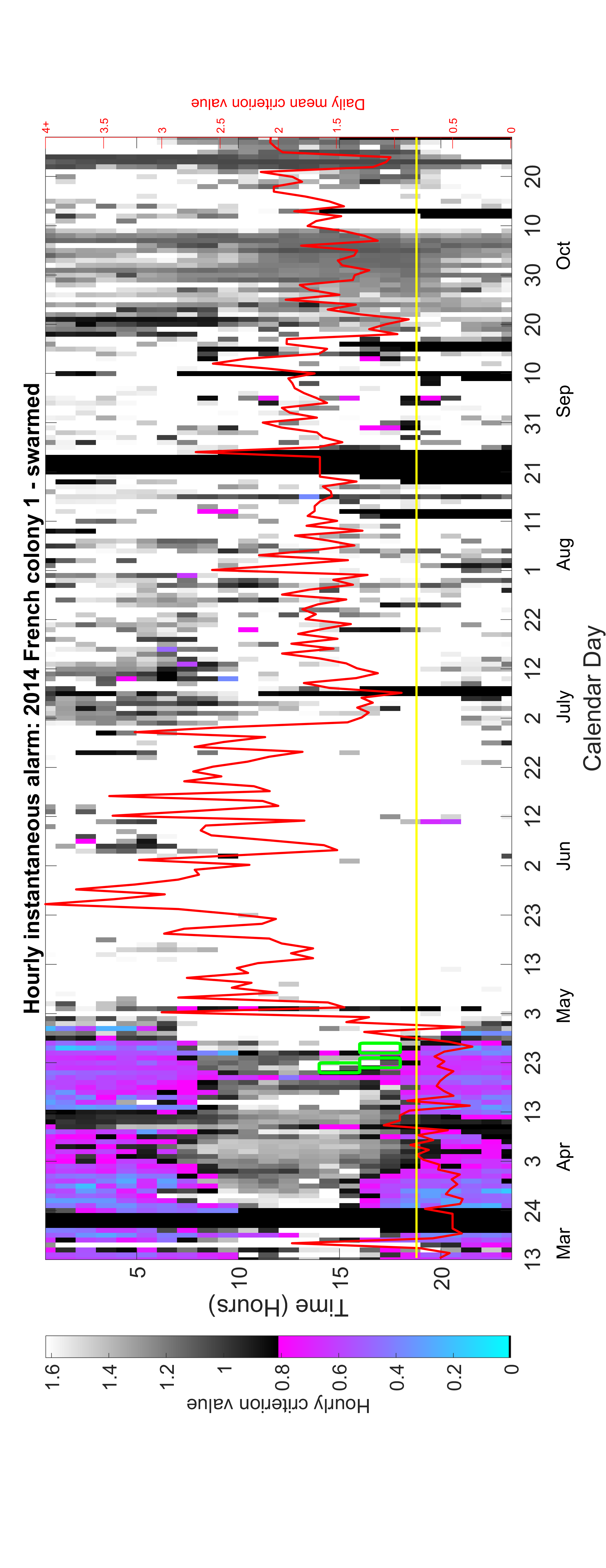

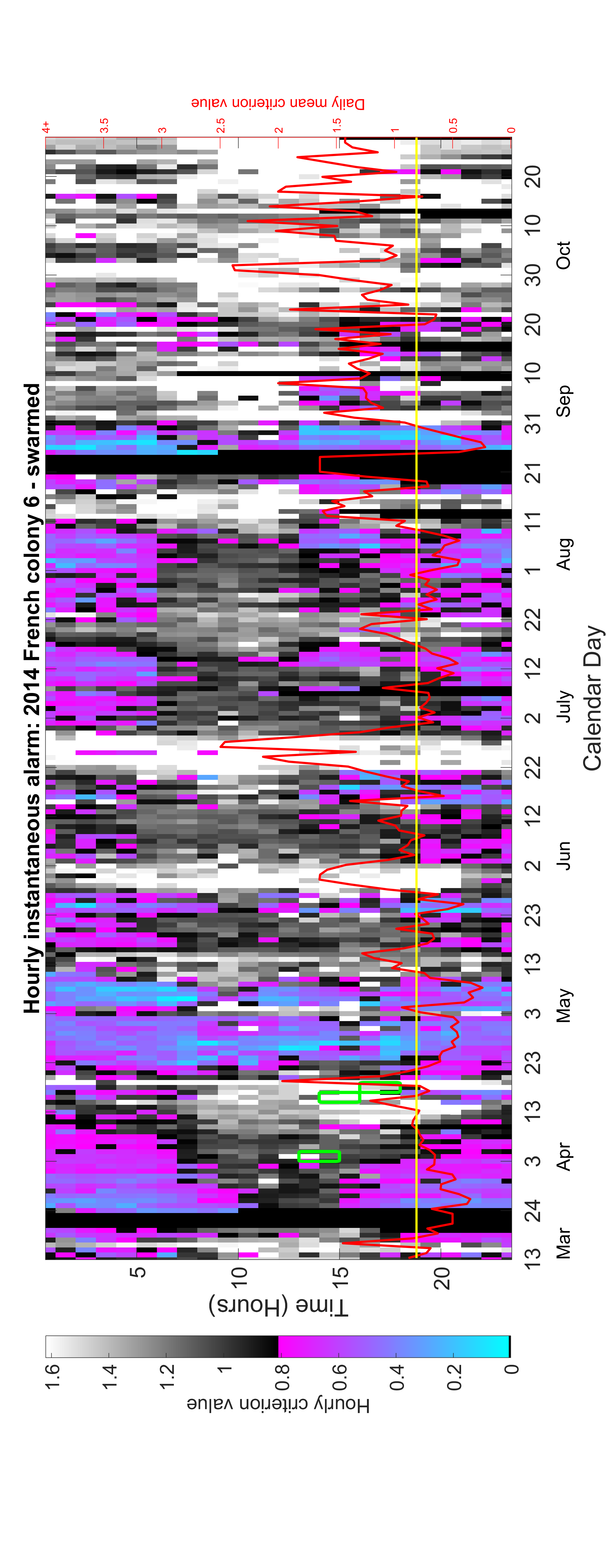

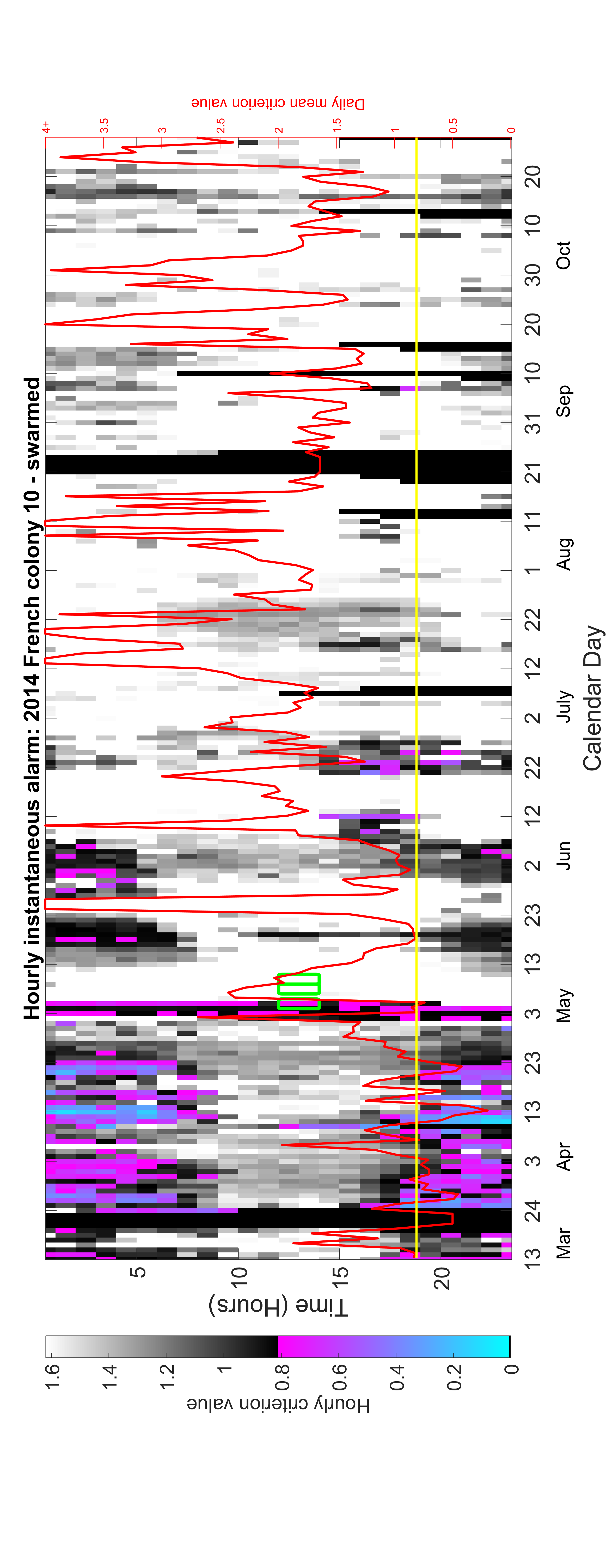

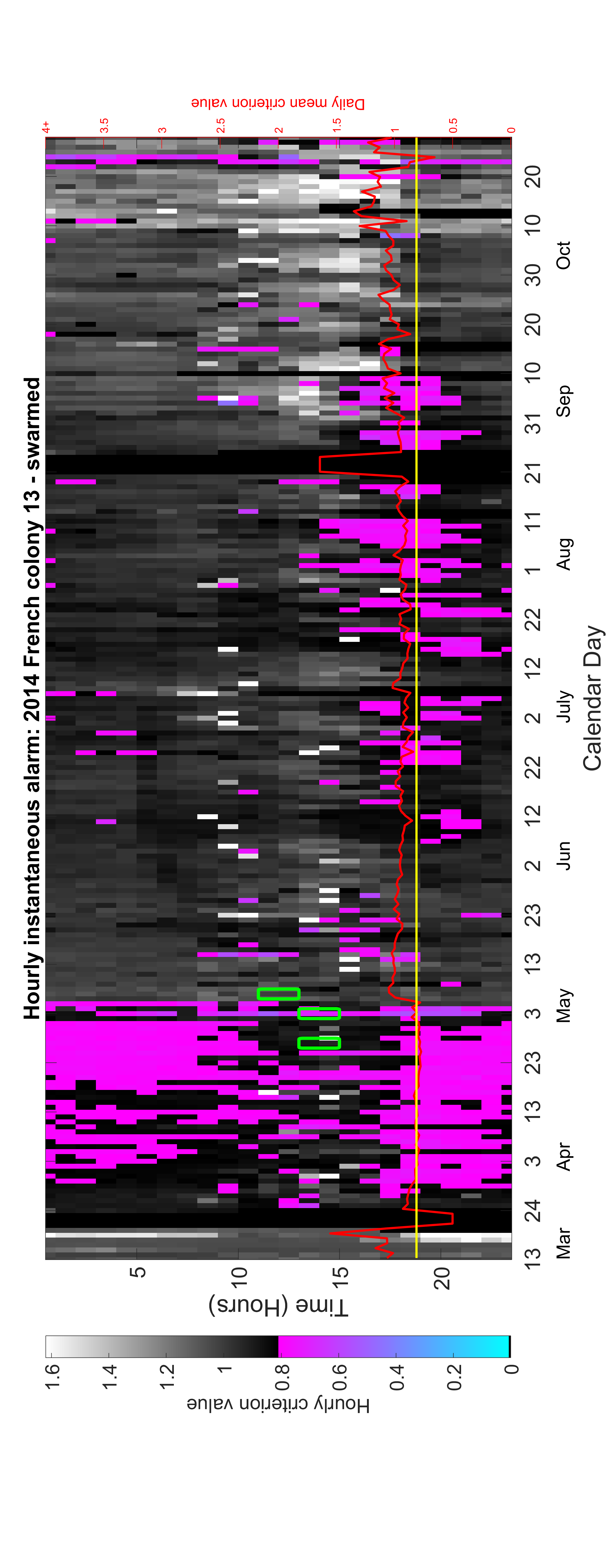

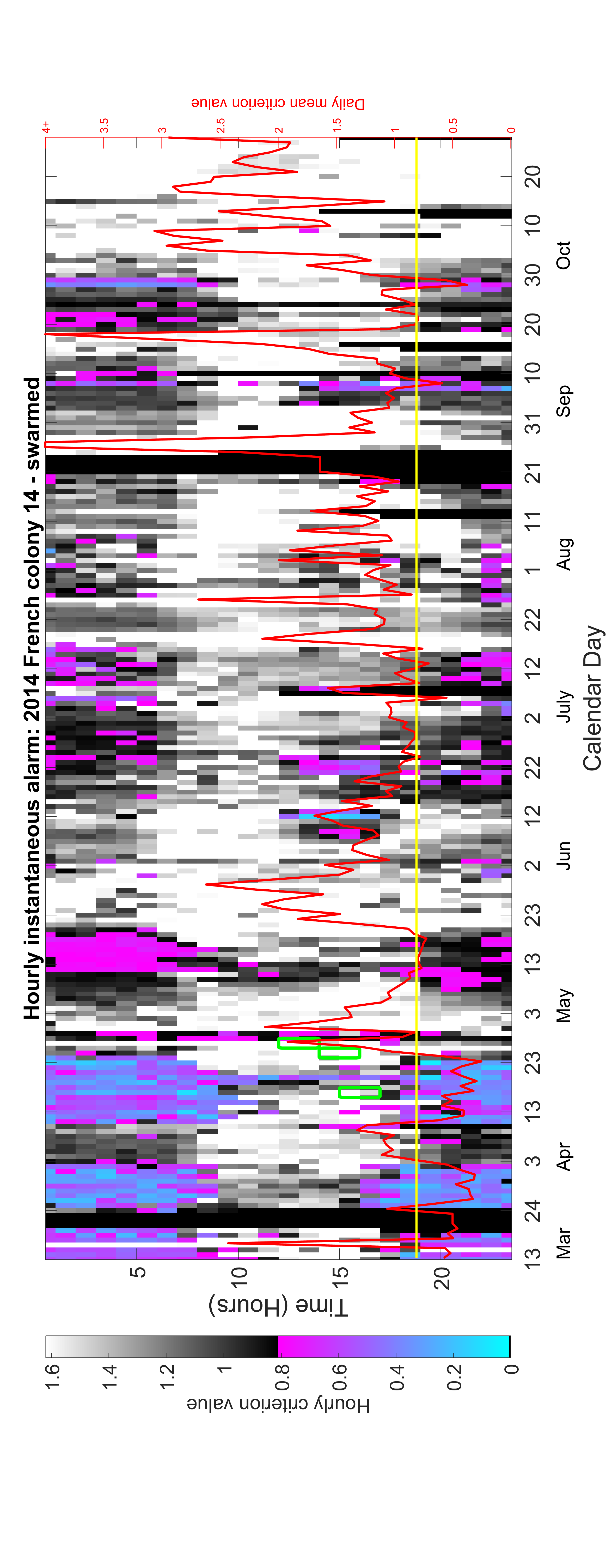

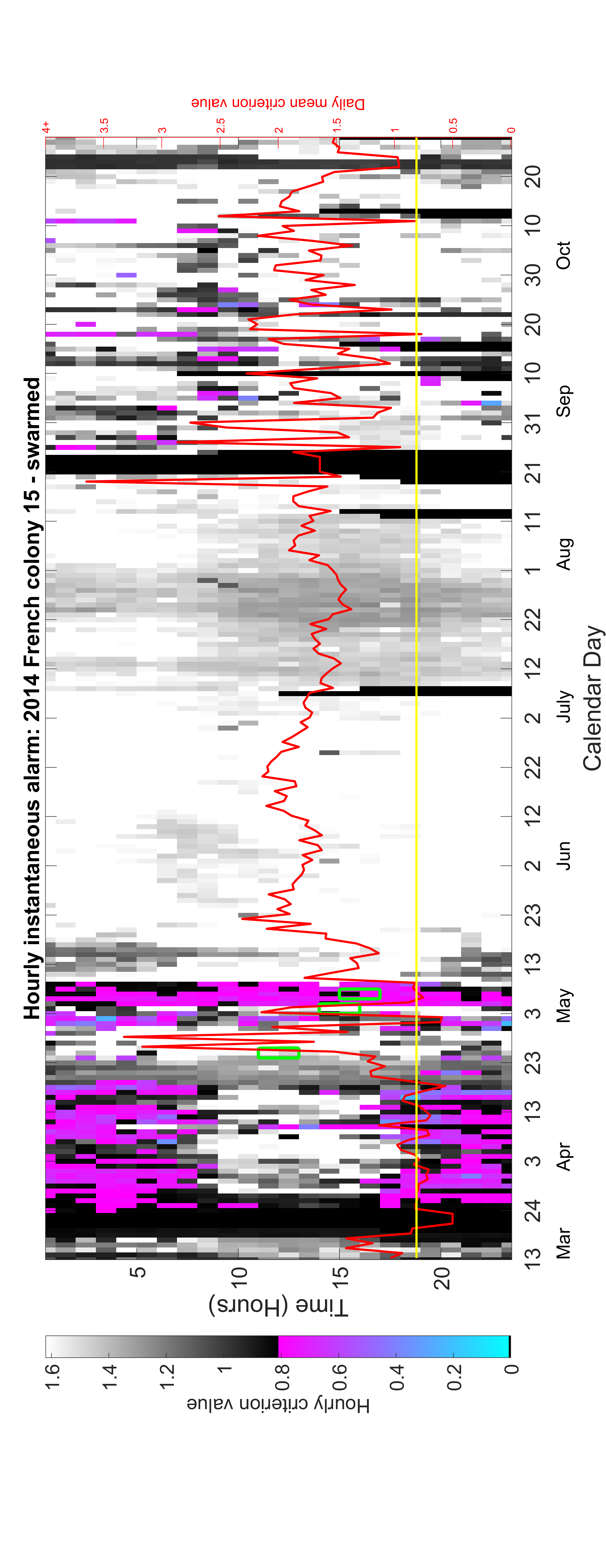

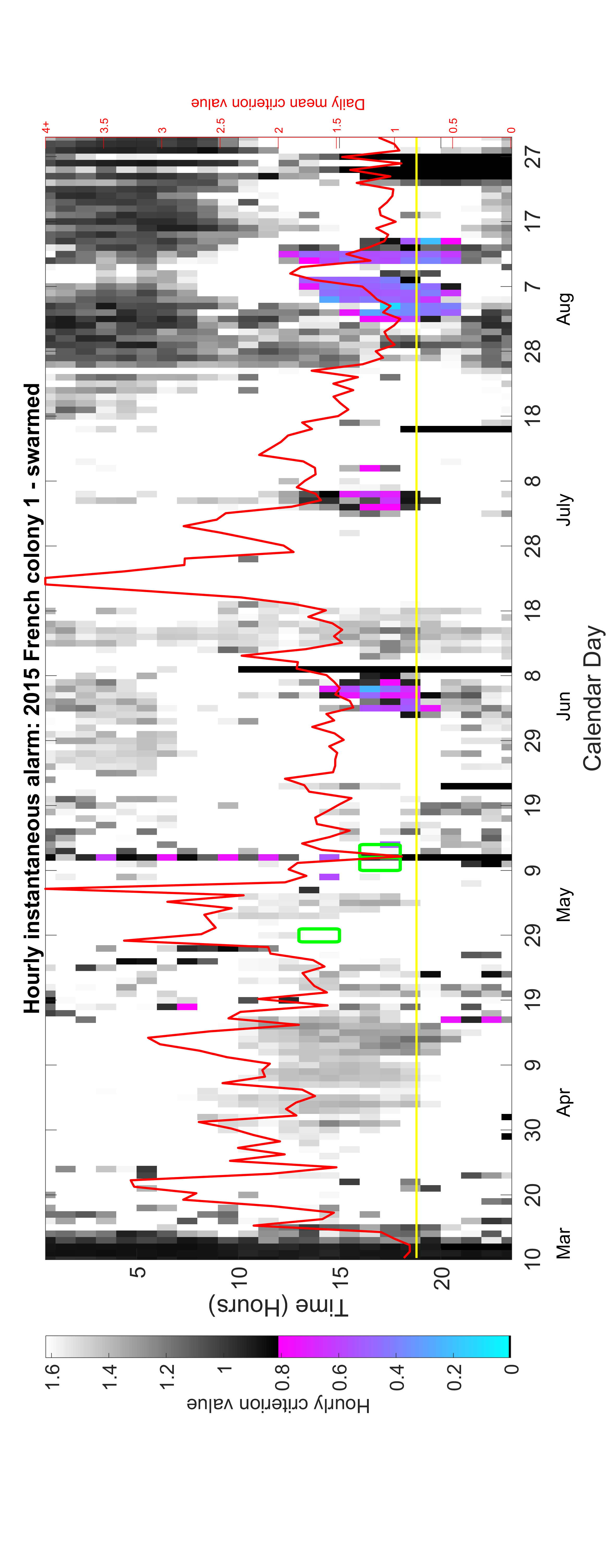

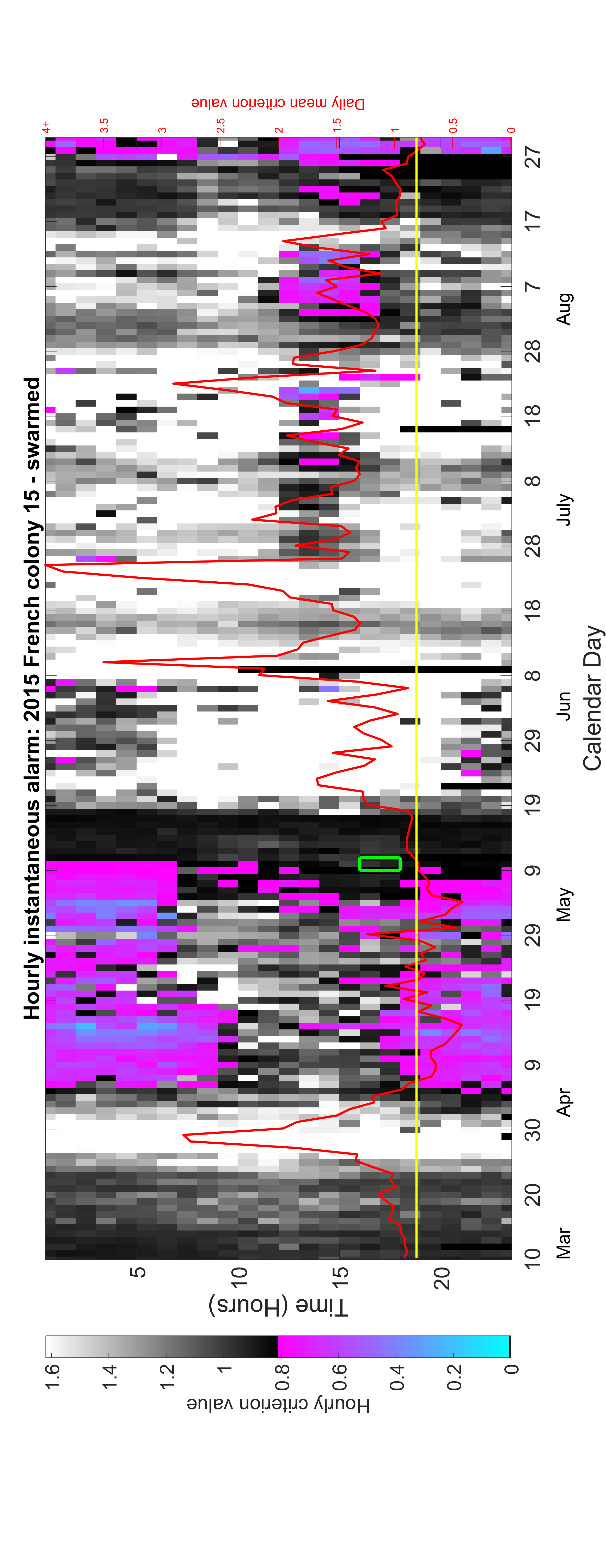

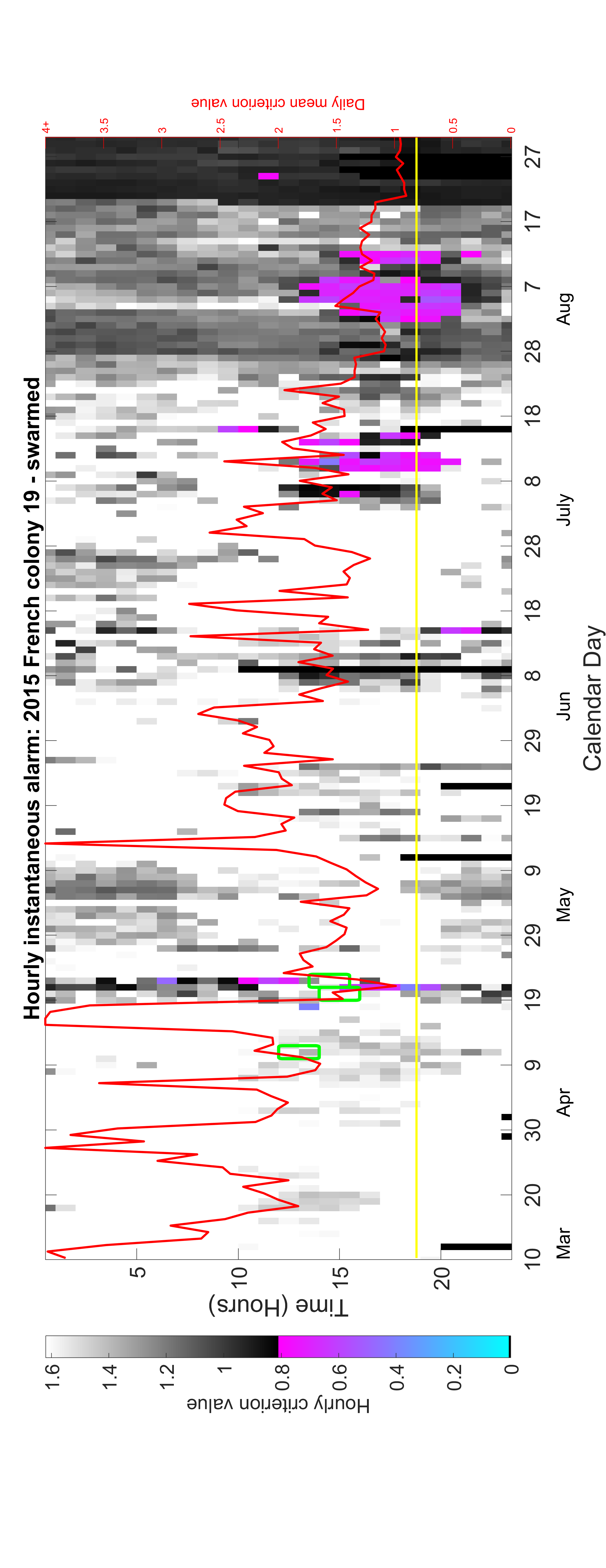


*Fig S15:* ***Swarming Colony.***

*Instantaneous alarm for the second frame of the 2014 UK colony 1, shown from the 18th March until the colony 14^th^ October. The colour coding has been split. Greyscale and coloured pixel intensity denote alarm values above (non-swarming state) and below (swarming state) the threshold, from pink to blue as the criterion approaches the swarming centroid. Green rectangles show the occurrence of swarms within this dataset. Superimposed is another set of axis showing the average of the previous night’s alarm taken between midnight and 5am, with the yellow line displaying the optimum alarm threshold.*

*Fig S16:* ***Swarming Colony.***

*Instantaneous alarm for the third frame of the 2014 UK colony 1, shown from the 18th March until the colony, died in October. The colour coding and plot features are identical to that of Fig S15.*

*Fig S17:* ***Swarming Colony.***

*Instantaneous alarm for the forth frame of the 2014 UK colony 1, shown from the 18th March until the colony, died in October. The colour coding and plot features are identical to that of Fig S15.*

*Fig S18:* ***Swarming Colony.***

*Instantaneous alarm for the fifth frame of the 2014 UK colony 1, shown from the 18th March until the colony, died in October. The colour coding and plot features are identical to that of Fig S15.*

*Fig S19:* ***Swarming Colony.***

*Instantaneous alarm for the sixth frame of the 2014 UK colony 1, shown from the 18th March until the colony, died in October. The colour coding and plot features are identical to that of Fig S15.*

*Fig S20:* ***Swarming Colony.***

*Instantaneous alarm for the seventh frame of the 2014 UK colony 1, shown from the 18th March until the colony, died in October. The colour coding and plot features are identical to that of Fig S15.*

*Fig 21:* ***Swarming Colony.***

*Instantaneous alarm for the eighth frame of the 2014 UK colony 1, shown from the 18th March until the colony, died in October. The colour coding and plot features are identical to that of Fig S15.*

*Fig S22:* ***Swarming Colony.***

*Instantaneous alarm for the ninth frame of the 2014 UK colony 1, shown from the 18^th^ March until the 14^th^ October. The colour coding and plot features are identical to that of Fig S15.*

*Fig S23:* ***Swarming Colony.***

*Instantaneous alarm for the 2014 UK colony 2, shown from the 18^th^ March until the 14^th^ October. The colour coding and plot features are identical to that of Fig S15.*

*Fig S24:* ***Swarming Colony.***

*Instantaneous alarm for the 2014 French colony 1, shown from the 13th March until the 30^th^ October. The colour coding and plot features are identical to that of Fig S15.*

*Fig S25:* ***Swarming Colony.***

*Instantaneous alarm for the 2014 French colony 6, shown from the 13th March until the 30^th^ October. The colour coding and plot features are identical to that of Fig S15.*

*Fig S26:* ***Swarming Colony.***

*Instantaneous alarm for the 2014 French colony 10, shown from the 13th March until the 30^th^ October. The colour coding and plot features are identical to that of Fig S15.*

*Fig S27:* ***Swarming Colony.***

*Instantaneous alarm for the 2014 French colony 13, shown from the 13th March until the 30^th^ October. The colour coding and plot features are identical to that of Fig S15.*

*Fig S28:* ***Swarming Colony.***

*Instantaneous alarm for the 2014 French colony 14, shown from the 13th March until the 30^th^ October. The colour coding and plot features are identical to that of Fig S15.*

*Fig S29:* ***Swarming Colony.***

*Instantaneous alarm for the 2014 French colony 15, shown from the 13th March until the 30^th^ October. The colour coding and plot features are identical to that of Fig S15.*

*Fig S30:* ***Swarming Colony.***

*Instantaneous alarm for the 2015 French colony 1, shown from the 13th March until the 30^th^ October. The colour coding and plot features are identical to that of Fig S15.*

*Fig S31:* ***Swarming Colony.***

*Instantaneous alarm for the 2015 French colony 15, shown from the 13th March until the 30^th^ October. The colour coding and plot features are identical to that of Fig S15.*

*Fig S32:* ***Swarming Colony.***

*Instantaneous alarm for the 2015 French colony 19, shown from the 13th March until the 30^th^ October. The colour coding and plot features are identical to that of Fig S15.*

**Similarity of swarming and non-swarming instantaneous spectra**

Fig S33: **The similarity that can occasionally take place between two instantaneous spectra** extracted from the swarming (red curve) and non-swarming (black curve) training databases is clearly demonstrated in the case of the two specific spectra showcased here.

**Spectral evolution alarm for colonies that did not swarm**

In this section, Fig S34 – S47 show the alarm based on the ten day evolution of spectra for various colonies monitored in France across the 2014 and 2015 active seasons. The plots have been grouped together because they represent colonies that **did not swarm**.


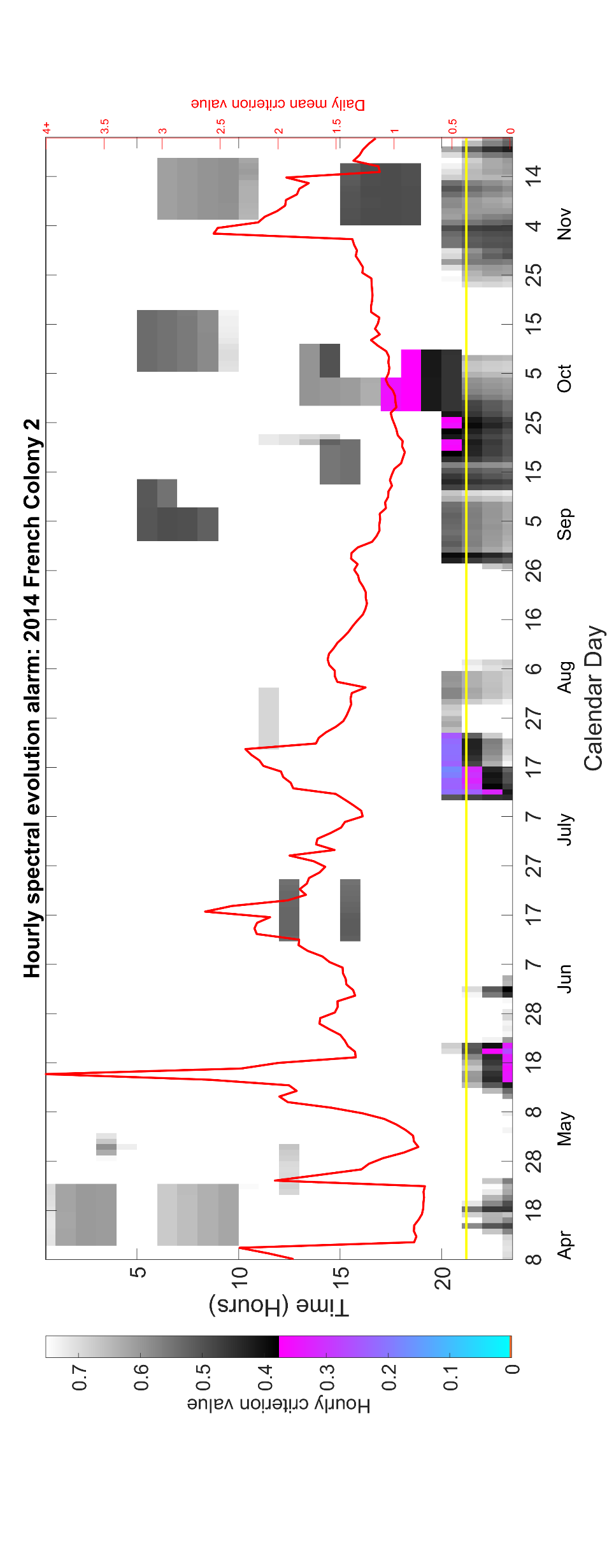

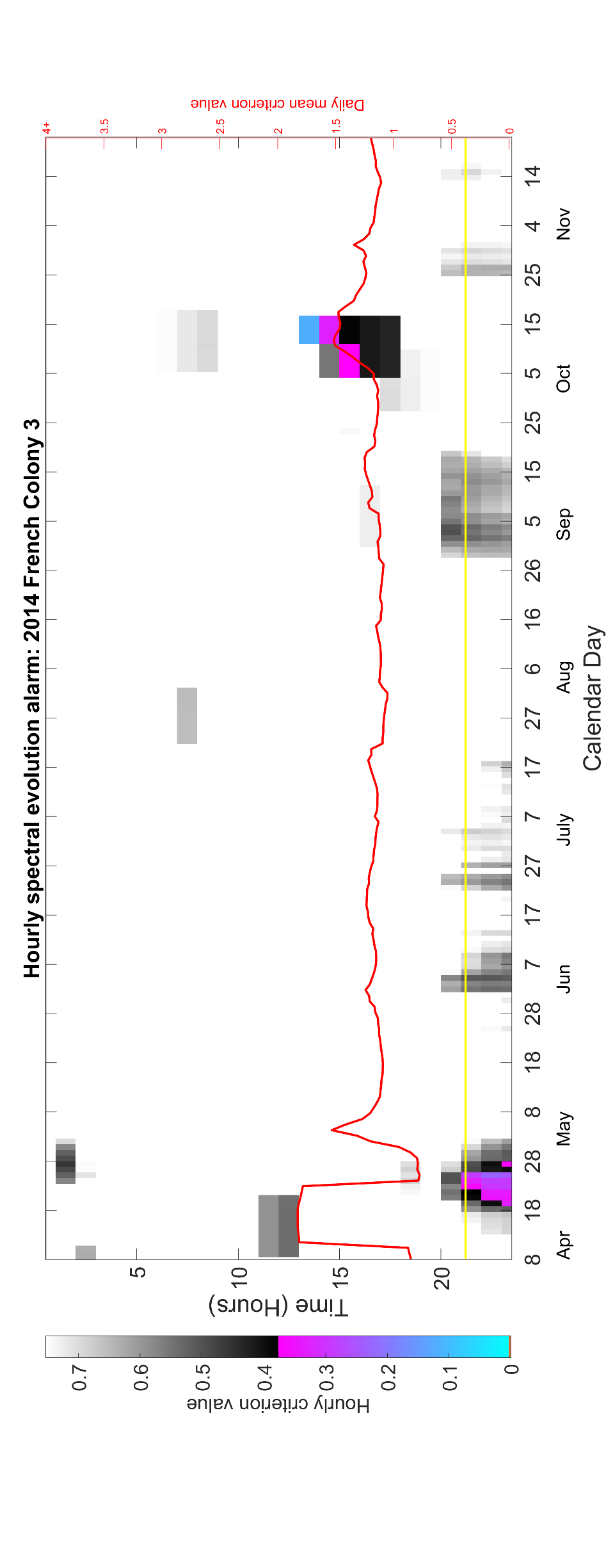

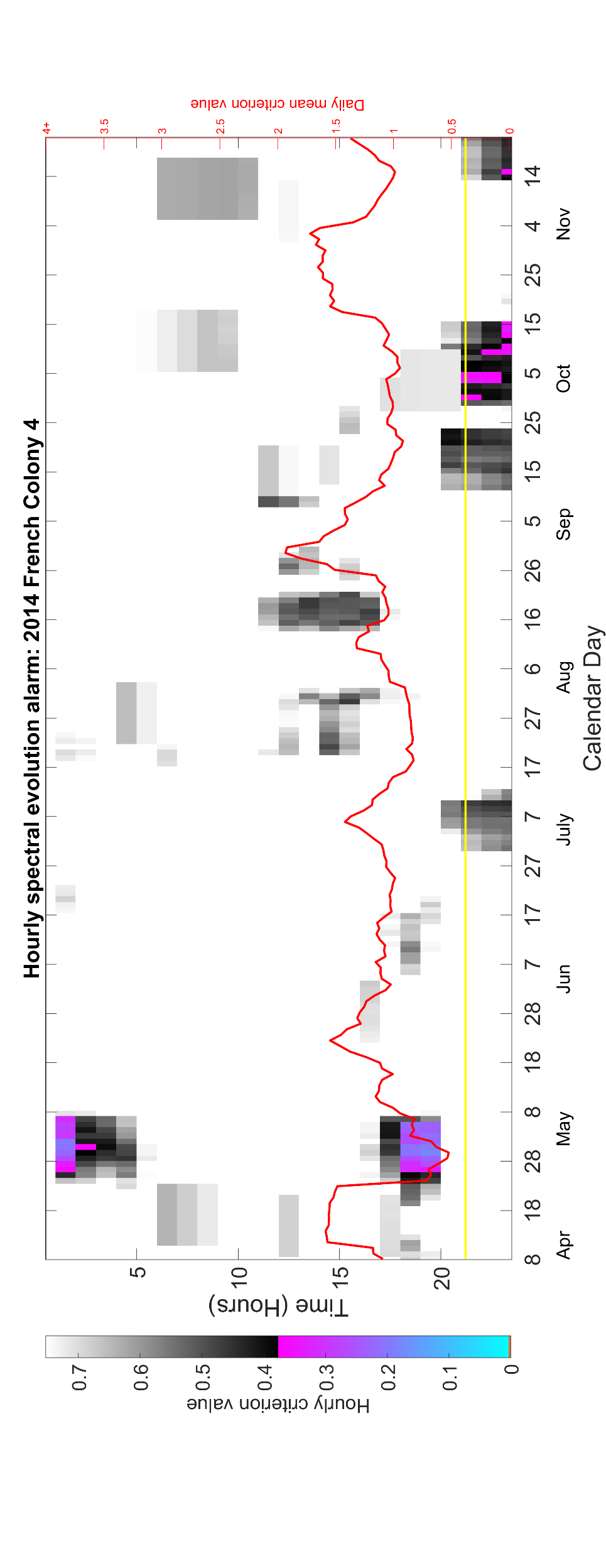

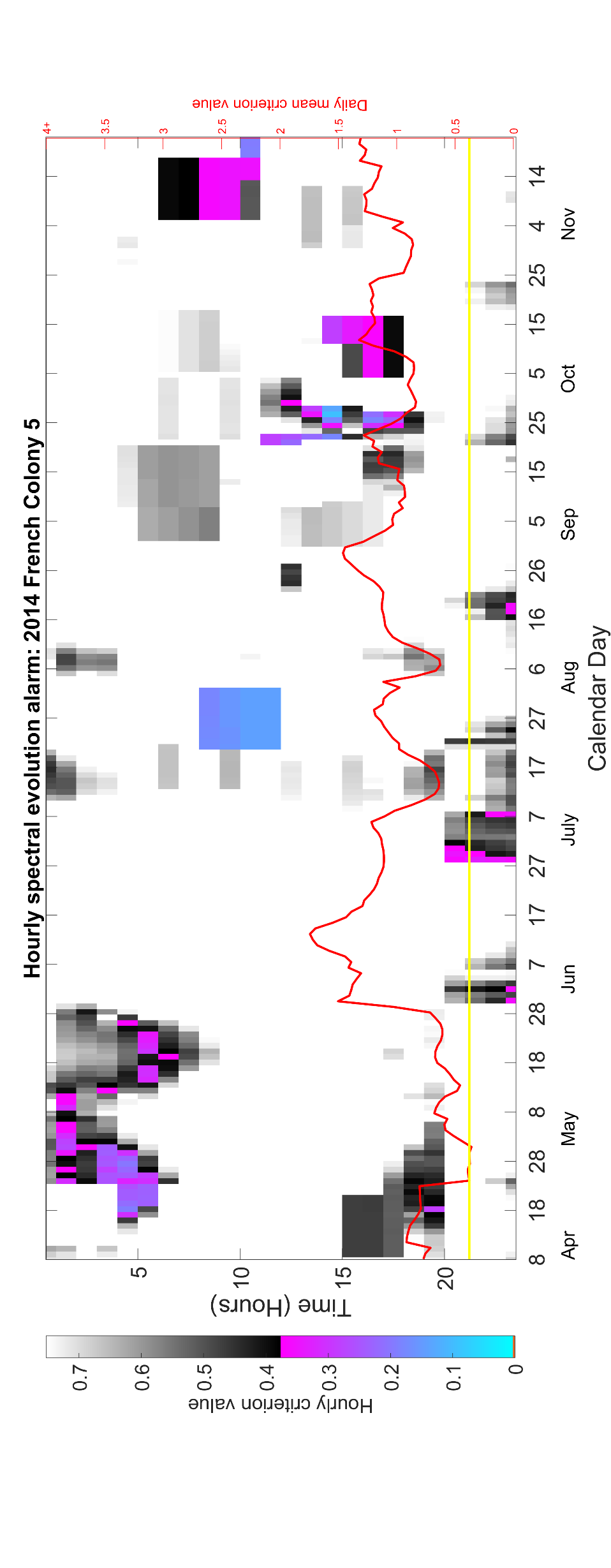

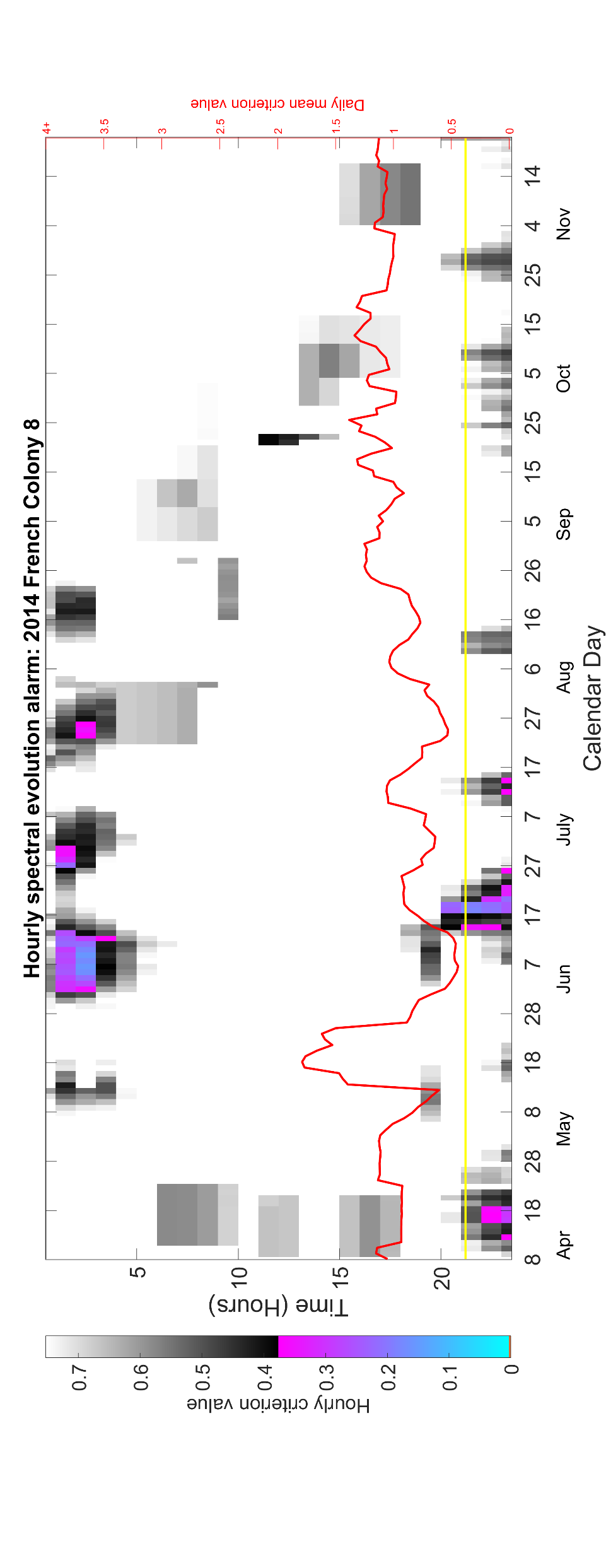

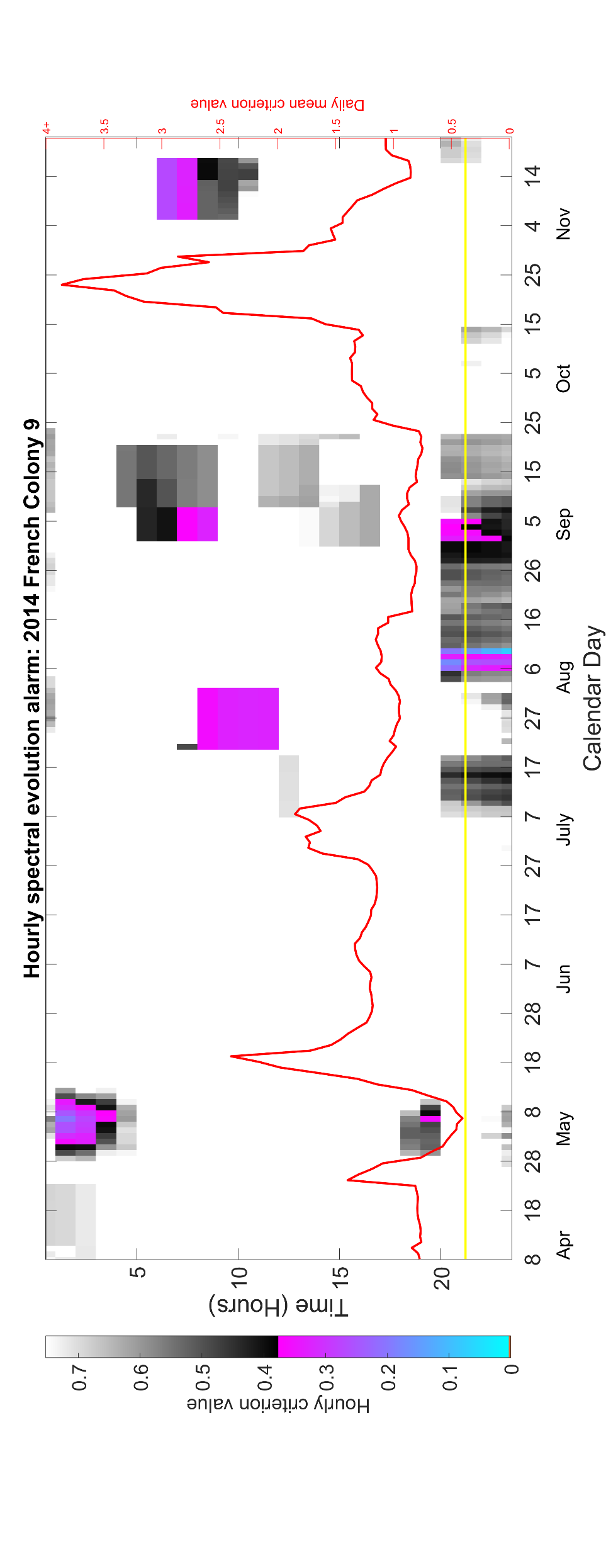

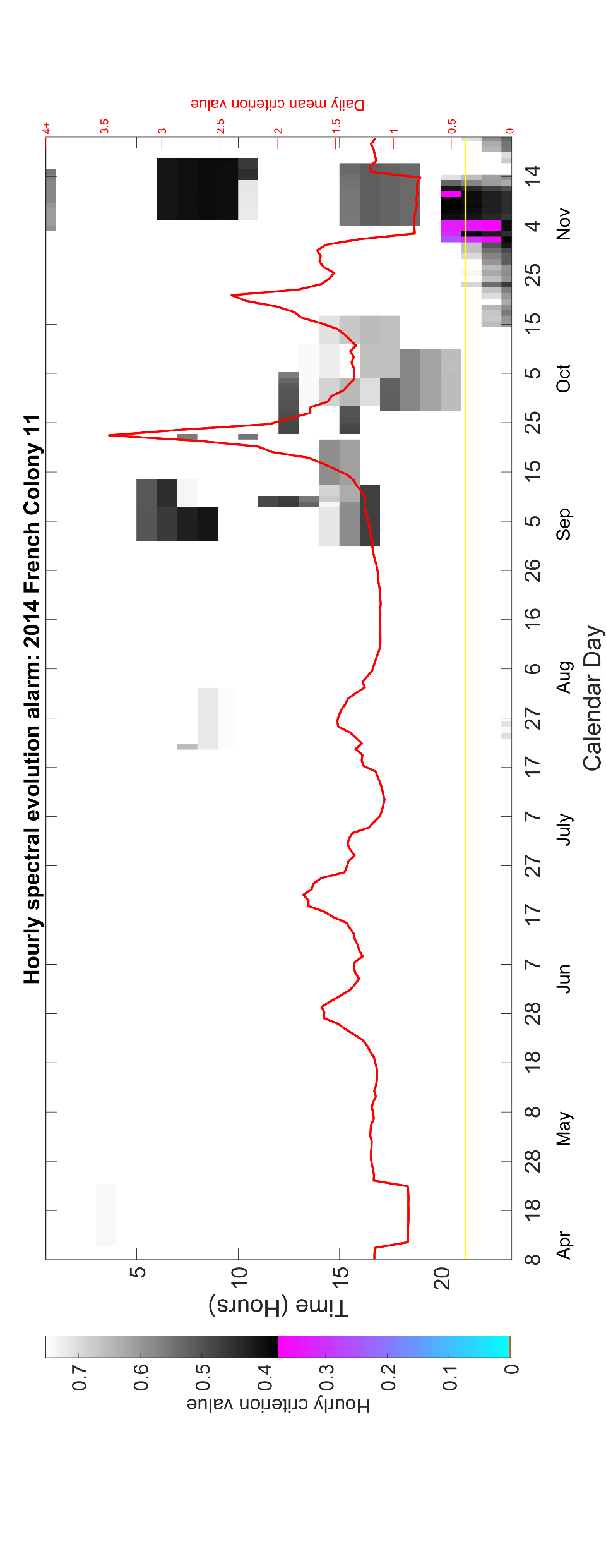

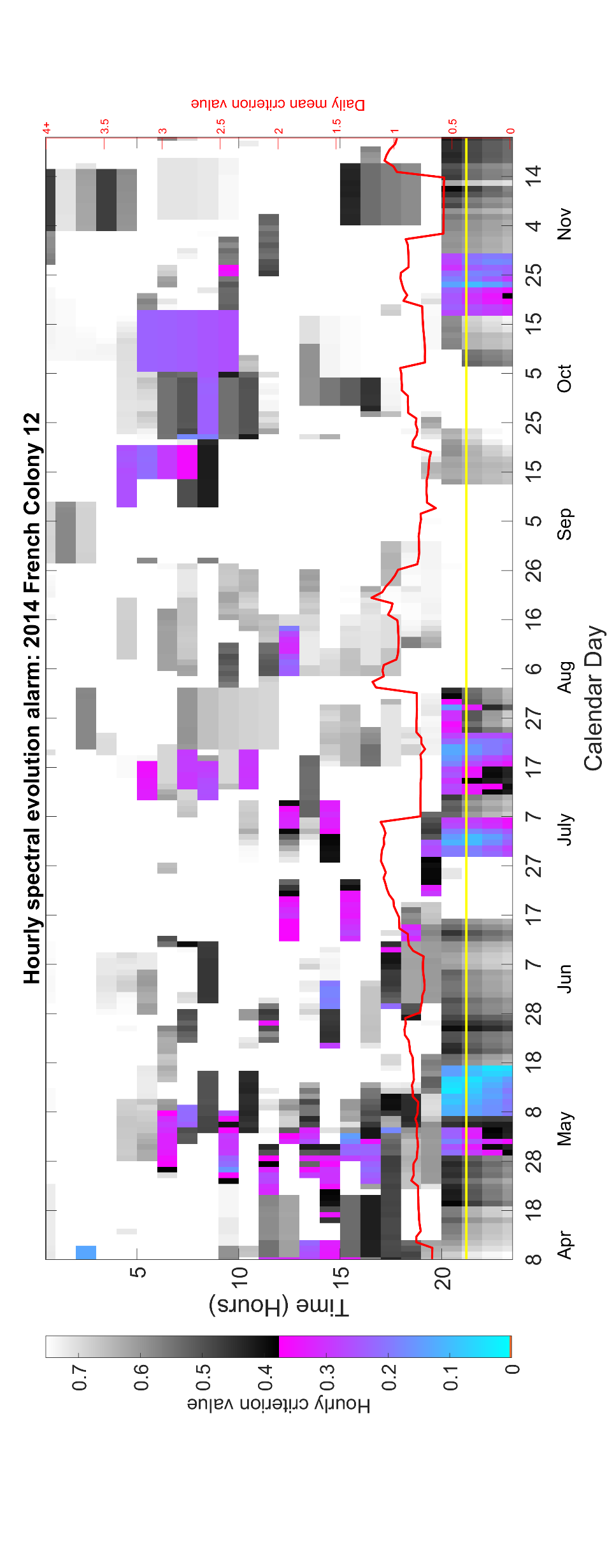

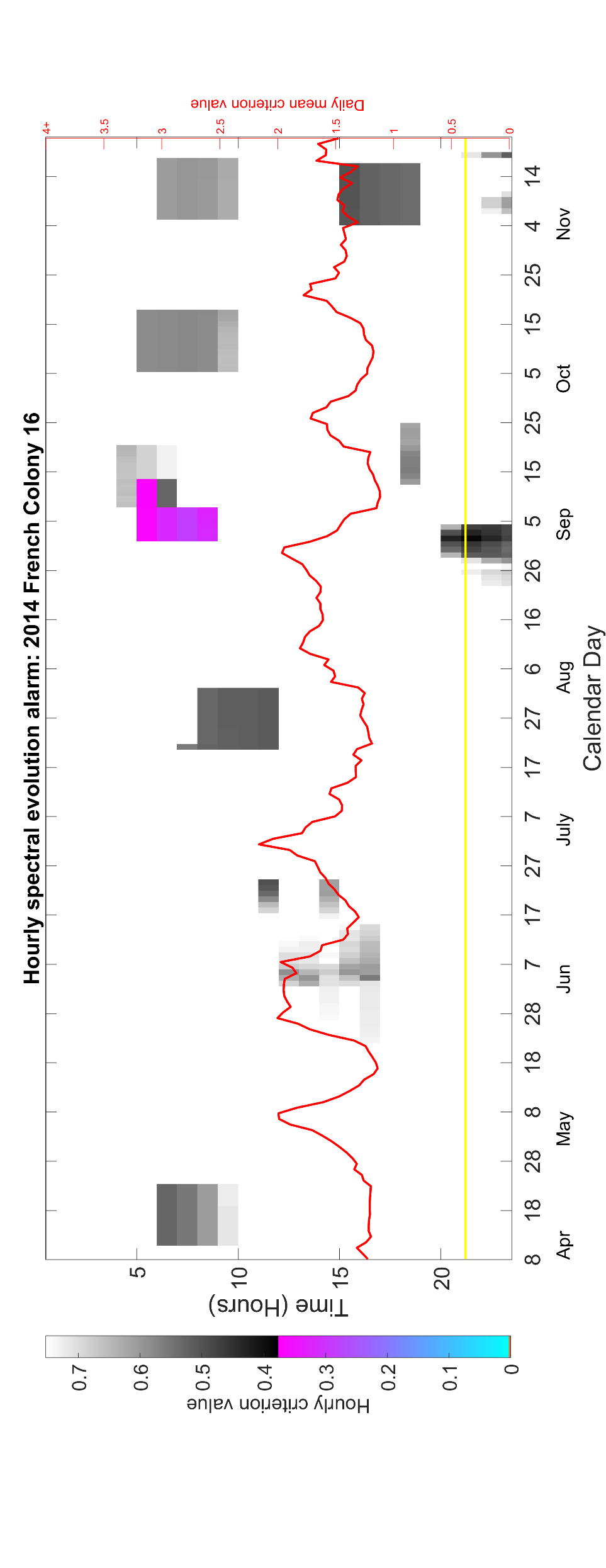

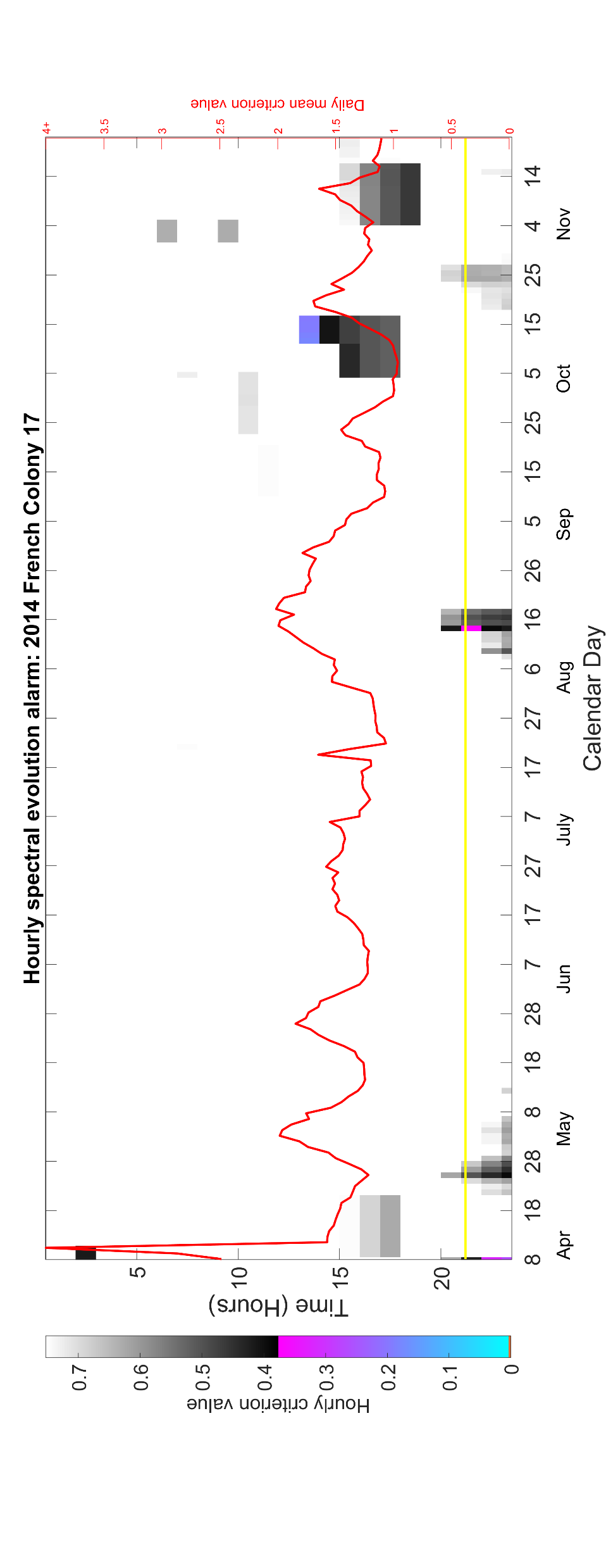

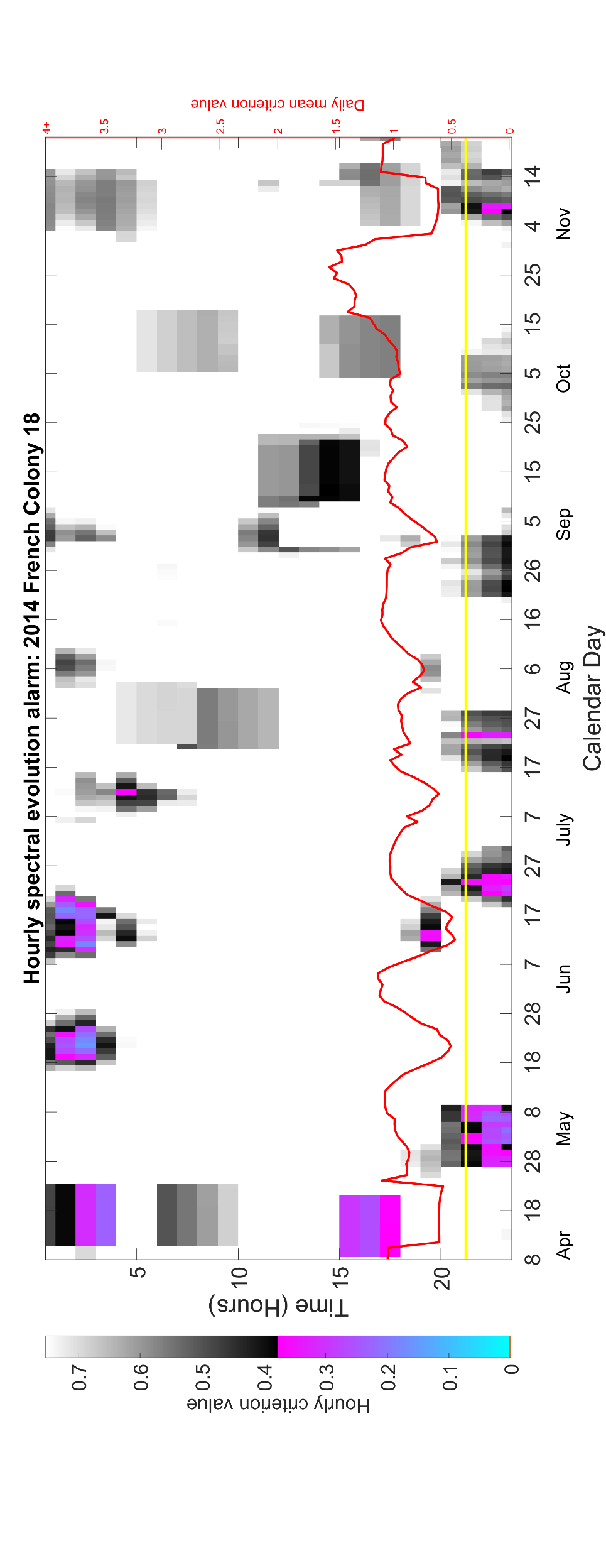

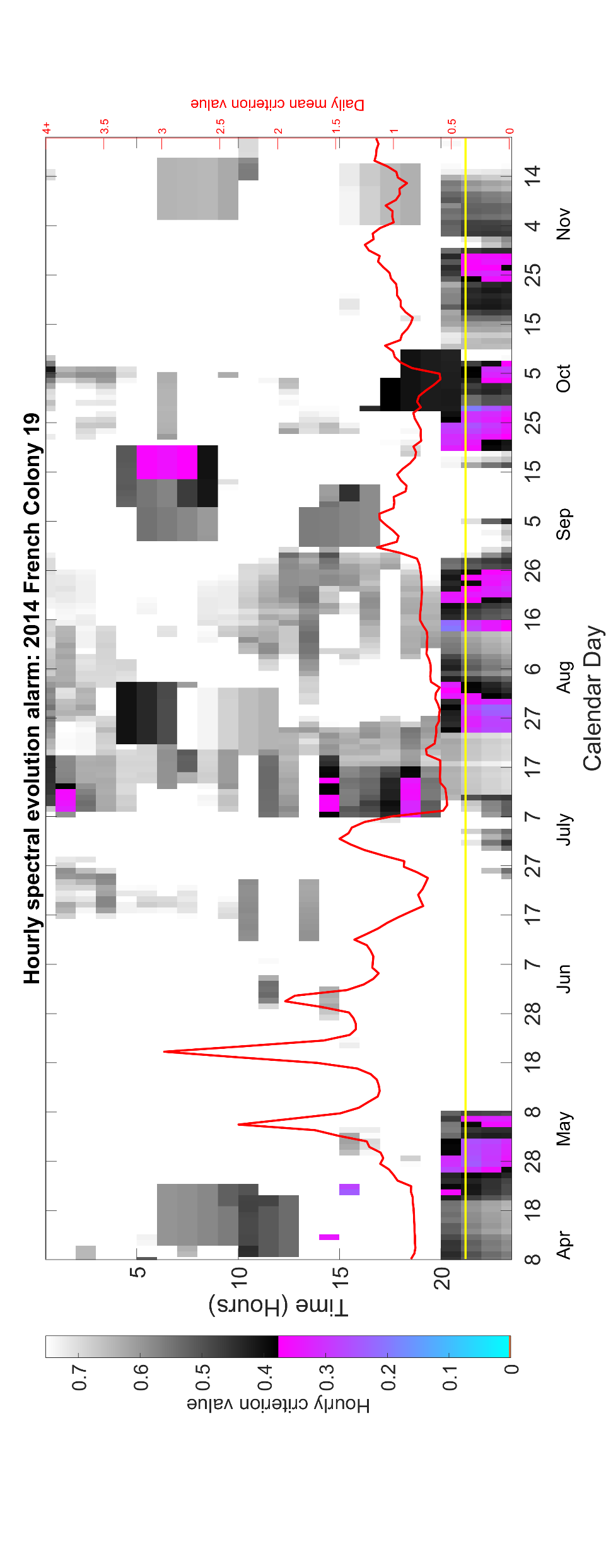

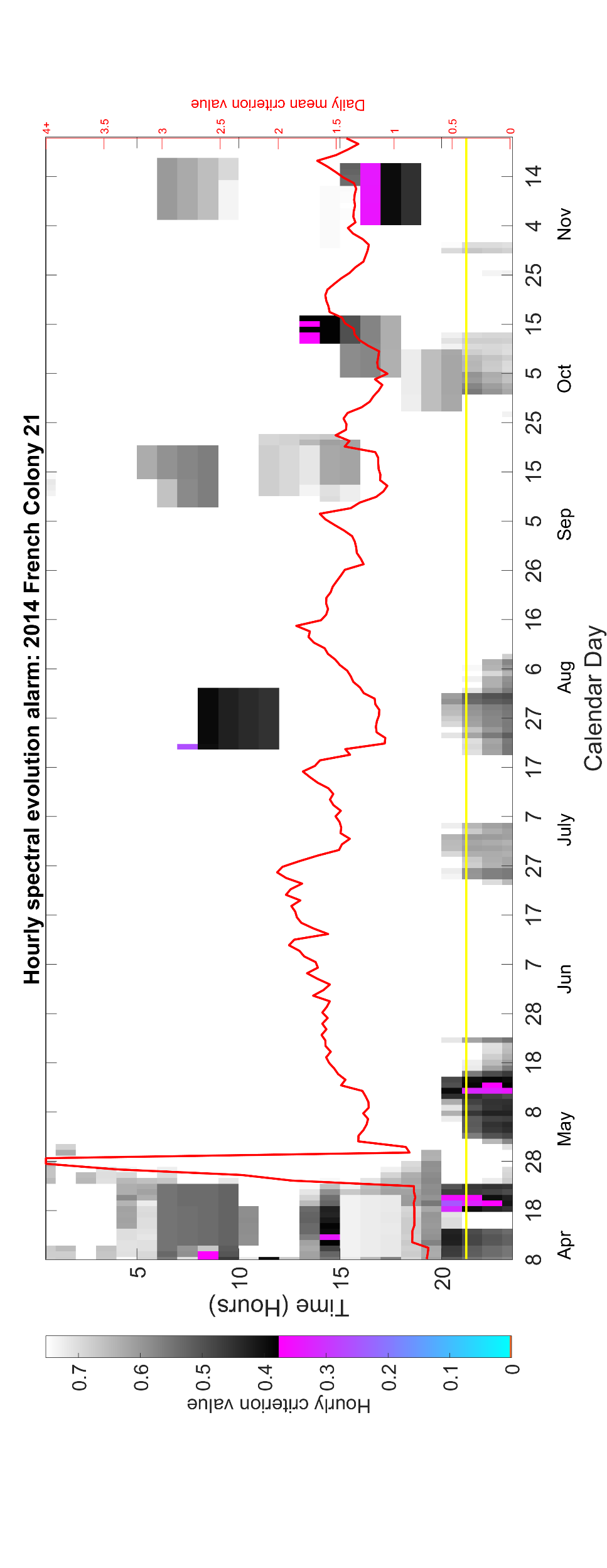

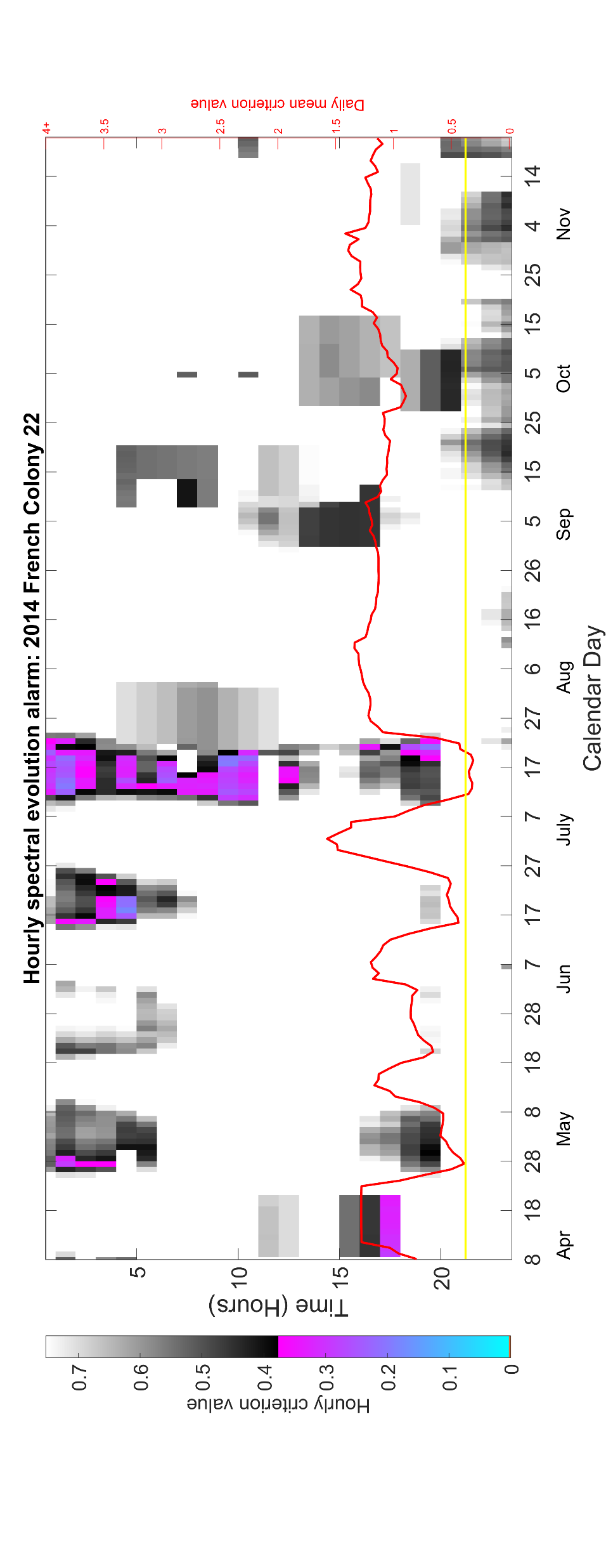


*Fig S34:* ***Non-Swarming Colony.***

***Spectral evolution alarm for 2014 French colony 2,*** *which was monitored from the 8^th^ April until the recording ceased on the 20^th^ November 2014. The colour coding has been split: greyscale colours denote the alarm values above the threshold (non-swarming state) and those in colour denote the alarm values below the threshold (swarming state), from pink to blue as the criterion approaches the swarming centroid. Superimposed is another set of axis showing the average of the previous night’s alarm taken between midnight and 5am, with the yellow line displaying the alarm threshold.*

*Fig S35:* ***Non-Swarming Colony.***

***Spectral evolution alarm for 2014 French colony 3,*** *which was monitored from the 8^th^ April until the recording ceased on the 20^th^ November 2014. The features of the plot are identical to that of the Fig S34.*

*Fig S36:* ***Non-Swarming Colony.***

***Spectral evolution alarm for 2014 French colony 4,*** *which was monitored from the 8^th^ April until the recording ceased on the 20^th^ November 2014. The features of the plot are identical to that of the Fig S34.*

*Fig S37:* ***Non-Swarming Colony.***

***Spectral evolution alarm for 2014 French colony 5,*** *which was monitored from the 8^th^ April until the recording ceased on the 20^th^ November 2014. The features of the plot are identical to that of the Fig S34.*

*Fig S38:* ***Non-Swarming Colony.***

***Spectral evolution alarm for 2014 French colony 8,*** *which was monitored from the 8th April until the recording ceased on the 20th November 2014****.*** *The features of the plot are identical to that of the Fig S34.*

*Fig S39:* ***Non-Swarming Colony.***

***Spectral evolution alarm for 2014 French colony 9,*** *which was monitored from the 8^th^ April until the recording ceased on the 20^th^ November 2014. The features of the plot are identical to that of the Fig S34.*

*Fig S40:* ***Non-Swarming Colony.***

***Spectral evolution alarm for 2014 French colony 11,*** *which was monitored from the 8^th^ April until the recording ceased on the 20^th^ November 2014. The features of the plot are identical to that of the Fig S34.*

*Fig S41:* ***Non-Swarming Colony.***

***Spectral evolution alarm for 2014 French colony 12,*** *which was monitored from the 8^th^ April until the recording ceased on the 20^th^ November 2014. The features of the plot are identical to that of the Fig S34.*

*Fig S42:* ***Non-Swarming Colony.***

***Spectral evolution alarm for 2014 French colony 16,*** *which was monitored from the 8^th^ April until the recording ceased on the 20^th^ November 2014. The features of the plot are identical to that of the Fig S34.*

*Fig S43:* ***Non-Swarming Colony.***

***Spectral evolution alarm for 2014 French colony 17,*** *which was monitored from the 8th April until the recording ceased on the 20^th^ November 2014. The features of the plot are identical to that of the Fig S34.*

*Fig S44:* ***Non-Swarming Colony.***

***Spectral evolution alarm for 2014 French colony 18,*** *which was monitored from the 8th April until the recording ceased on the 20th November 2014The features of the plot are identical to that of the Fig S34.*

*Fig S45:* ***Non-Swarming Colony.***

***Spectral evolution alarm for 2014 INRA colony 19*** *which was monitored from the 8^th^ April until the recording ceased on the 20^th^ November 2014. The features of the plot are identical to that of the Fig S34.*

*Fig S46:* ***Non-Swarming Colony.***

***Spectral evolution alarm for 2014 French colony 21,*** *which was monitored from the 8^th^ April until the recording ceased on the 20^th^ November 2014. The features of the plot are identical to that of the Fig S34.*

*Fig S47:* ***Non-Swarming Colony.***

***Spectral evolution alarm for 2014 French colony 22,*** *which was monitored from the 8^th^ April until the recording ceased on the 20^th^ November 2014. The features of the plot are identical to that of the Fig S34.*

**Spectral Evolution alarm for colonies that swarmed**

In this section, Fig S48 – S64 show the alarm based on the evolution of spectra for various colonies monitored in the UK and France across the 2014 and 2015 active seasons. The plots have been grouped together because they represent colonies that **swarmed**.


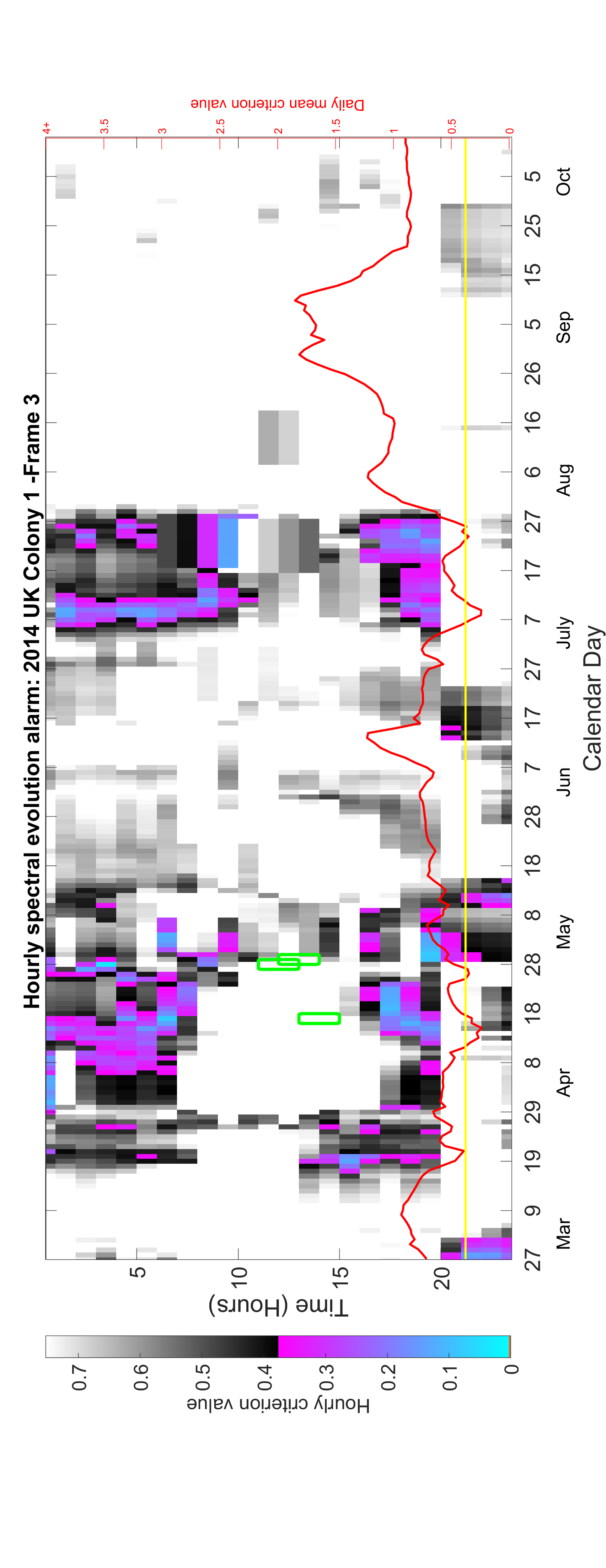

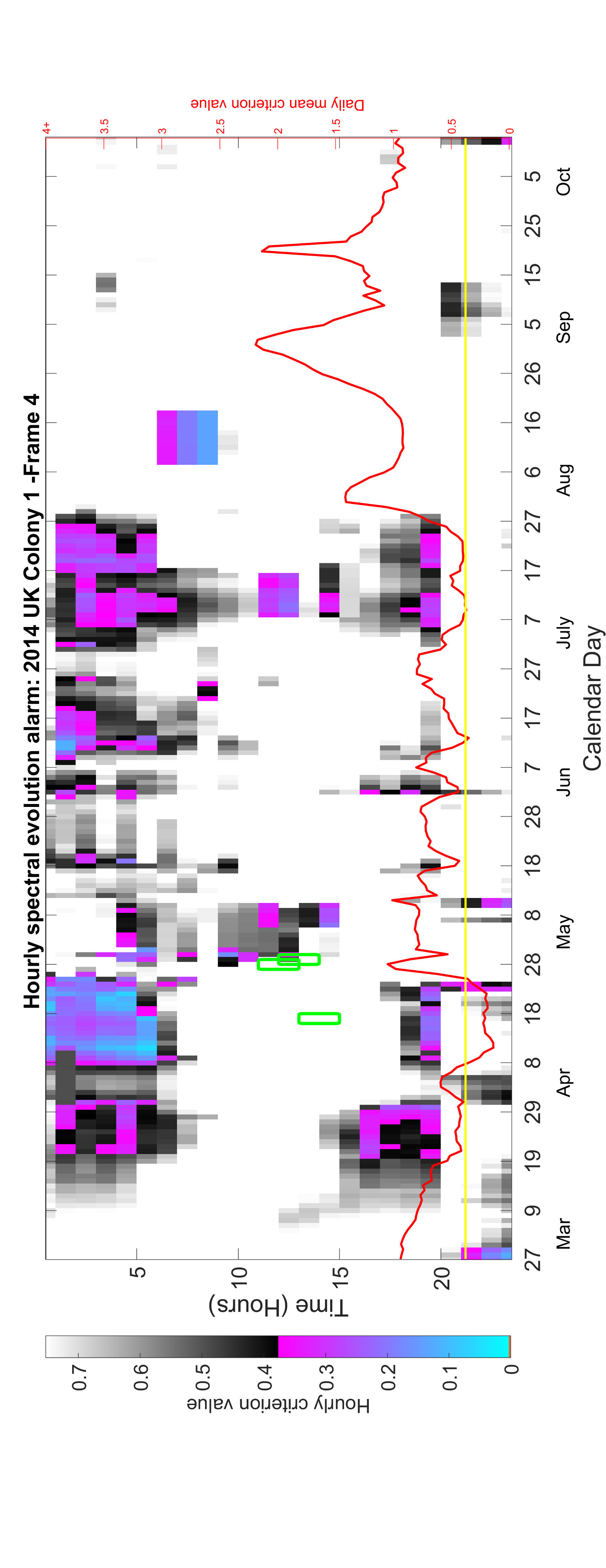

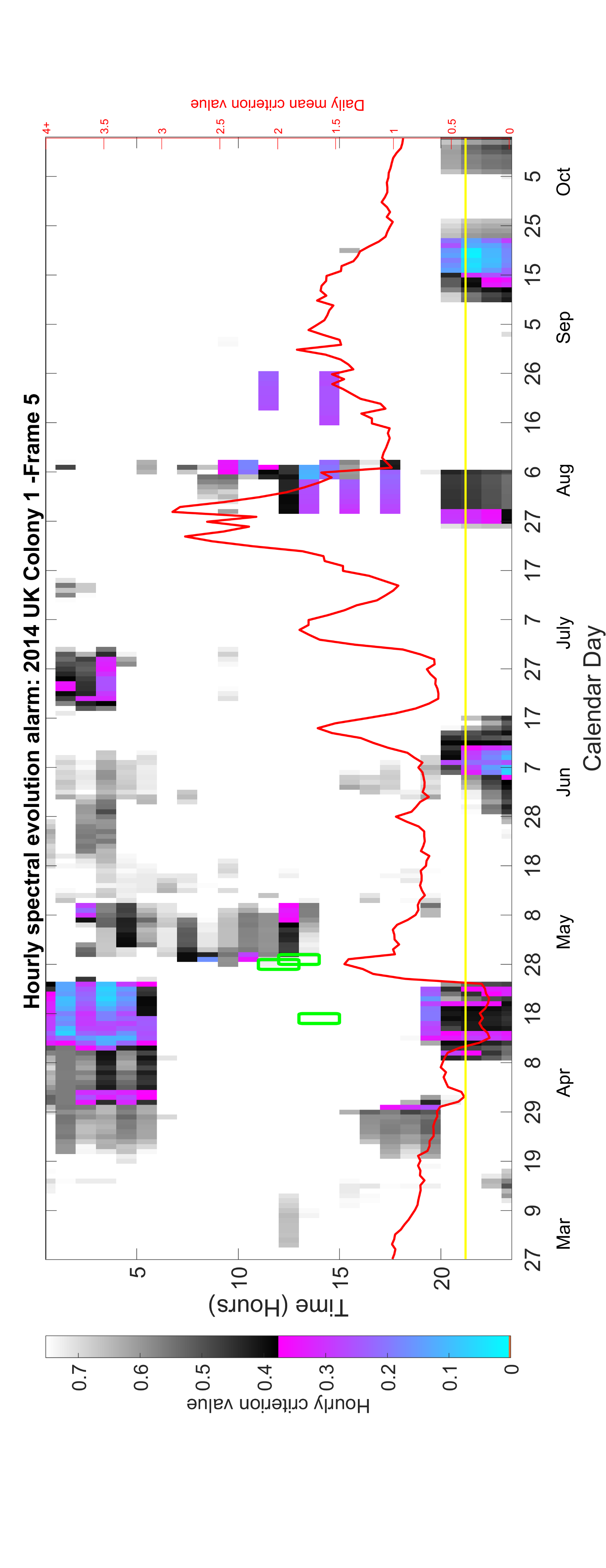

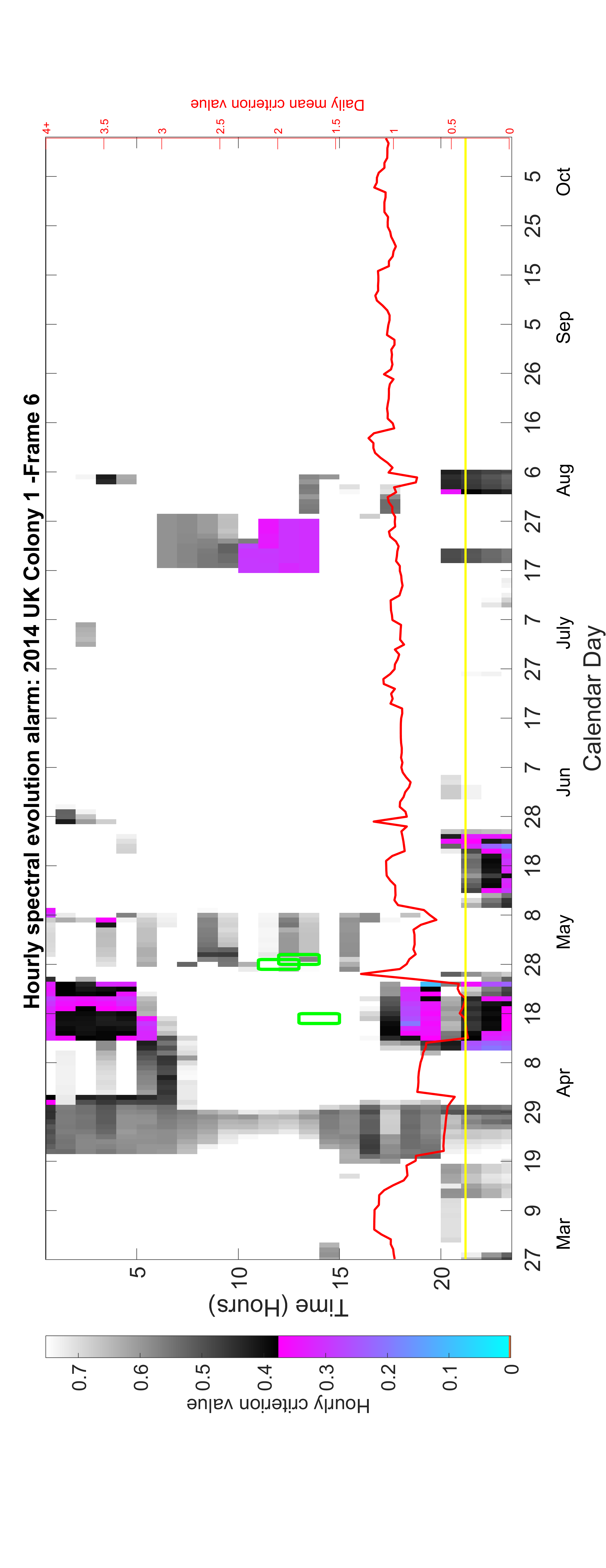

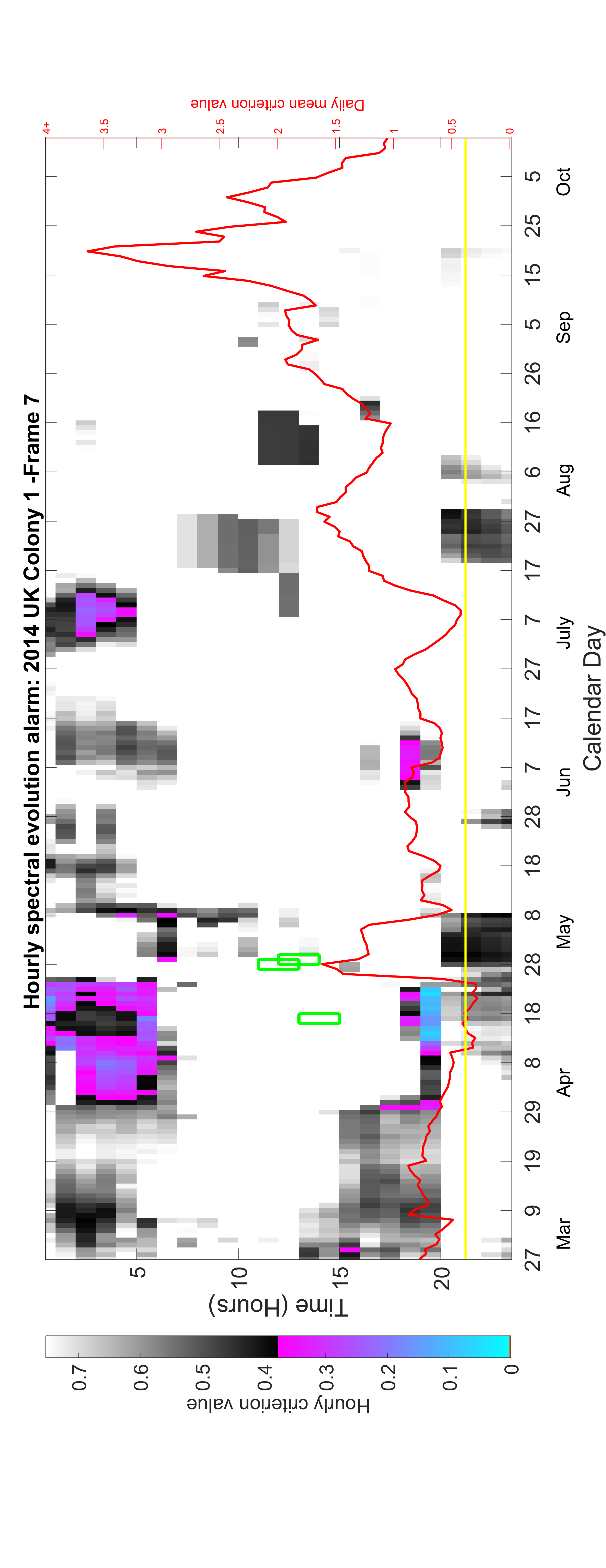

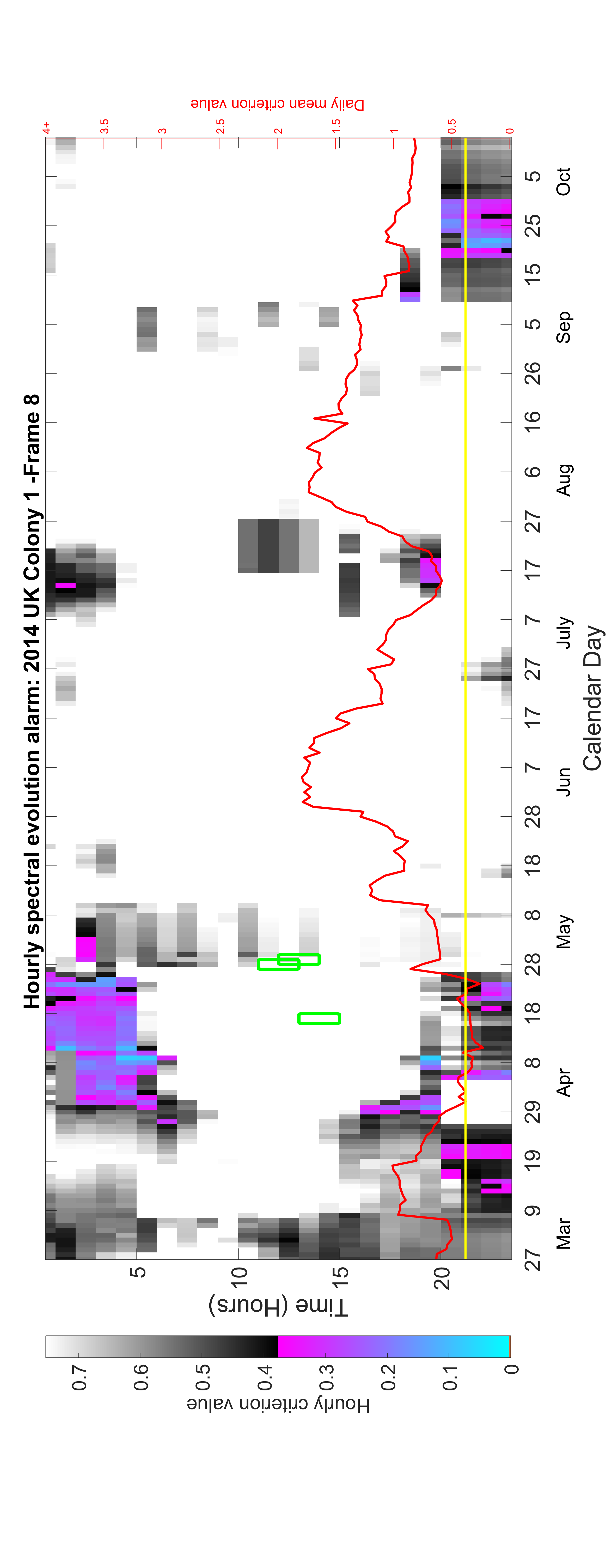

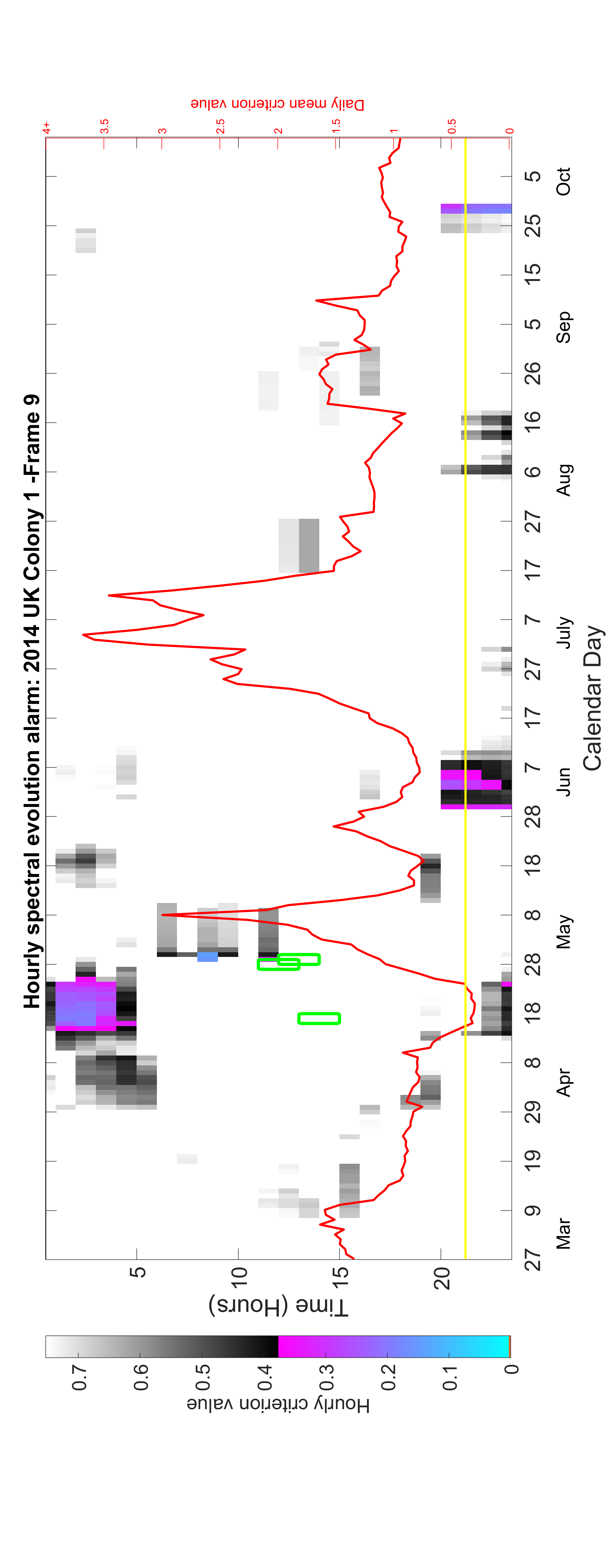

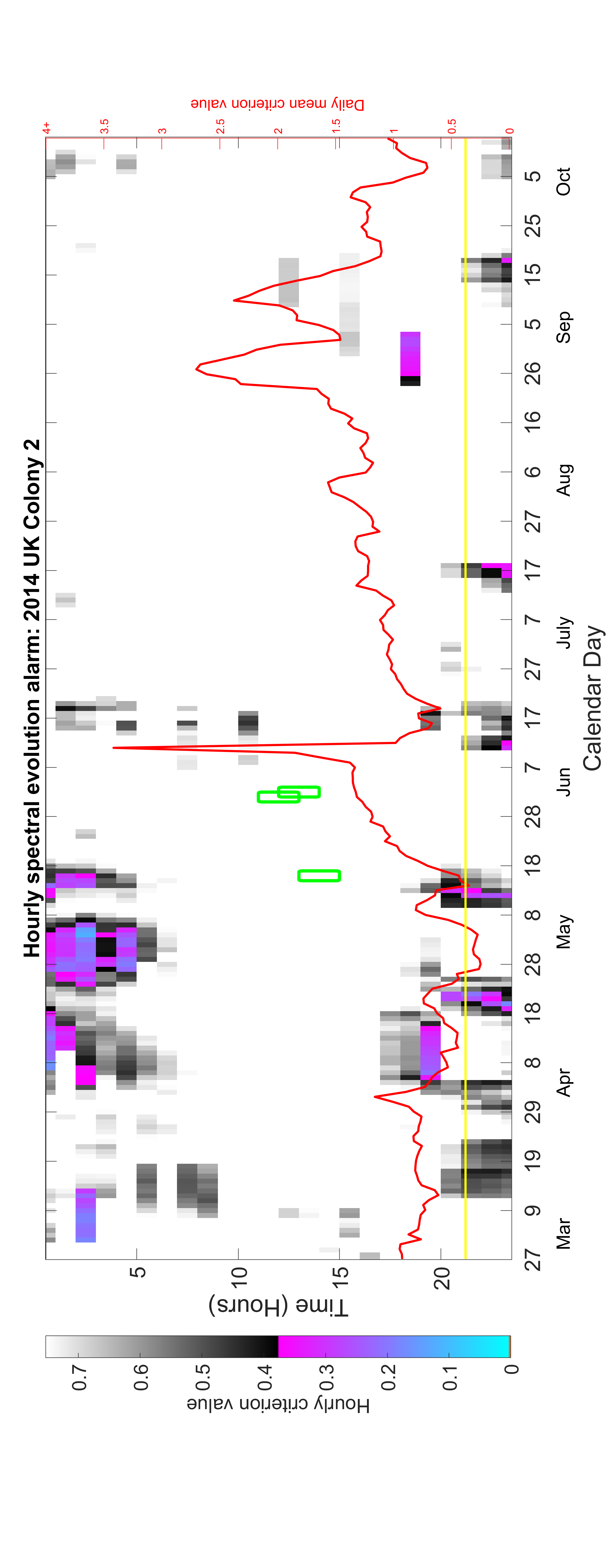


*Fig S48:* ***Swarming Colony.***

*Swarming alarm based on the 10-day evolution of spectra for Frame 3 of the 2014 UK colony 1 based at Nottingham Trent University, shown from March until October. The colour coding has been split. Greyscale colours denote the alarm values above the threshold (non-swarming state) and those in colour denote the alarm values below the threshold (swarming state), from pink to blue as the criterion approaches the swarming centroid. Green rectangles display the timings of three swarms that occurred within this dataset. Superimposed is another set of axis showing the average of the previous night’s alarm computed from data between 9pm and 1am, with the yellow line displaying the alarm threshold.*

*Fig S49:* ***Swarming Colony.***

*Swarming alarm based on the 10-day evolution of spectra for Frame 4 of the 2014 UK colony 1 based at Nottingham Trent University, shown from March until October. The figure properties are Identical to that of Fig S48.*

*Fig S50:* ***Swarming Colony.***

*Swarming alarm based on the 10-day evolution of spectra for Frame 5 of the 2014 UK colony 1 based at Nottingham Trent University, shown from March until October. The figure properties are Identical to that of Fig S48.*

*Fig S51:* ***Swarming Colony.***

*Swarming alarm based on the 10-day evolution of spectra for Frame 6 of the 2014 UK colony 1 based at Nottingham Trent University, shown from March until October. The figure properties are Identical to that of Fig S48.*

*Fig S52:* ***Swarming Colony.***

*Swarming alarm based on the 10-day evolution of spectra for Frame 7 of the 2014 UK colony 1 based at Nottingham Trent University, shown from March until October. The figure properties are Identical to that of Fig S48.*

*Fig S53:* ***Swarming Colony.***

*Swarming alarm based on the 10-day evolution of spectra for Frame 8 of the 2014 UK colony 1 based at Nottingham Trent University, shown from March until October. The figure properties are Identical to that of Fig S48.*

*Fig S54:* ***Swarming Colony.***

*Swarming alarm based on the 10-day evolution of spectra for Frame 9 of the 2014 UK colony 1 based at Nottingham Trent University, shown from March until October. The figure properties are Identical to that of Fig S48.*

*Fig S55:* ***Swarming Colony.***

*Swarming alarm based on the 10-day evolution of spectra for the 2014 UK colony 2 based at Nottingham Trent University, shown from March until October. The figure properties are Identical to that of Fig S48.*


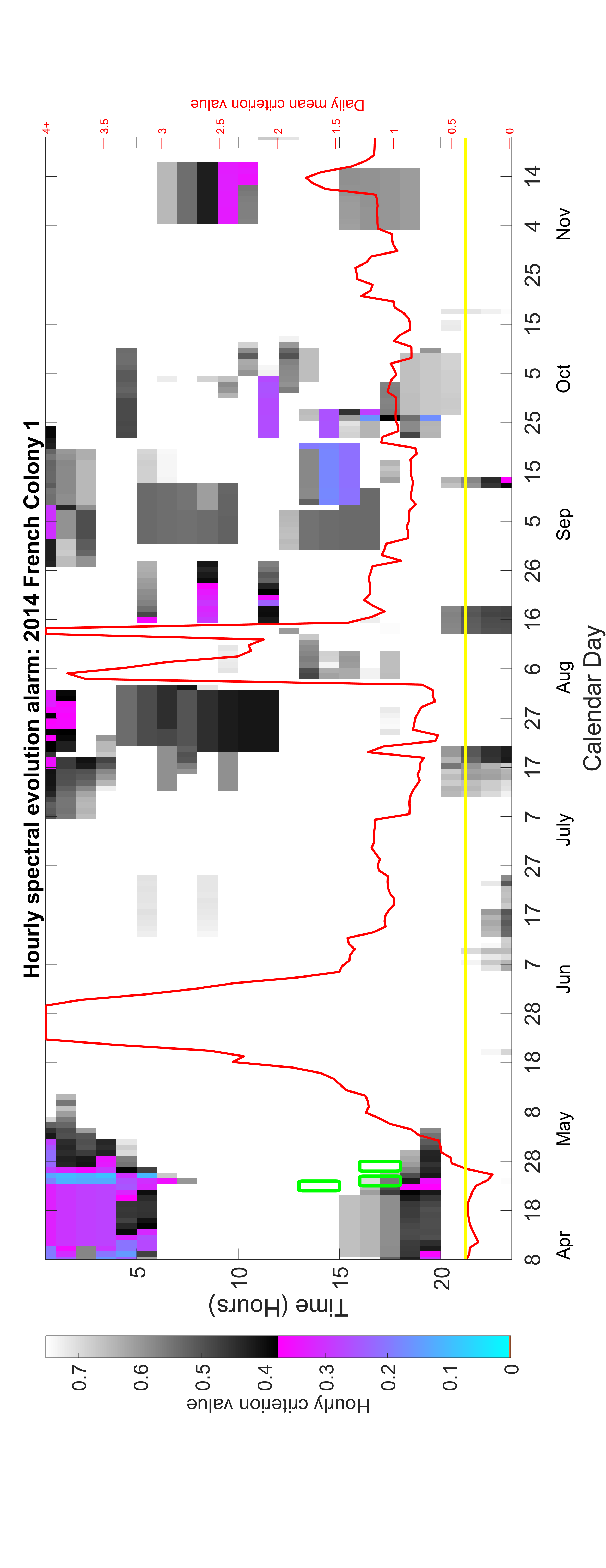


*Fig S56:* ***Swarming Colony.***

*Swarming alarm based on the 10-day evolution of spectra for the French 2014 colony 1, shown from the April until late November. The figure properties are Identical to that of Fig S48.*


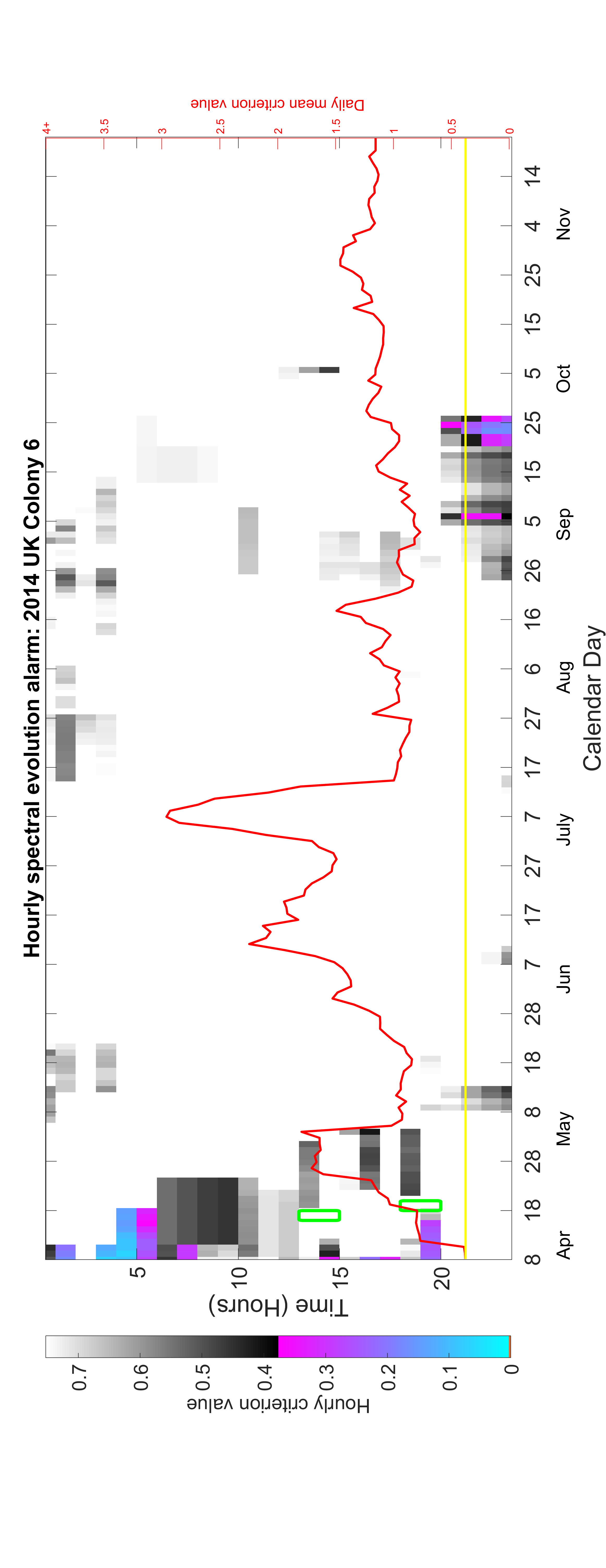

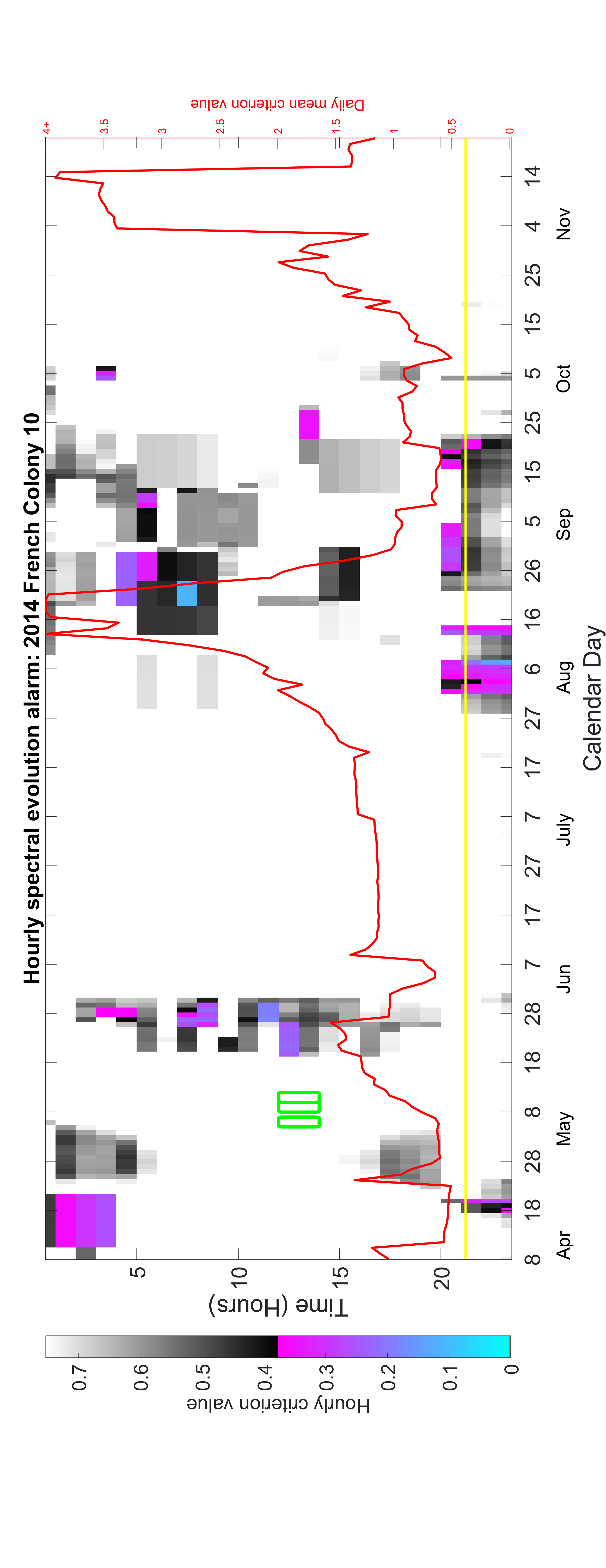

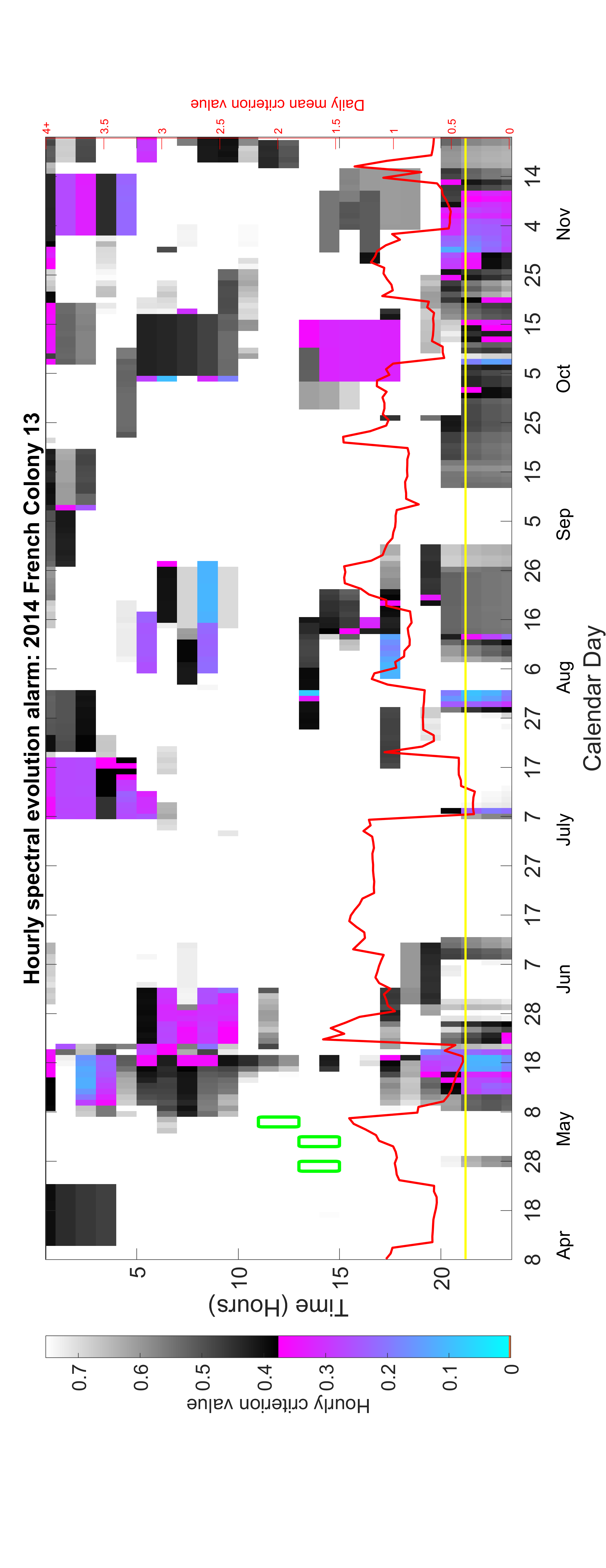

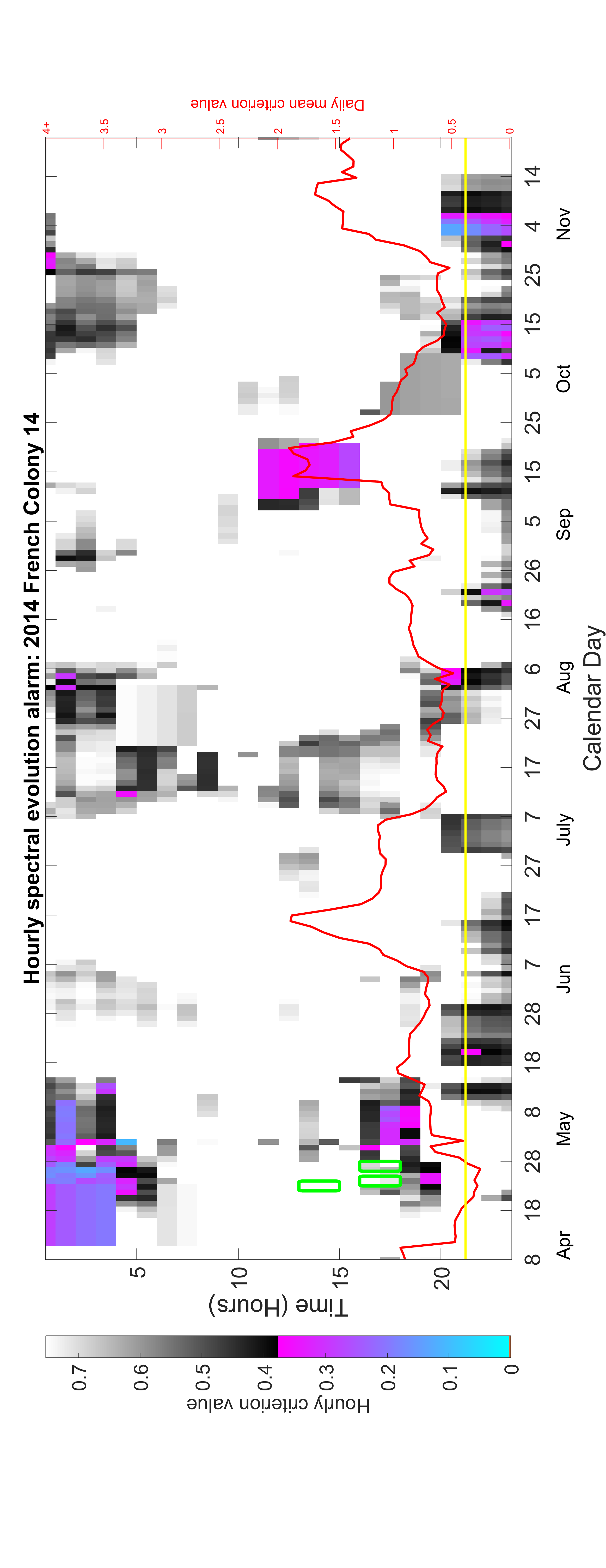

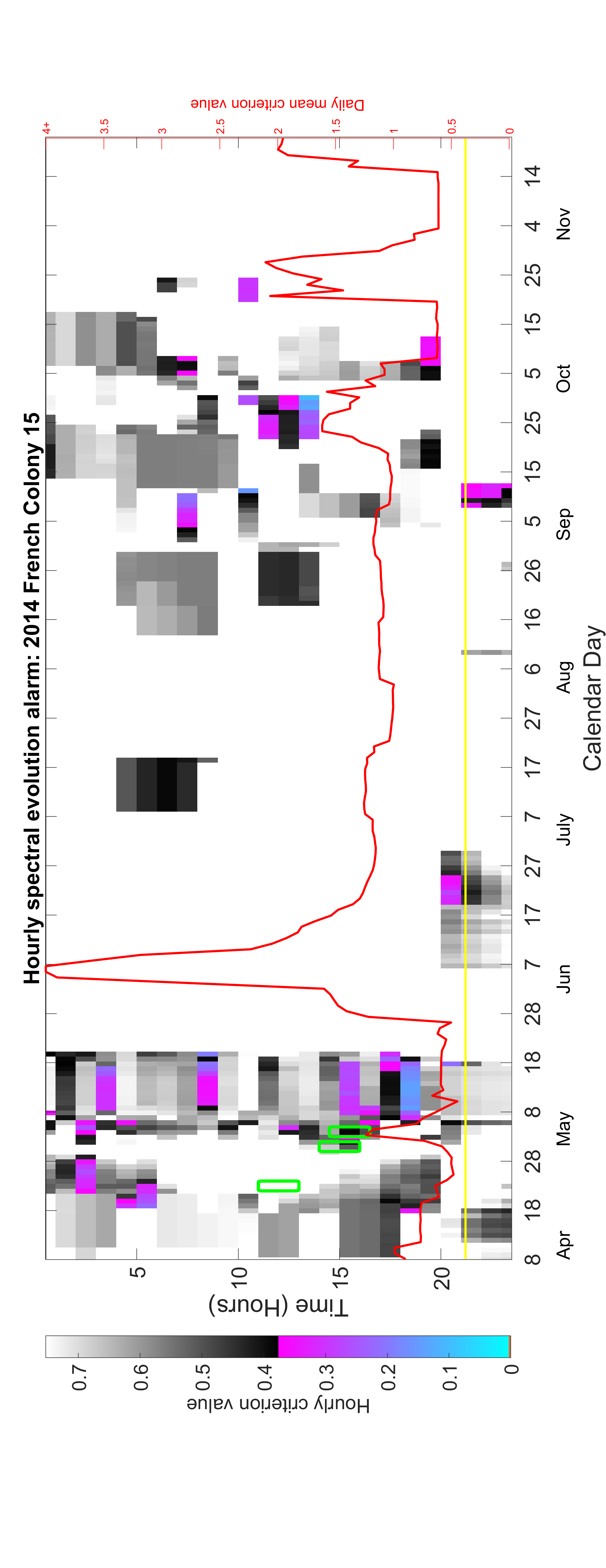


*Fig S57:* ***Swarming Colony.***

*Swarming alarm based on the 10-day evolution of spectra for the French 2014 colony 6, shown from the April until late November. The figure properties are Identical to that of Fig S48.*

*Fig S58:* ***Swarming Colony.***

*Swarming alarm based on the 10-day evolution of spectra for the French 2014 colony 10, shown from the April until late November. The figure properties are Identical to that of Fig S48.*

*Fig S59:* ***Swarming Colony.***

*Swarming alarm based on the 10-day evolution of spectra for the French 2014 colony 13, shown from the April until late November. The figure properties are Identical to that of Fig S48.*

*Fig S60:* ***Swarming Colony.***

*Swarming alarm based on the 10-day evolution of spectra for the French 2014 colony 14, shown from the April until late November. The figure properties are Identical to that of Fig S48.*

*Fig S61:* ***Swarming Colony.***

*Swarming alarm based on the 10-day evolution of spectra for the French 2014 colony 15, shown from the April until late November. The figure properties are Identical to that of Fig S48.*


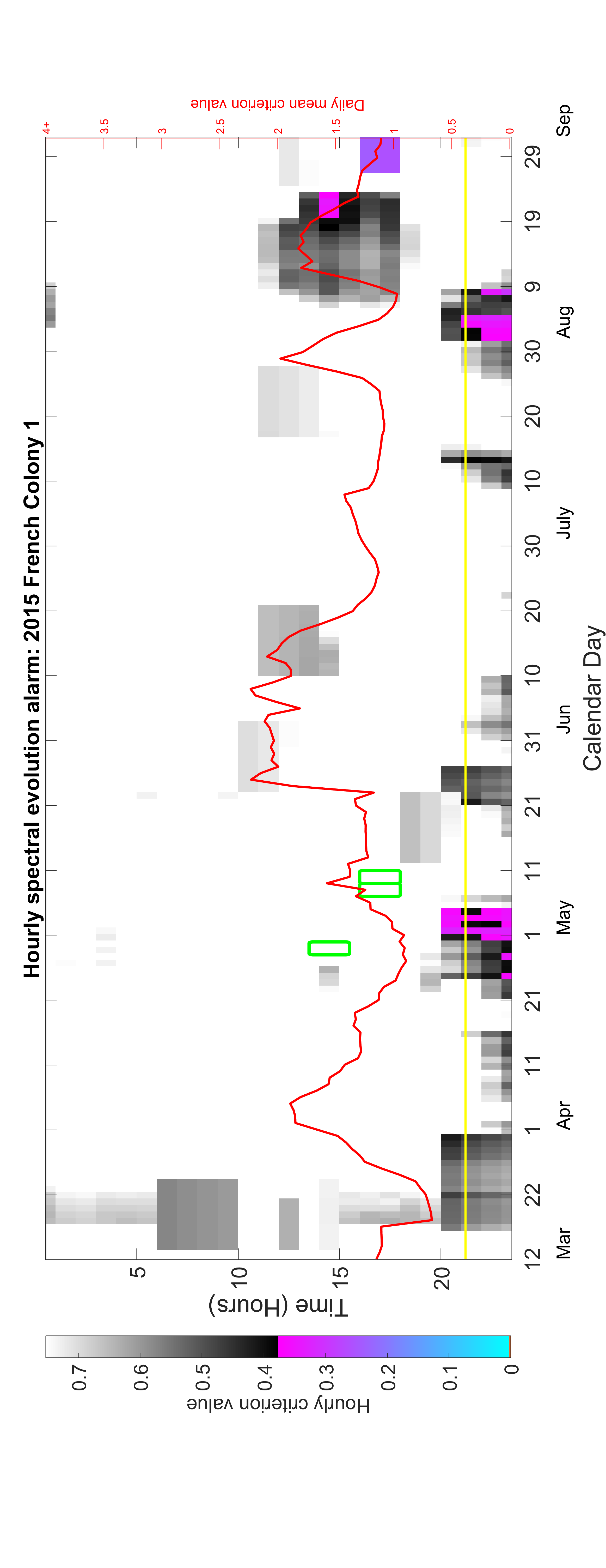

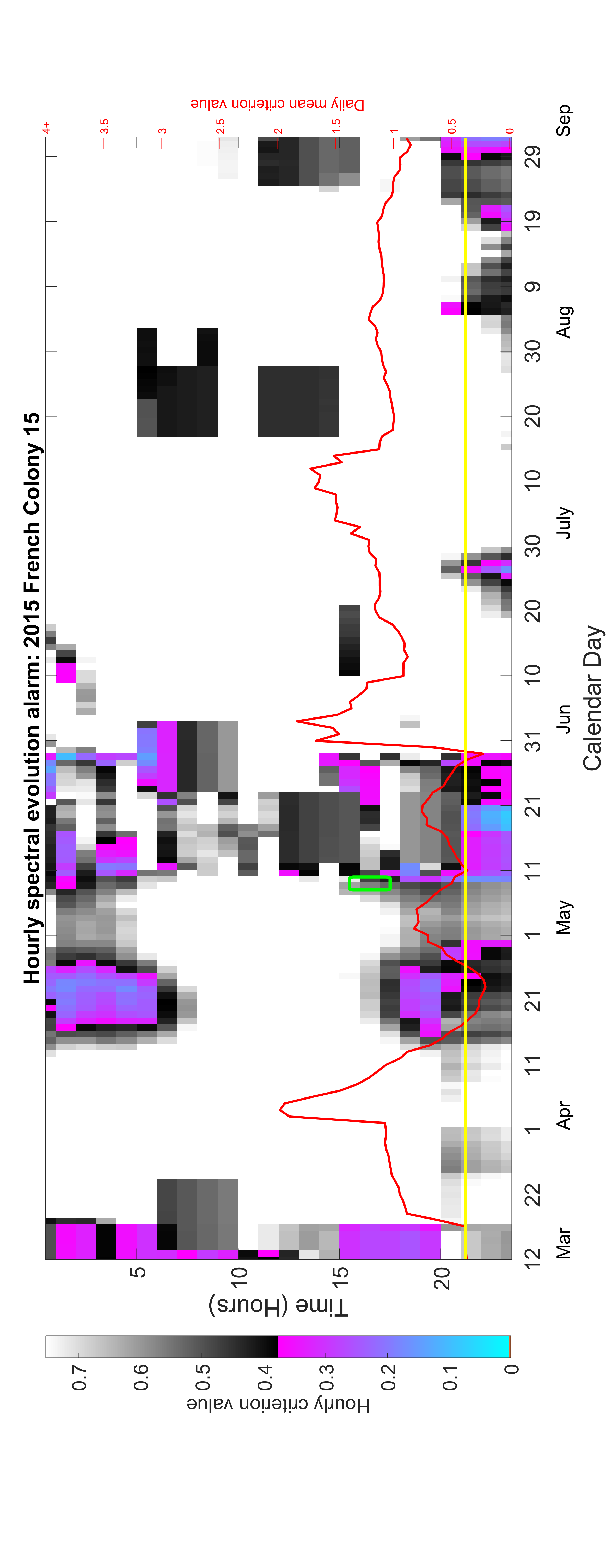

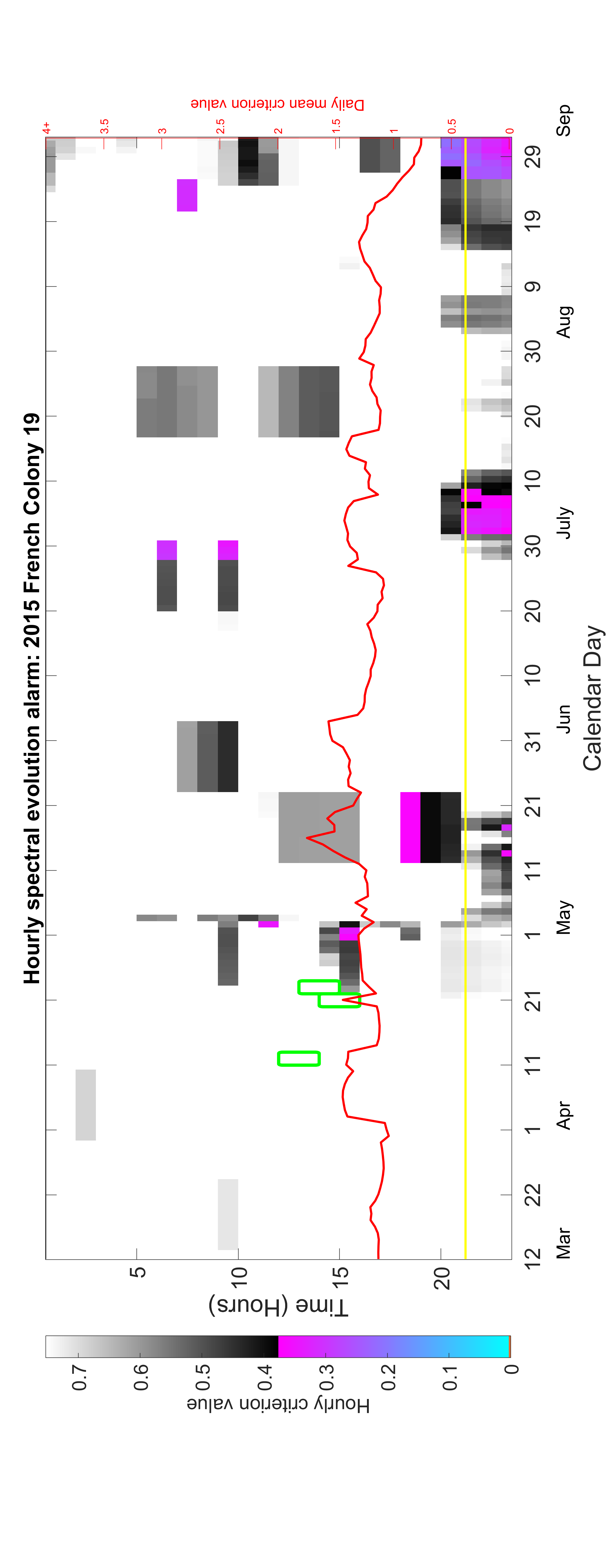


*Fig S62:* ***Swarming Colony.***

*Swarming alarm based on the 10-day evolution of spectra for the French 2015 colony 1, shown from the April until late November. The figure properties are Identical to that of Fig S48.*

*Fig S63:* ***Swarming Colony.***

*Swarming alarm based on the 10-day evolution of spectra for the 2015 colony 15 based at INRA, shown from the April until late November. The figure properties are Identical to that of Fig S48.*

*Fig S64:* ***Swarming Colony.***

*Swarming alarm based on the 10-day evolution of spectra for the 2015 colony B3 based at INRA, shown from the April until late November. The figure properties are Identical to that of Fig S48.*

**
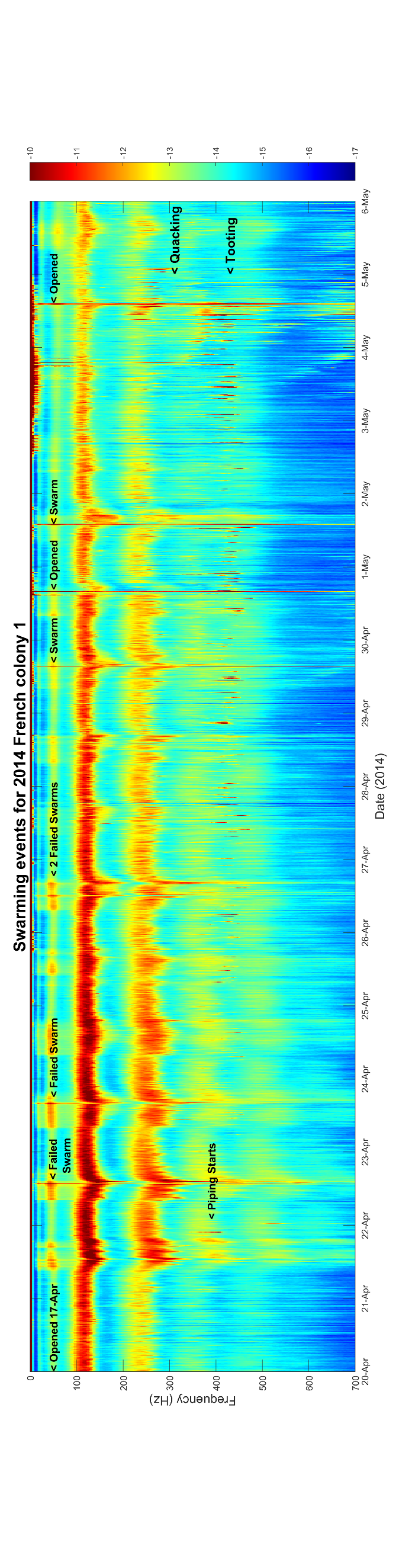

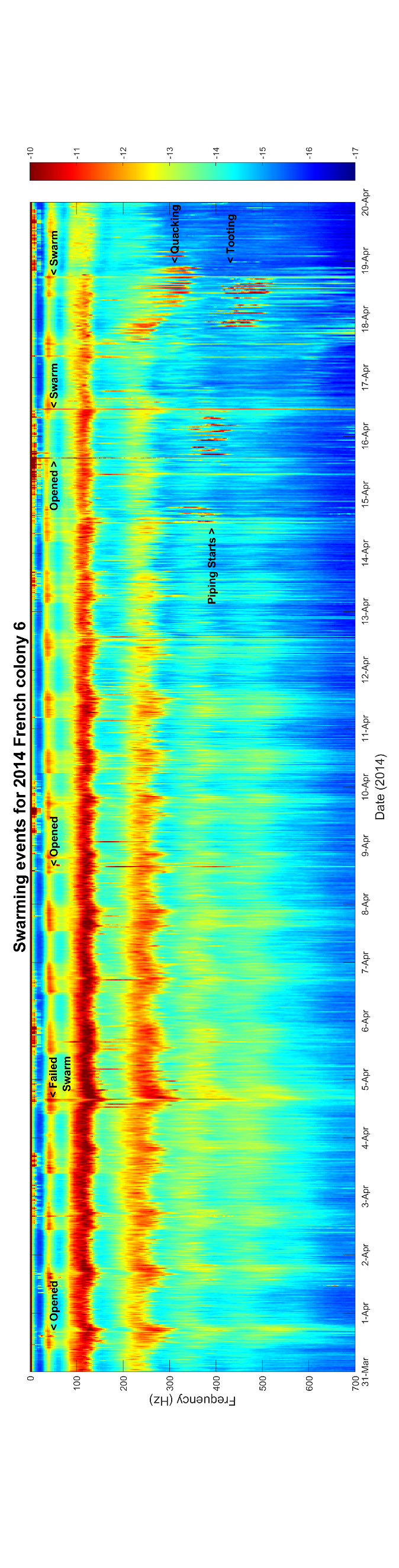

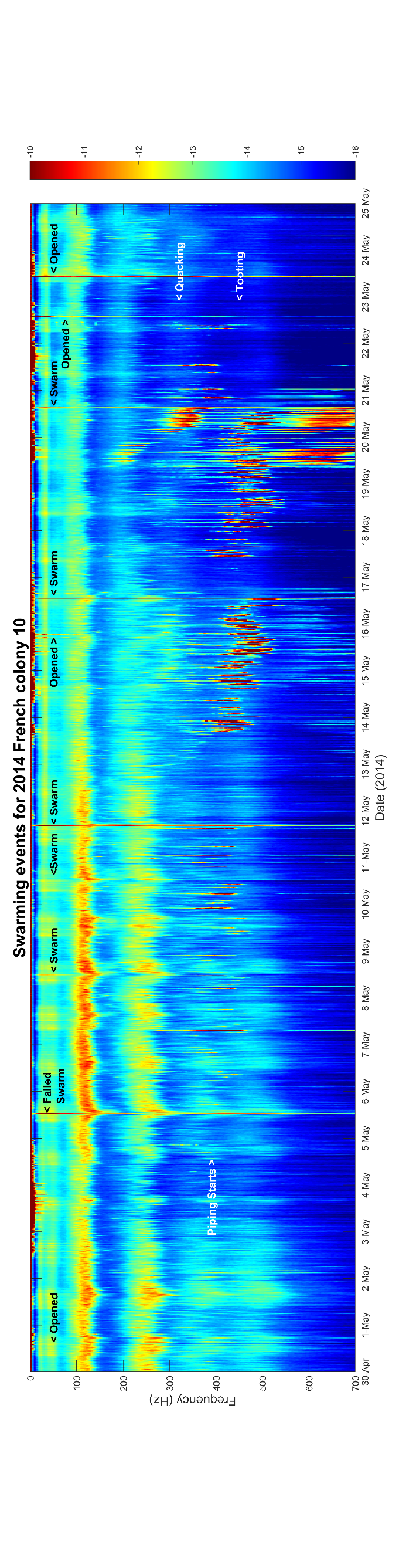

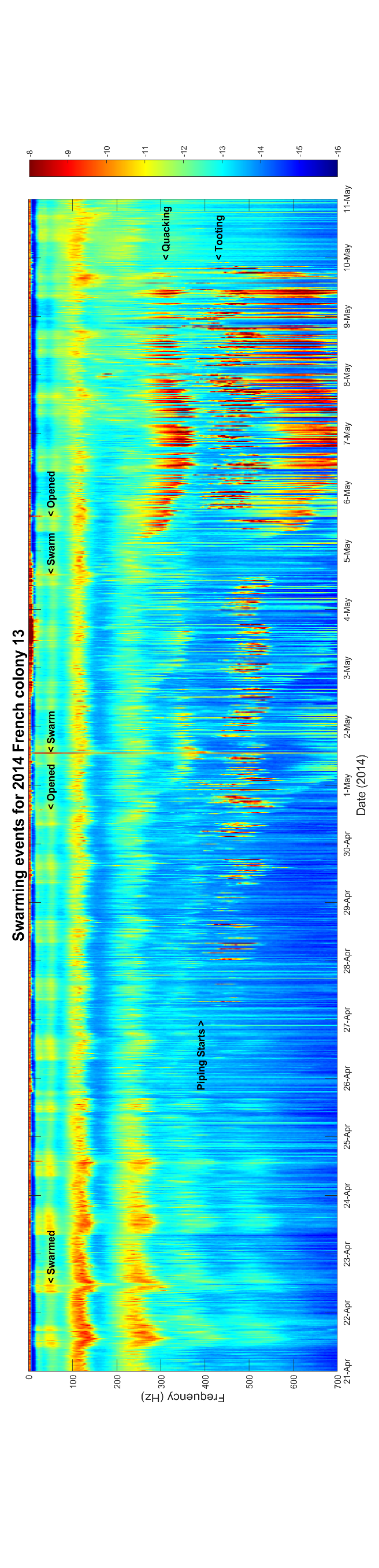

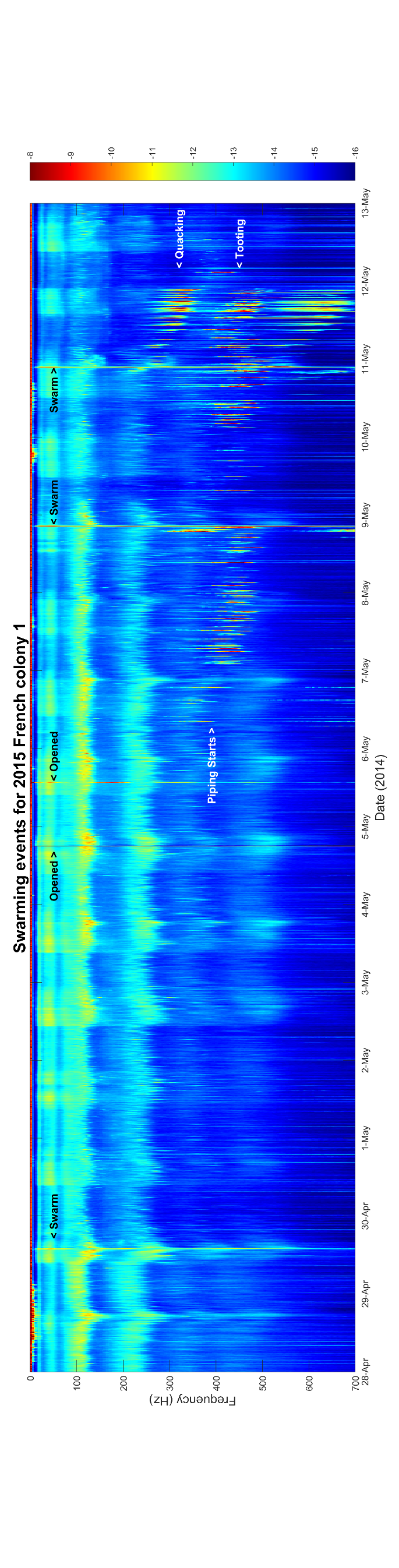
**
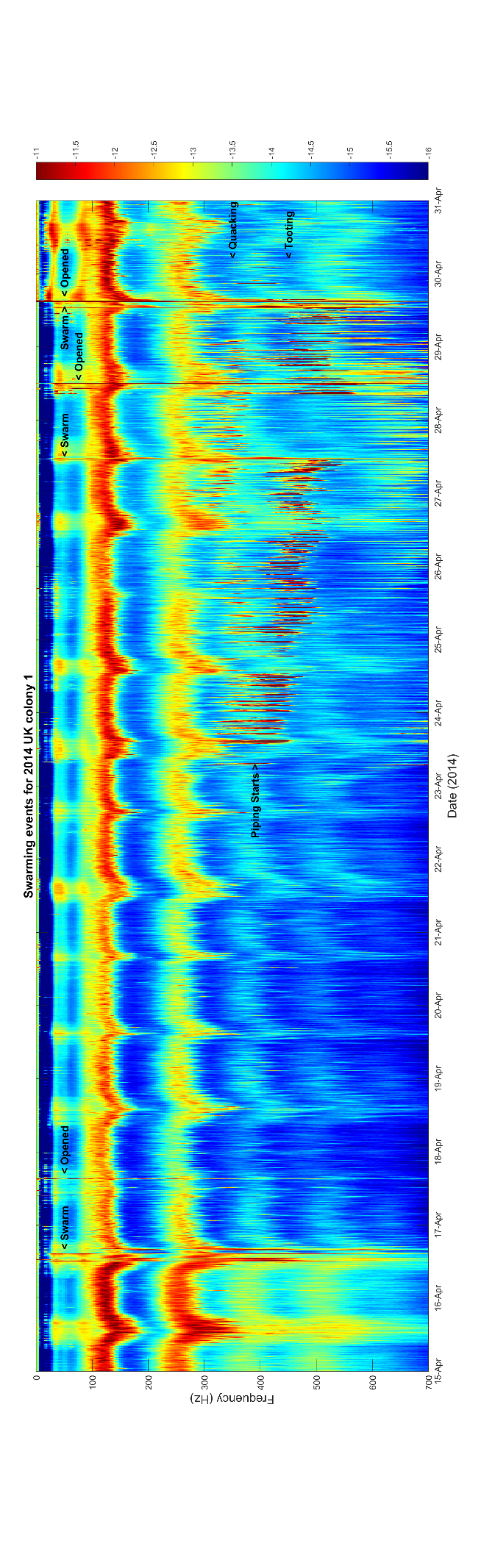

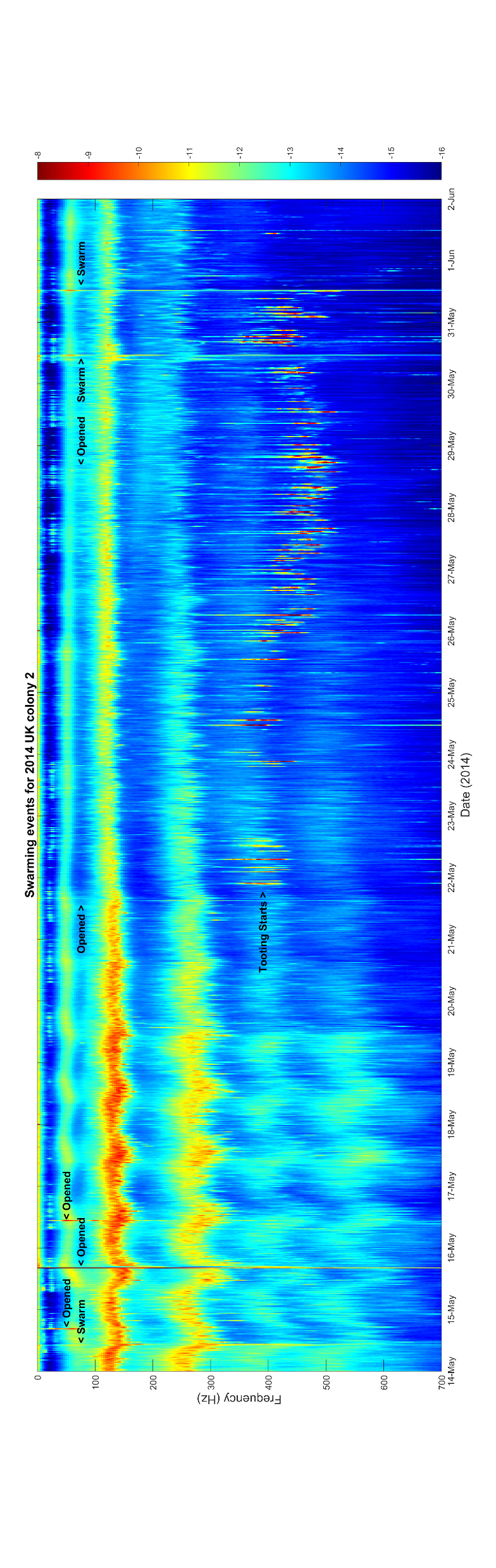

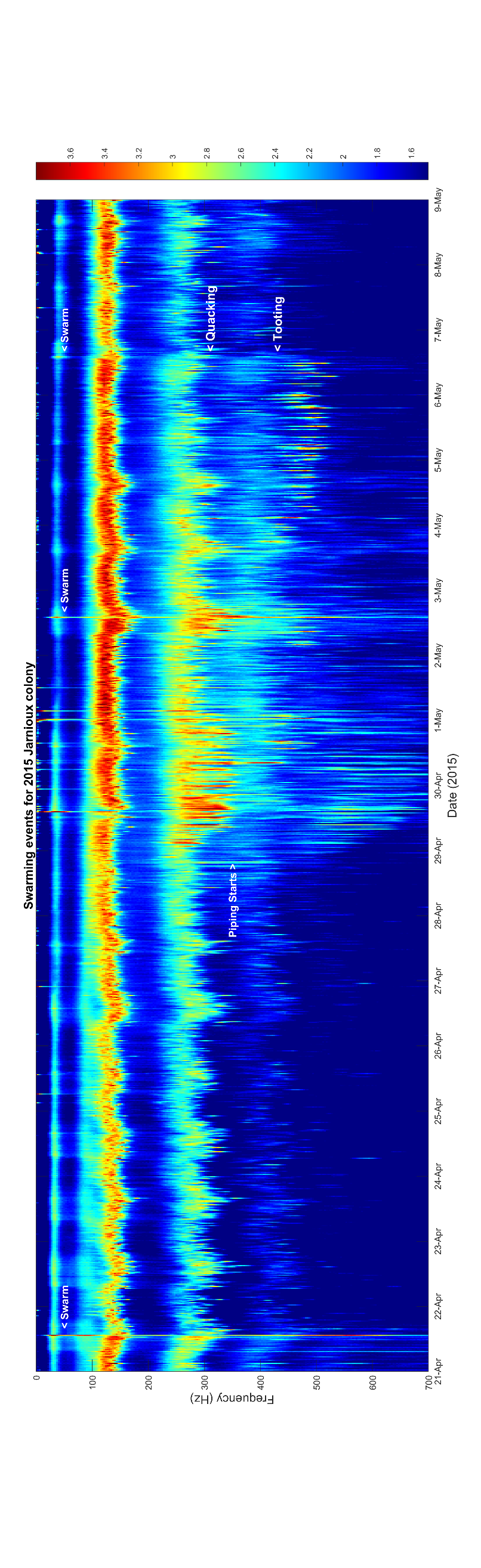


*Fig S65:* ***Spectrogram image of data extracted from the 2014 French colony 1 dataset representing the occurrences of queen piping that occurred within a colony with an original queen that was clipped. This colony was opened several times throughout its swarming activities between 20^th^ April and 6^th^ May 2014.*** Pixel intensity *represents spectral amplitude from low (blue) to high (red).*

*Fig S66:* ***Spectrogram image of data extracted from the 2014 French colony 6 dataset representing the occurrences of queen piping that occurred within a colony with an original queen that was clipped. This colony was opened several times throughout its swarming activities between 31^st^ March and 20^th^ April 2014.*** Pixel intensity *represents spectral amplitude from low (blue) to high (red).*

*Fig S67:* ***Spectrogram image of data extracted from the 2014 French colony 10 dataset representing the occurrences of queen piping that occurred within a colony with an original queen that was clipped. This colony was opened several times throughout its swarming activities between 30^th^ April and 25^th^ May 2014.*** Pixel intensity *represents spectral amplitude from low (blue) to high (red).*

*Fig S68:* ***Spectrogram image of data extracted from the 2014 French colony 13 dataset representing the occurrences of queen piping that occurred within a colony with an original queen that was left unclipped and was left undisturbed throughout its swarming activities between 21^st^ April and 11^th^ May 2014.*** Pixel intensity *represents spectral amplitude from low (blue) to high (red).*

*Fig S69:* ***Spectrogram image of data extracted from the 2015 French colony 1 dataset representing the occurrences of queen piping that occurred within a colony with an original queen that was left unclipped. The colony was opened several times throughout its swarming activities between 28th April and 13^th^ May 2014.*** *Pixel intensity represents spectral amplitude from low (blue) to high (red).*

*Fig S70:* ***Spectrogram image of data extracted from the 2014 UK colony 1 dataset representing the occurrences of queen piping that occurred within a colony with an original queen that was left unclipped. The colony was opened several times throughout its swarming activities between 15^th^ April and 31^st^ April 2014.*** Pixel intensity *represents spectral amplitude from low (blue) to high (red).*

*Fig S71:* ***Spectrogram image of data extracted from the 2014 UK colony 2 dataset representing the occurrences of queen piping that occurred within a colony with an original queen that was unclipped. This colony was opened several times throughout its swarming activities between 14^th^ May and 2^nd^ June 2014.*** Pixel intensity *represents spectral amplitude from low (blue) to high (red).*

*Fig S72:* ***Spectrogram image of data extracted from the 2015 Jarnioux colony dataset representing the occurrences of queen piping that occurred within a colony with an original queen that was left unclipped and was left undisturbed throughout its swarming activities between 21st April and 7^th^ May 2015.*** Pixel intensity *represents spectral amplitude from low (blue) to high (red).*


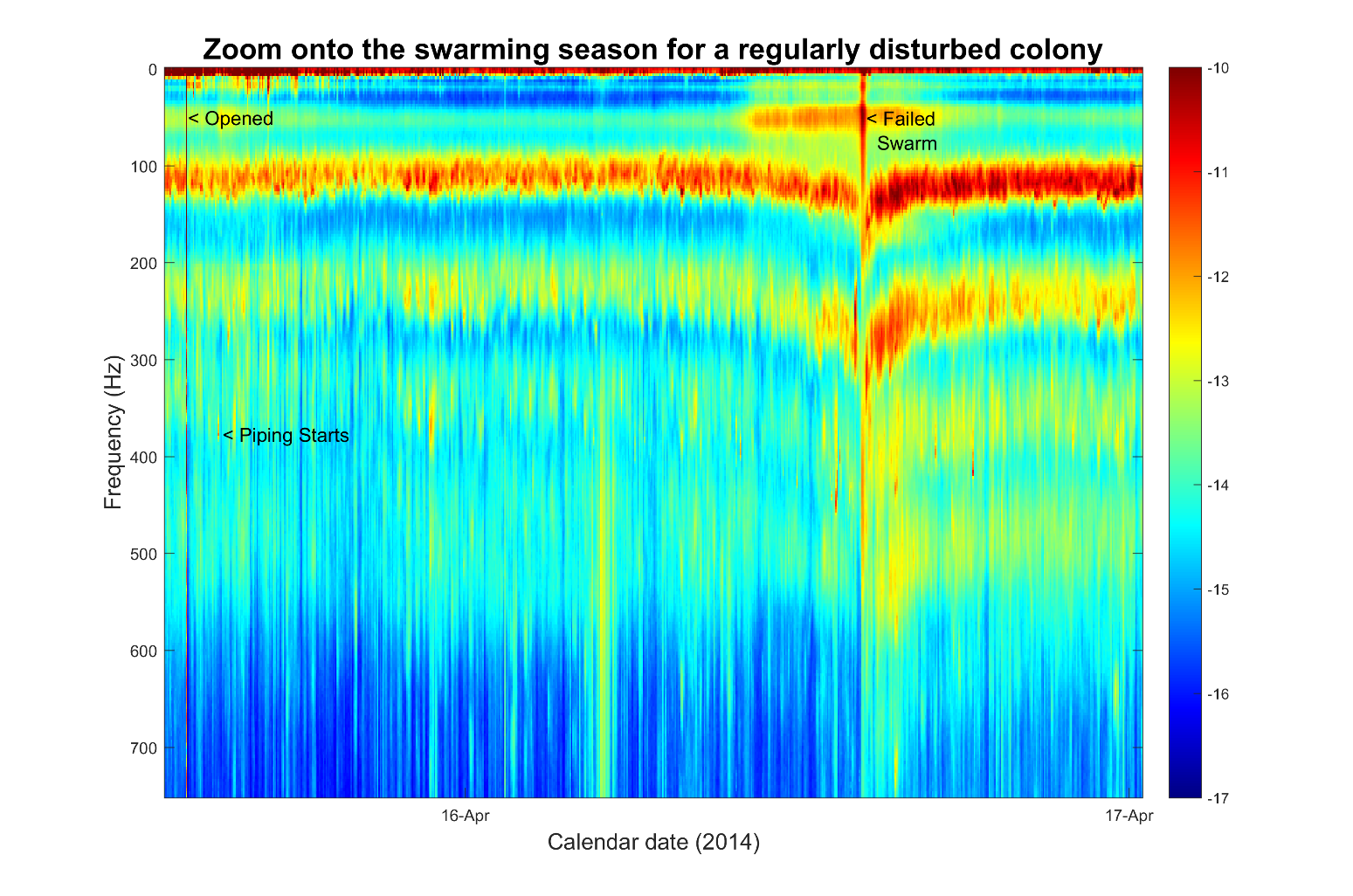


*Fig S73:* ***A further zoom onto the first queen pipe as highlighted in Figure 10.***


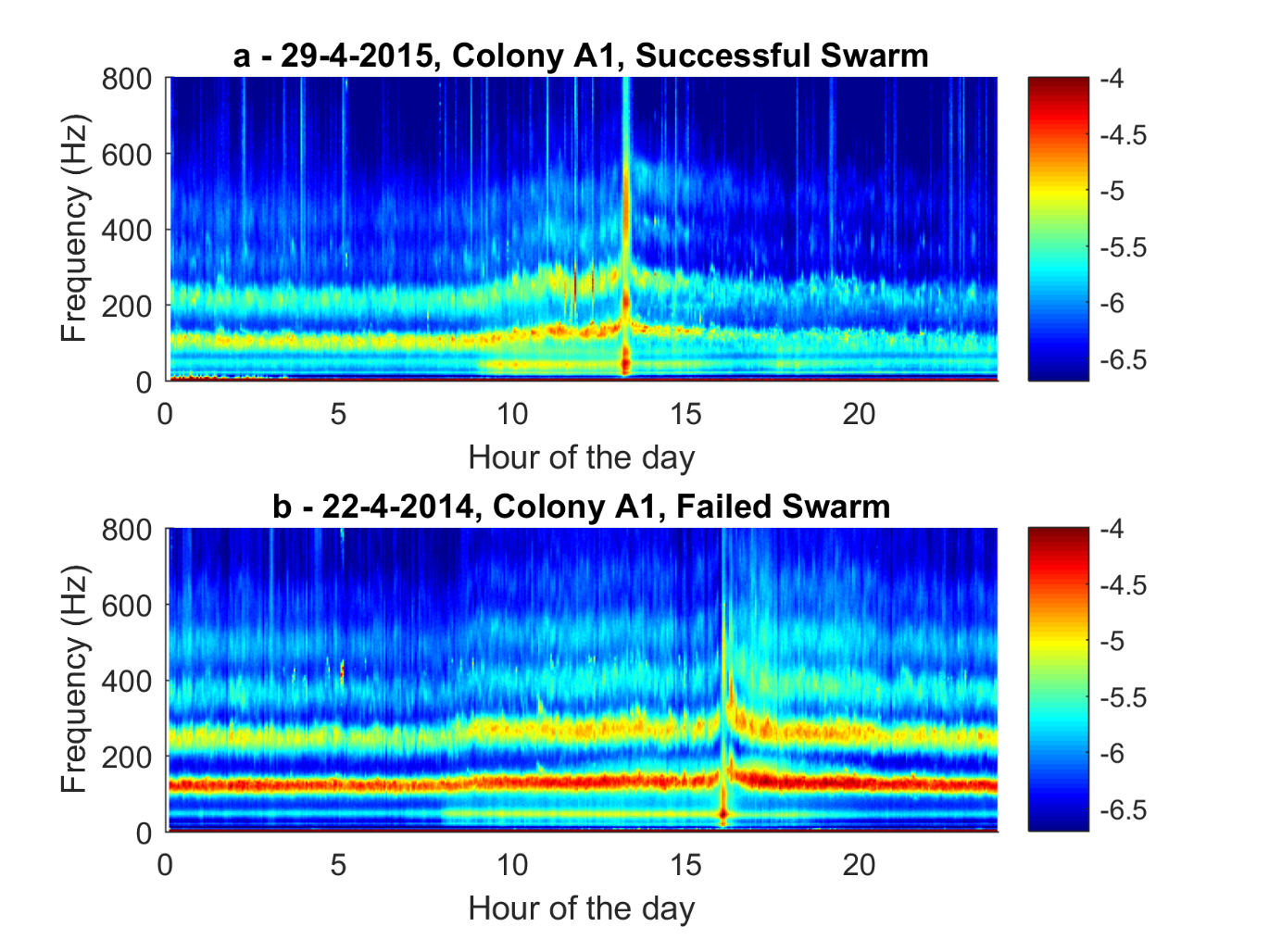


Figure S74: Daily spectrogram of data extracted from a) the 2015 French colony 1 dataset that exemplifies a typical **successful primary swarming event**; and b) ) the 2014 French colony 1 dataset that exemplifies a typical **failed swarming attempt,** as captured within the 3-minute averaged spectrum of vibration.

Although some swarming events are fully corroborated by video evidence from the cameras setup at INRA, such proof is unnecessary as a primary swarm leaves an unmistakable trace on vibrational measurements that are undertaken in this way. This allowed us to locate the exact timings of any primary swarm within the datasets for successful training of the machine learning algorithms. The following features helped to further establish the log of a swarm: (i) they usually take place around lunch time or early afternoon, (ii) they are preceded by a substantial increase in the bee vibrational magnitude and frequency over approximately two to three hours before the lift-off, (iii) they exhibit a huge vibrational magnitude peak at ultra-low frequencies, around 40 Hz, at the time of the lift-off, (iv) they are usually followed by "queen pipes" a few days later, (v) they are followed by a large signal drop, as the queen and worker bees capable of flight leave the hive. If the queen is unclipped (capable of flying) this signal drop will remain for several days, but build up slowly after some days as the young bees mature. However, if the queen was clipped (incapable of flight) there is a large signal enhancement shortly after the drop in signal as the worker bees return to the hive.


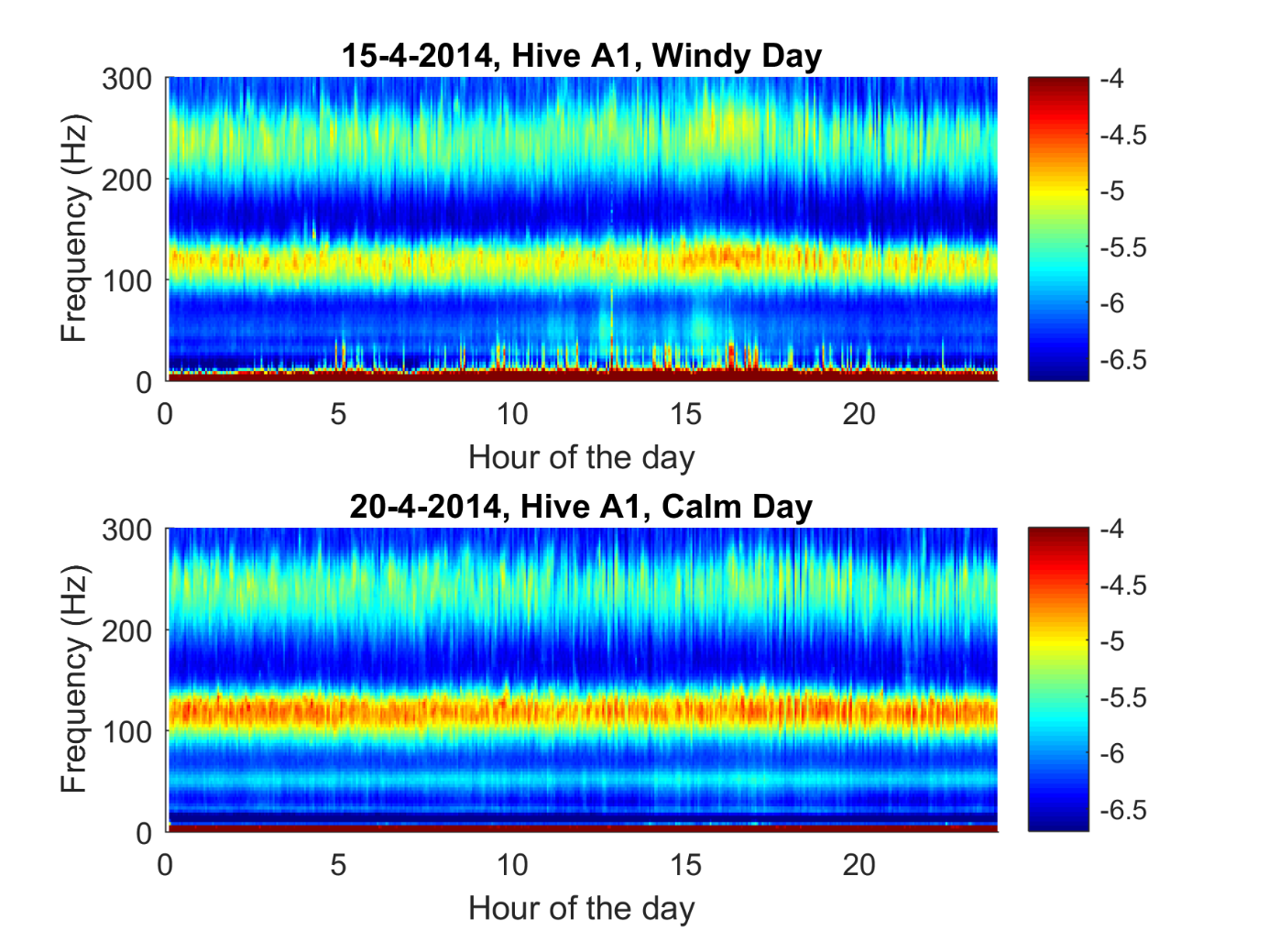


Figure S75: **Daily spectrogram of a windy day compared to that of a calm day,** as captured within the 3- minute averaged spectrum of vibration. The data from these plots were extracted from the 2014 dataset pertaining to 2014 French Hive 1.

Demonstrated in Fig S75 is the comparison of two days within the 2014 dataset of Hive A1, one of which experienced high levels of wind and the other in which the weather was calm. The onsite weather station at INRA, France, recorded wind speeds of up to 17 m/s (mean: 14.125 m/s) on the 15^th^ April 2014, compared to a maximum of 5 m/s (mean: 2.875 m/s) on the 20th April. Within the spectrograms that make up Fig S75, large peaks exist between 0 and 18Hz in the image for the 15th April (top) that are absent from the plot for the 20th April. This phenomena simultaneously appears across all hives in our dataset, and given the results for wind speeds presented in Fig S76, it can be concluded that this enhanced low frequency spectra is attributed to the accelerometers detection of the increased wind.


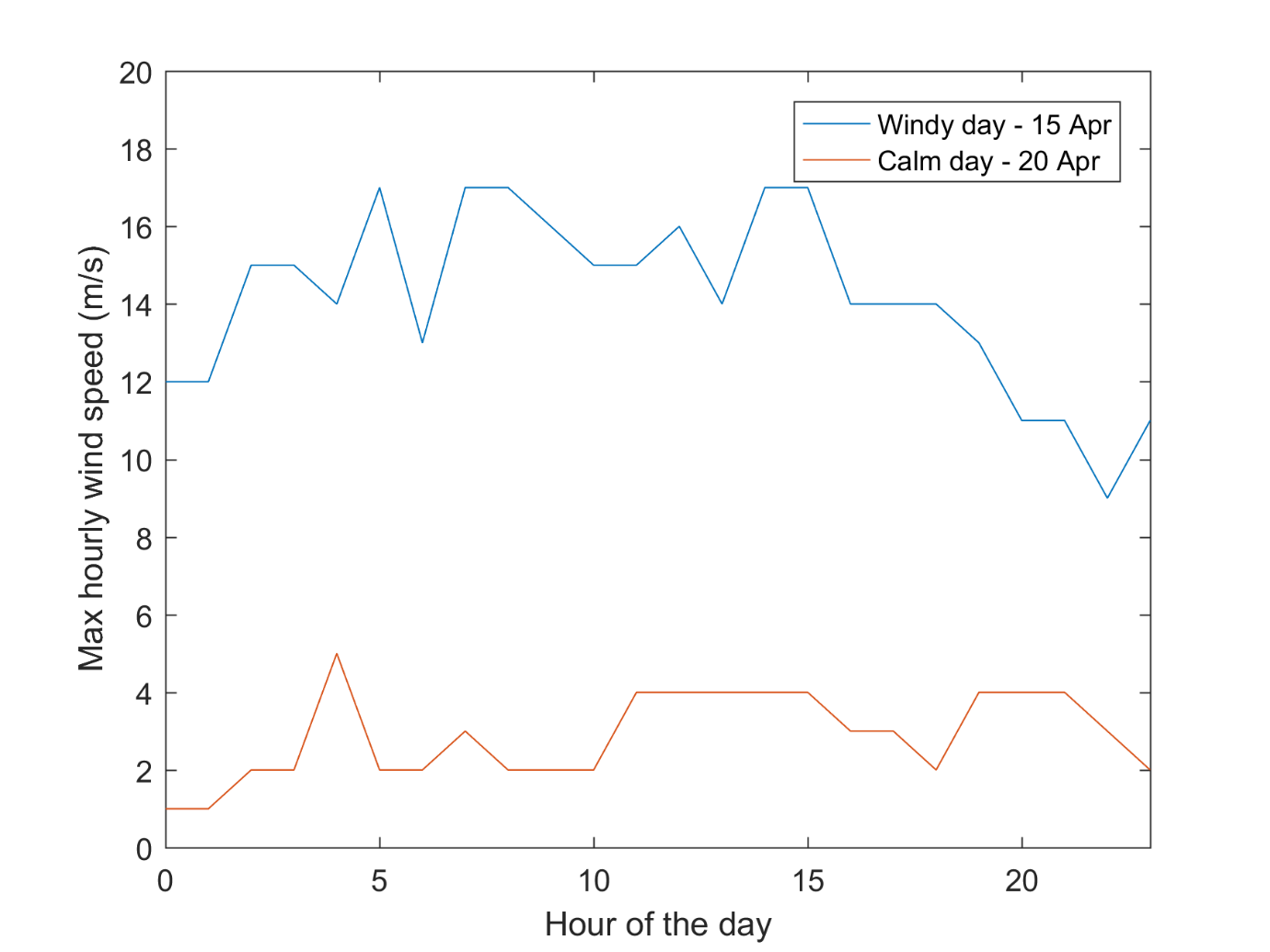


Figure S76: **Analysis of wind speeds for the windy and calm days exhibited in Fig S75.**
